# Capsaicin acts as a novel NRF2 agonist to suppress ethanol induced gastric mucosa oxidative damage by directly disrupting the KEAP1-NRF2 interaction

**DOI:** 10.7554/eLife.97632

**Published:** 2026-06-09

**Authors:** Xiaoning Gao, Wuyan Guo, Peiyuan Liu, Mingyue Yuwen, Hongyu Ren, Shengtao Hu, Zixiang Liu, Ruyang Tan, Kairui Liu, Zhiru Yang, Junli Ba, Xue Bai, Shiti Shama, Cong Tang, Kai Miao, Haozhi Pei, Liren Liu, Cheng Zhu, Tao Wang, Bo Zhang, Jun Kang

**Affiliations:** 1 School of Life Sciences, Tianjin University Tianjin China; 2 Tianjin JiAnKang Bio and TCM-technology Development Co, Ltd Tianjin China; 3 CAS and THHDG (Tianjin) Rural Revitalization Industry Development Co, Ltd Tianjin China; 4 Peiyang Park Campus, Tianjin University Tianjin China; 5 Department of Molecular Pharmacology, Tianjin Medical University Cancer Institute & Hospital; National Clinical Research Center for Cancer; Key Laboratory of Cancer Prevention and Therapy, Tianjin; Tianjin's Clinical Research Center for Cancer Tianjin China; 6 Institute for TCM-X, MOE Key Laboratory of Bioinformatics, Bioinformatics Division, BNRist, Department of Automation, Tsinghua University Beijing China; University of Oxford United Kingdom; National Cancer Institute United States

**Keywords:** oxidative stress, KEAP1, capsaicin, allosteric regulation, Mouse

## Abstract

Excessive alcohol consumption poses significant health risks and is closely associated with oxidative damage. The KEAP1-NRF2-ARE signaling pathway serves as the primary antioxidant system. However, current small molecule inhibitors are all covalently bound to KEAP1, meaning that once bound, they are not easily dissociated, while continuous inhibition of KEAP1 exhibits severe side effects. In this study, BLI, CETSA, Pull-down, Co-IP, and HDX-MS assay analysis were conducted to detect the KEAP1 binding behavior of natural product, capsaicin (CAP), both in vitro and in cells. The ethanol-induced acute gastric mucosal damage rat model was also established to evaluate the therapeutic effect of CAP. Our findings demonstrated that CAP mitigated mitochondrial damage, facilitated the nuclear translocation of NRF2, leading to the up-regulation of downstream antioxidant response elements, HMOX1, TXN, GSS, and NQO1 in GES-1 cells. Furthermore, CAP directly bind to KEAP1 and inhibit the interaction between KEAP1 and NRF2. In the KEAP1-knockout 293T cells, CAP failed to activate NRF2 expression. We identified that CAP non-covalently bound to the Kelch domain and allosterically regulated three specific regions of KEAP1: L342-L355, D394-G423, and N482-N495. To improve drug solubility and delivery efficiency, we developed IR-Dye800 modified albumin-coated CAP nanoparticles. The nanoparticles significantly reduced the gastric mucosal inflammation and activated NRF2 downstream genes in vivo. Our hypothesis was further verified our hypothesis in Nrf2-knockout mice. This study provides new insights that CAP is a safe and novel NRF2 agonist by allosterically regulating KEAP1, which may contribute to the development of lead drugs for oxidative stress-related illness, e.g., aging, cancer, neurodegenerative, and cardiovascular diseases.

## Introduction

Ethanol (EtOH), commonly referred to as alcohol in everyday life, is a fascinating ingredient found in beer, wine, and liquor. Excessive consumption poses great health risks and is responsible for approximately 3 million deaths per year (World Health Organization, 2018). In 2016, the largest share of all deaths caused by alcohol consumption worldwide was attributed to digestive disorders (21.3%) (World Health Organization, 2018). Alcohol induces oxidative damage to gastric mucosa. Persistent and intensified oxidative stress, particularly reactive oxygen species (ROS), induces DNA damage, protein oxidation, and lipid peroxidation (Zhang et al., 2020). Consequently, this can result in gastrointestinal dysfunction, chronic atrophic gastritis, and is closely related to the development of gastric cancer (Yu et al., 2022).

Natural products are essential sources of drugs or lead compounds for treating major diseases (Srinivasan, 2014). Previous reports indicate that the incidence rate of gastric ulcers in Indians and Malaysians is quite low. Chili peppers are commonly found in the cuisine of these two countries (Satyanarayana, 2006). This raises questions regarding the potential for chili peppers to alleviate gastric mucosal damage and reduce the occurrence of gastric ulcers. Recent experiments conducted in rats have demonstrated that red pepper/capsaicin (CAP) possesses significant protective effects on ethanol-induced gastric mucosal damage, and the mechanisms involved may relate to the promotion of vasodilation (Holzer et al., 1991; Raimura et al., 2013), increased mucus secretion (Kang et al., 1995),and the release of calcitonin gene-related peptide (CGRP) (Hayashi et al., 2001; Saeki et al., 2000). However, it is important to note that the specific role of the antioxidant activity of CAP has not been thoroughly investigated.

The Kelch-like ECH-associated protein 1 (KEAP1)–Nuclear factor erythroid 2–related factor 2 (NRF2)–antioxidant response element (ARE) pathway is a core defense mechanism against oxidative and electrophilic stress (Yamamoto et al., 2018). Under homeostatic conditions, KEAP1 acts as a linker protein for the Cul3-E3 ubiquitin ligase complex, continuously promoting the ubiquitination and proteasomal degradation of NRF2, thereby maintaining NRF2 at basal levels (McMahon et al., 2006). When oxidative or electrophilic stress occurs, critical cysteine residues in KEAP1 are modified, or the interaction between the ETGE/DLG motifs on NRF2 and the Kelch domain of KEAP1 is disrupted, allowing NRF2 to escape degradation, accumulate, and translocate to the nucleus. There, NRF2 forms heterodimers with small Maf proteins and binds to ARE, inducing the expression of antioxidant and cytoprotective genes, such as those involved in glutathione metabolism, NADPH regeneration, phase II detoxifying enzymes, and drug efflux transporters, thereby restoring redox balance within the cell and reducing oxidative damage (Forman and Zhang, 2021).

Classical NRF2 agonists, such as sulforaphane, are small molecules that bind to KEAP1 and covalently modify its cysteine residues, thereby altering the binding affinity between KEAP1 and NRF2 (Zhang and Hannink, 2003). However, traditional covalent agonists may induce sustained overactivation of NRF2, leading to adverse side effects and limiting clinical application (Liu et al., 2019). Consequently, recent efforts have shifted toward the development of non-covalent NRF2 agonists, which are generally associated with lower toxicity and greater translational potential, enabling more controlled enhancement of NRF2 activity and offering new insights and therapeutic opportunities in antioxidant-related interventions.

In this study, EtOH-induced acute oxidative damage models were established in vitro and in vivo, and the effects of CAP pretreatment on KEAP1-NRF2-ARE signaling pathway were verified. Ultimately, we employed hydrogen-deuterium exchange mass spectrometry (HDX-MS) to identify the allosteric regulatory sites following CAP treatment. CAP may emerge as a pioneering direct inhibitor of the KEAP1-NRF2 interaction, which also offers an in-depth understanding of its antioxidative defense mechanism.

## Results

### CAP protects GES-1 from oxidative stress

The molecular formula of CAP (PubChem CID: 1548943) is provided in Figure 1—figure supplement 1a. Initially, an oxidative stress model was established in human gastric mucosal epithelial (GES-1) cells. According to the *Lancet* (Wood et al., 2018), WHO recommendation and US Standard Drink Sizes, that daily alcohol consumption should be limited to 1–2 standard cups/day (1 standard cup is equivalent to 10–14 g of pure EtOH in different countries), so cell models were tested at a similar concentration of 5%. A dose-dependent decline in cell viability upon exposure to EtOH was observed, with viability decreasing to approximately 50% after 1.5 hr of treatment with 5% EtOH (Figure 1—figure supplement 1b). To evaluate the protective effects of CAP, GES-1 model cells were pretreated with CAP for 2.5 hr before introducing EtOH. The results revealed that a pretreatment with 8 μM CAP significantly enhanced cell viability to 93.84% (Figure 1—figure supplement 1c). Notably, further increases in CAP concentration did not enhance the protective effect, possibly due to cytotoxicity at higher concentrations (Thongin et al., 2022; Figure 1—figure supplement 1d). Subsequently, three CAP concentrations (2, 8, and 32 μM) and three EtOH concentrations (0.5%, 3.5%, and 5%) on GES-1 were further valuated to establish a stable cell model in vitro (Figure 1—figure supplement 1e–1g). Furthermore, we assessed EtOH-induced apoptosis and found that the percentage of apoptotic cells was reduced from 28.85–13.95% in the group pretreated with 8 μM CAP, compared to the EtOH-only group (Figure 1—figure supplement 1h).

**Figure 1.**
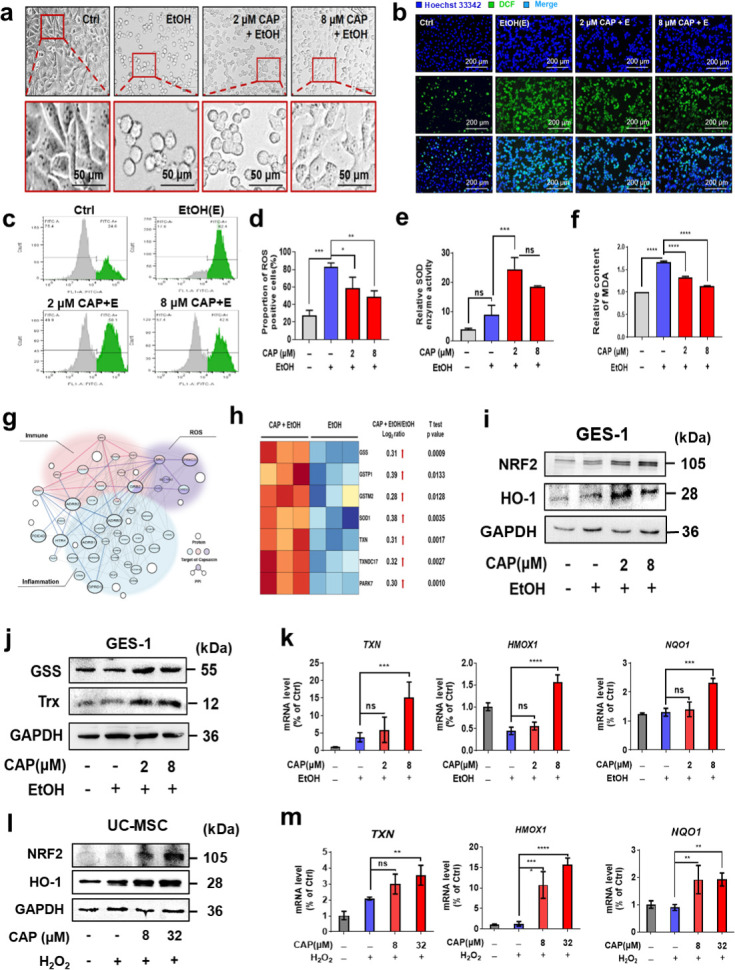
Capsaicin (CAP) reduces reactive oxygen species (ROS) and promotes nuclear factor erythroid 2–related factor 2 (NRF2) expression. (**a**) Microscopic examination of gastric mucosal epithelial cells (GES-1) cellular morphology following treatments. Cells were pre-treated with CAP at concentrations of 2 or 8 μM for 2.5 hr, followed by incubation with 5% ethanol (EtOH) for 1.5 hr. Scale bar, 50 μm. (**b**) Detection of ROS-positive GES-1 cells using DCFH-DA staining. Scale bar, 200 μm. (**c**) Flow cytometric (FCM) analysis of ROS-positive GES-1 cells labeled with DCFH-DA. (**d**) Statistical analysis of ROS levels measured by FCM. The experiment was conducted in triplicate. (**e**) Assessment of superoxide dismutase (SOD) activity in GES-1 cells. (**f**) Quantification of malondialdehyde (MDA) levels of GES-1 cells. (**g**) A network targets of CAP. Purple, blue, pink nodes represent proteins or genes in the predicted biological effect profile of CAP related to ROS, inflammation, or immune, respectively. (**h**) Heatmap depicting differentially expressed genes (DEGs) enriched in antioxidant activity based on proteomic analysis. Each row represents the expression level of a single gene, while each column corresponds to an individual experimental sample. (**i**) Western blot analysis of NRF2 and heme oxygenase 1 (HO-1) expression in GES-1 cells. (**j**) Western blot assessment of antioxidant protein expression in GES-1 cells. (**k**) Quantitative analysis of relative mRNA levels of antioxidant response elements (ARE)-related genes in GES-1 cells. Cells were treated with or without CAP and EtOH, and the mRNA levels of thioredoxin (TXN), HMOX1, and quinone dehydrogenase 1 (NQO1) were measured. Each group consisted of three replicates. (**l**) Western blot analysis of NRF2 and HO-1 expression in human umbilical cord mesenchymal stem cells (UC-MSC). (**m**) Quantitative analysis of relative mRNA levels of ARE-related genes in HUC-MSCs. Figure 1—source data 1.TIFF files that contain original western blots indicating the relevant bands and treatments. Figure 1—source data 2.Original files for western blot analysis.

Then, the morphological changes in GES-1 cells were examined. Cells in the EtOH-treated group displayed a rounded morphology and showed a marked tendency for aggregation. In contrast, cells that were pretreated with 2 μM and 8 μM CAP largely retained their characteristic cellular shape (Figure 1a). Then, we assessed ROS production using DCFH-DA staining. The EtOH-only group exhibited elevated levels of ROS, as indicated by increased green fluorescence, while the phenomenon was substantially diminished in CAP-pretreated groups (Figure 1b). Flow cytometric analyses revealed a consistent decline in intracellular ROS levels with increasing dosages of CAP: from 83.1 ± 4.39% in the ethanol-only group to 58.6 ± 12.64% and 48.9 ± 6.74% in the 2 μM and 8 μM CAP groups, respectively (Figure 1c and d). To evaluate the impact of CAP on intracellular redox homeostasis, the activity of superoxide dismutase (SOD), as well as the amount of malondialdehyde (MDA) were measured. Our findings demonstrated that SOD levels were significantly upregulated following CAP treatment (Figure 1e), while MDA production was markedly reduced (Figure 1f). Collectively, these results suggest that CAP effectively reduces ROS levels and enhances redox balance in GES-1 cells exposed to EtOH.

**Figure 1—figure supplement 1.**
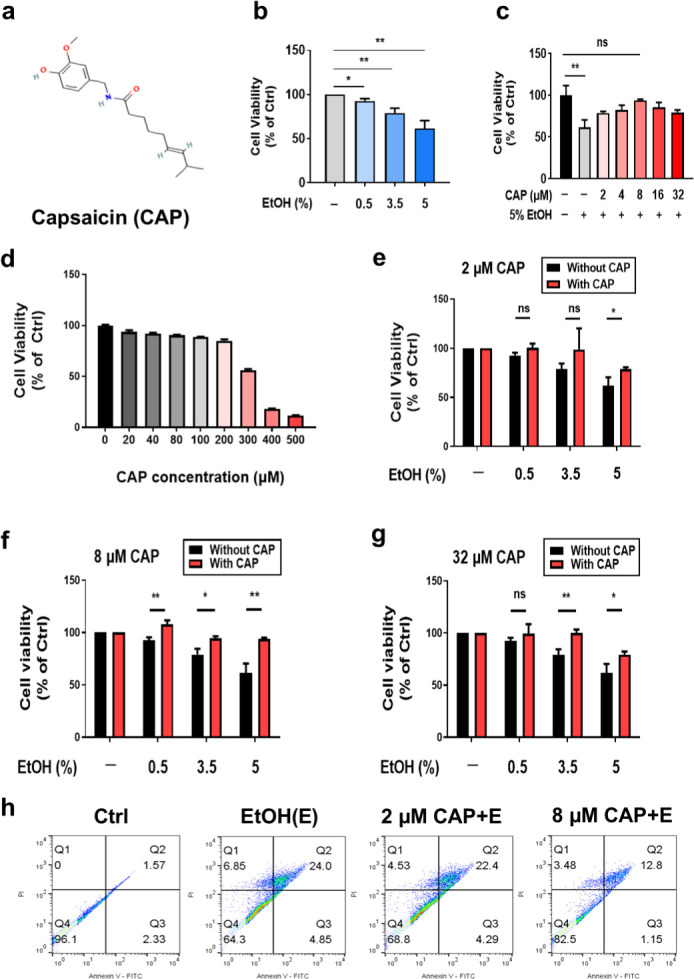
Capsaicin (CAP) protects gastric mucosal epithelial cells (GES-1) from oxidative stress. (**a**) Molecular formula of CAP from PubChem (Compound CID: 1548943). (**b**) Cell viability of GES-1 after 1.5 hr treatment with ethanol at different concentrations (v/v). (**c**) Cell viability of GES-1 after pretreatment with different concentrations of CAP for 2.5 hr and adding 5% ethanol for 1.5 hr. (**d**) Effects of different concentrations of CAP on the viability of GES-1 cells for 24 hr. (**e–g**) Cell viability was measured by adding CAP (2 μM, 8 μM, or 32 μM) to GES-1 cells for 2.5 hr and then adding three concentrations of ethanol (0.5%, 3.5%, and 5%) for another 1.5 hr. (**h**) Flow cytometric (FCM) showed CAP inhibited the apoptosis of GES-1 induced by ethanol (EtOH).

**Figure 1—figure supplement 2.**
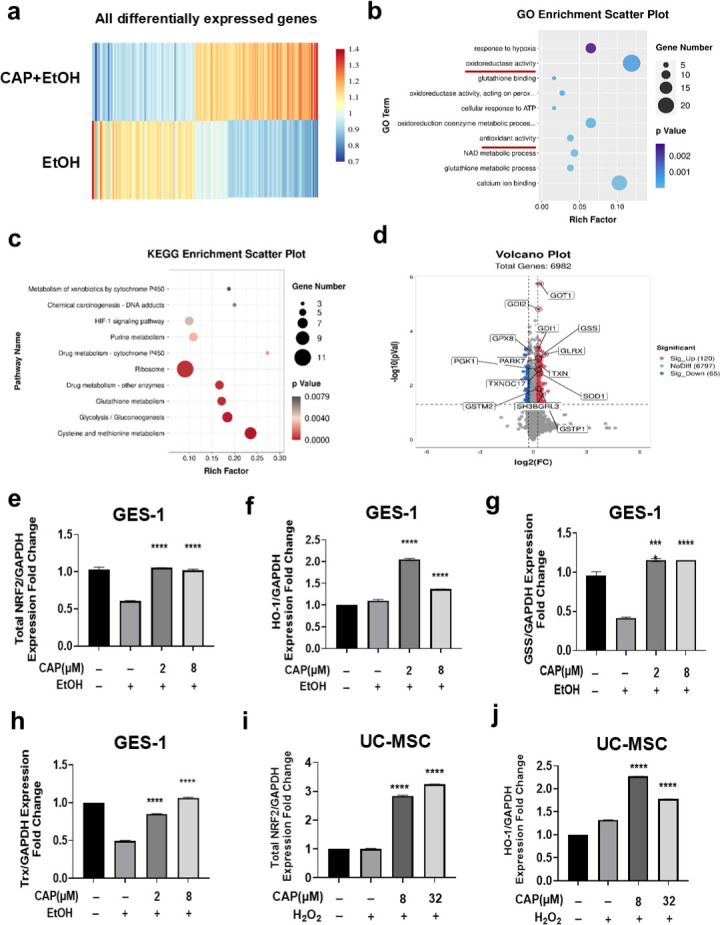
Potential pathways and proteins that capsaicin (CAP) may affect. (**a**) Heat map showed the up-regulation and down-regulation of all differentially expressed proteins in CAP (8 μM, 2.5 hr) and 5% ethanol (EtOH) (1.5 hr) versus 5% EtOH (1.5 hr) alone. Red and blue are indicative of increased and decreased expression, respectively. (**b**) Gene Ontology (GO) enrichment analysis of differentially expressed genes (DEGs). (**c**) Kyoto Encyclopedia of Genes and Genomes (KEGG) enrichment analysis of differentially expressed genes (DEGs). (**d**) Volcano plot illustrating DEGs between two treatment groups in gastric mucosal epithelial cells (GES-1) cells. Genes with significantly increased expression were marked in red, while those with significantly decreased expression were marked in blue. (**e–h**) Grayscale analysis of western blotting (WB) in GES-1. Statistical results of the expression of total nuclear factor erythroid 2–related factor 2 (Nrf2), heme oxygenase 1 (HO-1), glutathione synthase (GSS), and thioredoxin (Trx) compared with internal reference protein GAPDH. Experiments were repeated three times. (**i and j**) Grayscale analysis of western blot in HUC-MSC. Statistical results of the expression of total Nrf2, HO-1, GSS, and Trx compared with internal reference protein GAPDH.

### CAP activates the NRF2-ARE signaling pathway

With analytical techniques, such as network pharmacology (Li, 2021) and single-cell sequencing, researchers identified pivotal target proteins implicated in the precursor lesions of gastric cancer (Zhang et al., 2019a). Based on the network targets analysis, CAP may regulate pathways and biological processes related to ROS, inflammation and immune expression (Figure 1g). However, the specific mechanism of ROS regulation by CAP remains unclear, so we conducted proteomic analyses to identify differentially expressed genes (DEGs). A comprehensive overview of these DEGs was presented in the heat map. Using stringent criteria for statistical significance (|Fold Change|>1.2, p-value<0.05), analysis revealed that 120 proteins were significantly up-regulated, while 65 were down-regulated following CAP treatment (Figure 1—figure supplement 2a). Subsequent Gene Ontology (GO), and Kyoto Encyclopedia of Genes and Genomes (KEGG) enrichment analyses were performed on this pool of 185 DEGs (Figure 1—figure supplement 2b and c). As anticipated, the top terms in the ‘Molecular Function’ category of the GO analysis included ‘antioxidant activity’ (GO: 0016209) and ‘oxidoreductase activity’ (GO: 0016491). Genes corresponding to these functional categories were highlighted in the volcano plot (Figure 1—figure supplement 2d) and annotated in the heat map (Figure 1h). Intriguingly, our findings demonstrated a significant elevation in the expression of key antioxidant-related proteins, such as glutathione synthase (GSS), superoxide dismutase 1 (SOD1), and thioredoxin (TXN, also known as Trx). These proteins were associated with previously reported antioxidant response elements (AREs) and function as downstream targets of the transcription factor NRF2 (Ngo and Duennwald, 2022). We hypothesize that CAP may activate the NRF2-ARE signaling pathway in oxidative stress models.

To substantiate the hypothesis, we discovered that CAP significantly increased the expression levels of NRF2 and heme oxygenase 1 (HMOX1, also known as HO-1) in GES-1 cells (Figure 1i), which is a downstream target of NRF2. Concurrently, the elevated expression levels of GSS and Trx proteins, which were also downstream targets of NRF2, were further validated by western blotting (Figure 1j). A grayscale quantification of these pivotal proteins was presented in Figure 1—figure supplement 2e–2h. Recognizing NRF2’s role as a transcriptional regulator, we further analyzed the transcript levels of these essential target genes: TXN, HMOX1, and NQO1. It’s worth mentioning that NAD(P)H quinone dehydrogenase 1 (NQO1) is also a classic target gene of NRF2 (Xiang et al., 2022). Quantitative reverse transcription PCR (RT-qPCR) assays demonstrated that CAP significantly increased the transcription of these antioxidant-associated genes (Figure 1k).

In a parallel experiment involving a hydrogen peroxide (H_2_O_2_)-induced oxidative stress model in human umbilical cord mesenchymal stem cells (UC-MSC), CAP pretreatment similarly boosted the transcription and protein expression levels of NRF2 and its downstream genes of TXN, HMOX1, and NQO1 (Figure 1l and m). A grayscale quantification of these pivotal proteins was also shown in Figure 1—figure supplement 2i and j. These collective findings suggested that CAP’s role in ROS mitigation was not confined to a specific cell type, needing further exploration of the underlying mechanisms.

### CAP promotes NRF2 to translocate into nucleus

**Figure 2.**
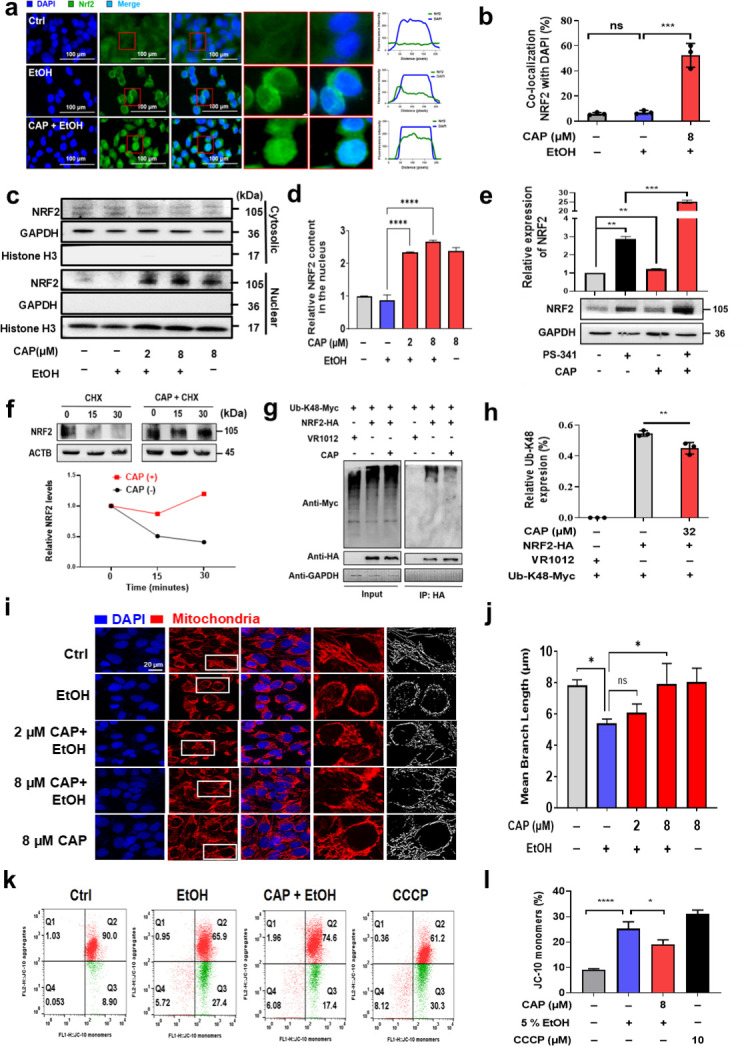
Capsaicin (CAP) inhibits the ubiquitination and degradation of nuclear factor erythroid 2–related factor 2 (NRF2). (**a**) Immunofluorescence detection of NRF2 nuclear localization DAPI was employed to label the cell nuclei for reference. Scale bar, 100 μm. (**b**) Statistical analysis of NRF2 nuclear translocation following 8 μM CAP pre-treatment. The proportion of NRF2 localized in the nucleus post-CAP treatment was quantitatively assessed. (**c and d**) Subcellular localization of NRF2 in gastric mucosal epithelial cells (GES-1) cells across different treatment groups. NRF2 levels in both the nucleus and cytoplasm were assessed. GAPDH was used as a cytoplasmic marker, and Histone H3 served as a nuclear marker. Statistical analysis was performed specifically on the nuclear localization of NRF2. (**e**) Total NRF2 levels induced by PS-341 or CAP. (**f**) Analysis of total NRF2 levels under the influence of cycloheximide (CHX), with or without 8 μM CAP treatment. NRF2 degradation was semi-quantitatively assessed using ImageJ software to analyze the western blot results. (**g–h**) Inhibition of K48 ubiquitination on NRF2 protein by 32 μM CAP as assessed by Co-IP assay in 293T cells. (**i**) Mitochondrial visualization in GES-1 cells following CAP and ethanol (EtOH) treatment regimens. Cells were pre-treated with CAP at concentrations of 2 or 8 μM for 2.5 hr, followed by a 10 min incubation with 5% EtOH. Mitochondria were labeled with Mito Tracker Red CMXRos and detected via the Leica STELLARIS 5 Confocal Microscope Platform. Red fluorescence indicates the mitochondria, while blue fluorescence (DAPI staining) marks the cell nucleus. Scale bar represents 20 μm. (**j**) Quantitative analysis of mitochondrial branch length in different treatment groups using ImageJ and GraphPad Prism. The branch length of individual mitochondria was analyzed using ImageJ software and the data were plotted using GraphPad Prism. (**k**) Assessment of mitochondrial membrane potential (ΔΨm) in GES-1 cells using flow cytometric (FCM). Cells were pre-treated with CAP at concentrations of 2 or 8 μM for 2.5 hr, followed by a 10 min incubation with 5% EtOH or a 20 min incubation with CCCP as a positive control. ΔΨm was evaluated using JC-10 staining. (**l**) Quantitative analysis of ΔΨm across different treatment groups . Figure 2—source data 1.TIFF files that contain original western blots indicating the relevant bands and treatments. Figure 2—source data 2.Original files for western blot analysis.

Furthermore, we proceeded to investigate the subcellular localization of NRF2 following pretreatment with CAP. Our findings revealed that CAP effectively facilitated the translocation of NRF2 into the nucleus. Specifically, 52.29% of NRF2 fluorescence co-localized with the nuclear dye DAPI, as determined by analyses across three randomly selected fields of view for each sample group (Figure 2a and b). To corroborate this observation, we conducted a nucleoplasm separation assay to quantify the expression levels of NRF2 in both the nuclear and cytoplasmic compartments (Figure 2c). Grayscale analysis of the immunoblotting data further corroborated that the nuclear accumulation of NRF2 increased with CAP treatment (Figure 2d).

**Figure 2—figure supplement 1.**
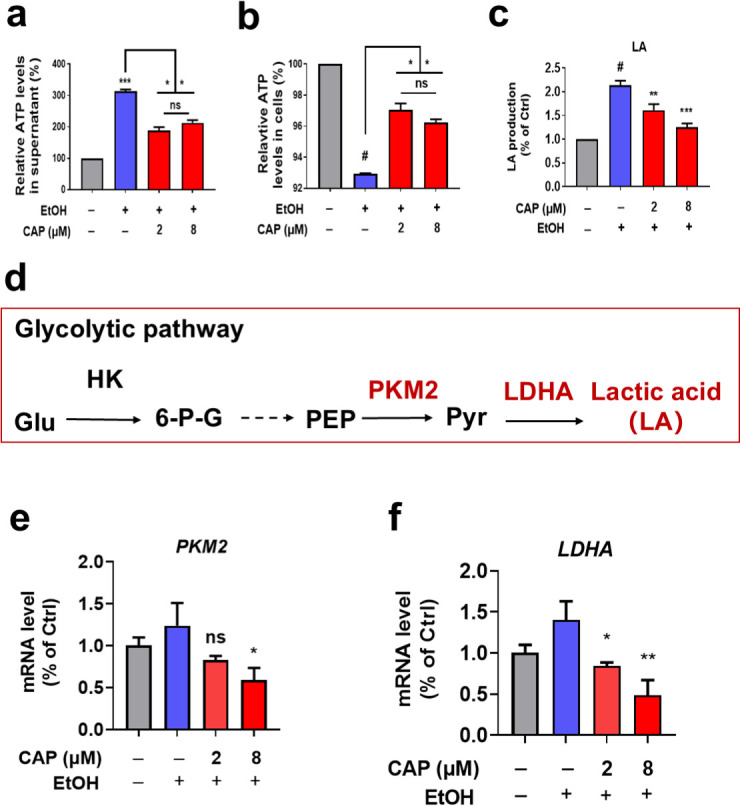
Capsaicin (CAP) increases ATP content and inhibits lactic acid (LA) production. (**a**) The content of ATP in gastric mucosal epithelial cells (GES-1) cells in different treatment groups. (**b**) CAP (8 μM) pretreatment for 2.5 hr and 5% ethanol (EtOH) incubation for 1.5 hr. (c) The intracellular LA level was measured. (d) Schematic diagram of intracellular glycolysis. (**e and **f) Detection of the mRNA level of *PKM2* and *LDHA* in different treatment groups.

### CAP inhibits the degradation of NRF2

NRF2 was known to undergo proteasomal degradation, so we employed PS-341, a renowned proteasome inhibitor, to selectively impede the 20 S proteasome activity. Our data revealed that PS-341 independently elevated NRF2 expression levels. Moreover, when cells were co-incubated with both PS-341 and CAP, the overall NRF2 levels rose even further, which may suggest that the mechanism of CAP differs from that of PS-341 (Figure 2e).

To delve deeper into whether CAP affects protein degradation, we utilized cycloheximide (CHX), a recognized inhibitor of protein synthesis (Liu et al., 2022). As shown in Figure 2f, CHX treatment led to a rapid decline in NRF2 protein levels, rendering it nearly undetectable approximately 30 min after treatment. Conversely, NRF2 levels remained noticeably stable when co-incubated with CAP, suggesting that CAP prolongs NRF2 half-life. These findings support the idea that CAP enhances NRF2 stability by inhibiting its proteolytic degradation. In alignment with existing literature, which posits that K48 ubiquitination facilitates NRF2 degradation (Hong et al., 2023; Zhang et al., 2019b), we carried out ubiquitin immunoprecipitation experiments. These indicated a modest reduction, approximately 17.57%, in K48 ubiquitination of NRF2 following CAP treatment (Figure 2g and h). This phenomenon indicated that there may be other ways to inhibit degradation.

### CAP protects mitochondrial function

The mitochondria serve as the primary intracellular source of ROS (Dan Dunn et al., 2015). Initial exploratory studies focused on the role of NRF2 in maintaining mitochondrial integrity (Esteras and Abramov, 2022). Our findings revealed a 30.93% decrease in average mitochondrial branch length after treatment with EtOH. Remarkably, pretreatment with CAP was found to preserve mitochondrial structural integrity in GES-1 cells (Figure 2i and j). We subsequently turned our attention to the mitochondrial membrane potential (MMP, ΔΨm), a key factor in sustaining mitochondrial oxidative phosphorylation and the subsequent production of adenosine triphosphate (ATP). Exposure to 5% EtOH for 10 min or the positive control CCCP for 20 min resulted in comparable declines in ΔΨm, affecting approximately 30% of the cells. Notably, pretreatment with 8 μM CAP significantly mitigated this reduction in ΔΨm (Figure 2k and l).

Typically, diminished levels of ATP serve as a hallmark of compromised mitochondrial function. To investigate this, we employed a luminescence detector to quantify both extracellular and intracellular ATP concentrations. The extracellular ATP levels surged to approximately three times in the EtOH-only group compared to the control, while a corresponding sharp decrease was observed in intracellular ATP concentrations (Figure 2—figure supplement 1a). In striking contrast, CAP pretreatment effectively stabilized intracellular ATP levels, maintaining them at 96.24% of their original concentration (Figure 2—figure supplement 1b). Building on these observations, we assessed the end product of the anaerobic glycolytic pathway, lactate (LA), as well as the transcription levels of key metabolic enzymes—pyruvate kinase M2 (PKM2) and lactate dehydrogenase A (LDHA). CAP treatment resulted in significant downregulation of both LA and the aforementioned enzymes, suggesting a potential metabolic reprogramming from glycolysis to oxidative phosphorylation (Figure 2—figure supplement 1c–1f). Previous studies have demonstrated that CAP can directly bind to and inhibit the activity of PKM2 and LDHA, subsequently attenuating inflammatory responses (Zhang et al., 2022b). Therefore, our findings underscore the role of CAP in maintaining mitochondrial function and also reducing oxidative stress.

### CAP disrupts KEAP1-NRF2 interaction

**Figure 3.**
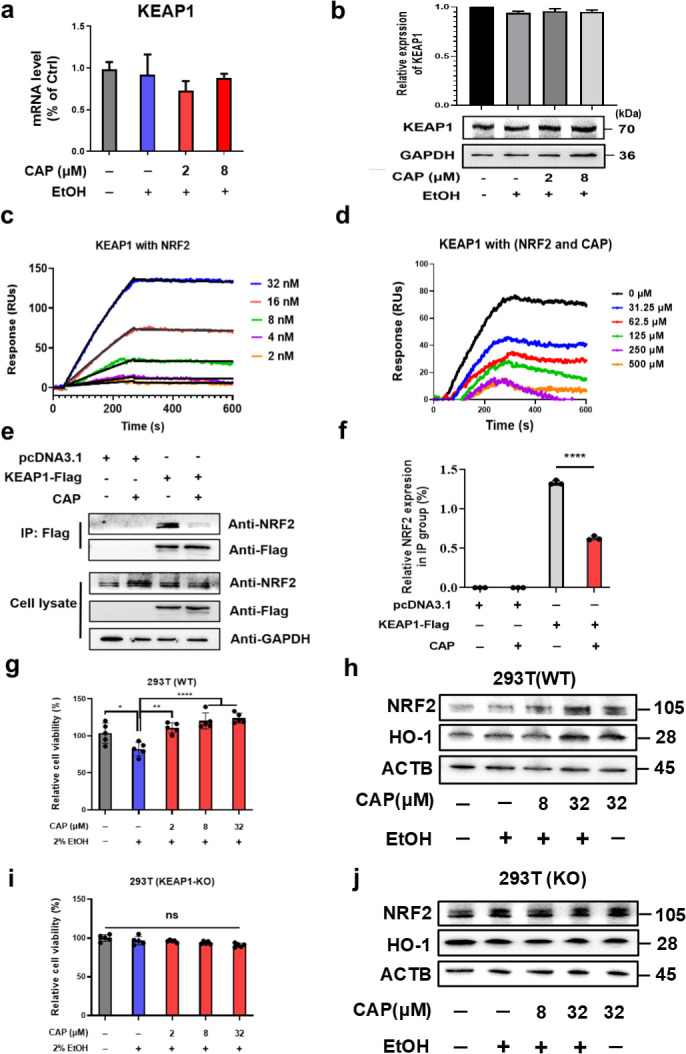
Capsaicin (CAP) disrupted KEAP1-NRF2 interaction. (**a–b**) Assessment of KEAP1 transcription and expression levels using RT-qPCR and western blot analyses. (**c**) KEAP1-NRF2 interaction was detected with Surface plasmon resonance (SPR) in vitro. (**d**) Disruption of KEAP1-NRF2 interaction by CAP as assessed by SPR. (**e**) Disruption of KEAP1-NRF2 interaction by 32 μM CAP as assessed by Co-Immunoprecipitation (Co-IP) in 293T Cells. (**f**) Quantitative analysis of relative nuclear factor erythroid 2–related factor 2 (NRF2) expression in IP samples using ImageJ software. (**g**) Cell viability assessment of 293T cells treated with CAP and EtOH using CCK-8 assay. (**h**) Western blot analysis of NRF2 and HO-1 expression in 293T cells. (**i**) Cell viability assessment of 293T(KO) cells treated with CAP and ethanol (EtOH) using CCK-8 assay. (**j**) Western blot analysis of NRF2 and heme oxygenase 1 (HO-1) expression in 293T(KO) cells. Figure 3—source data 1.TIFF files that contain original western blots indicating the relevant bands and treatments. Figure 3—source data 2.Original files for western blot analysis.

To explore the possible influence of CAP on NRF2 stabilization via KEAP1 modulation, our findings revealed no significant alterations in KEAP1 expression, both at the transcriptional level (Figure 3a) and at the protein level (Figure 3b). The possibility that CAP exerted no impact on KEAP1 expression suggests the likelihood that CAP may disrupt the interaction between KEAP1 and NRF2.

**Figure 3—figure supplement 1.**
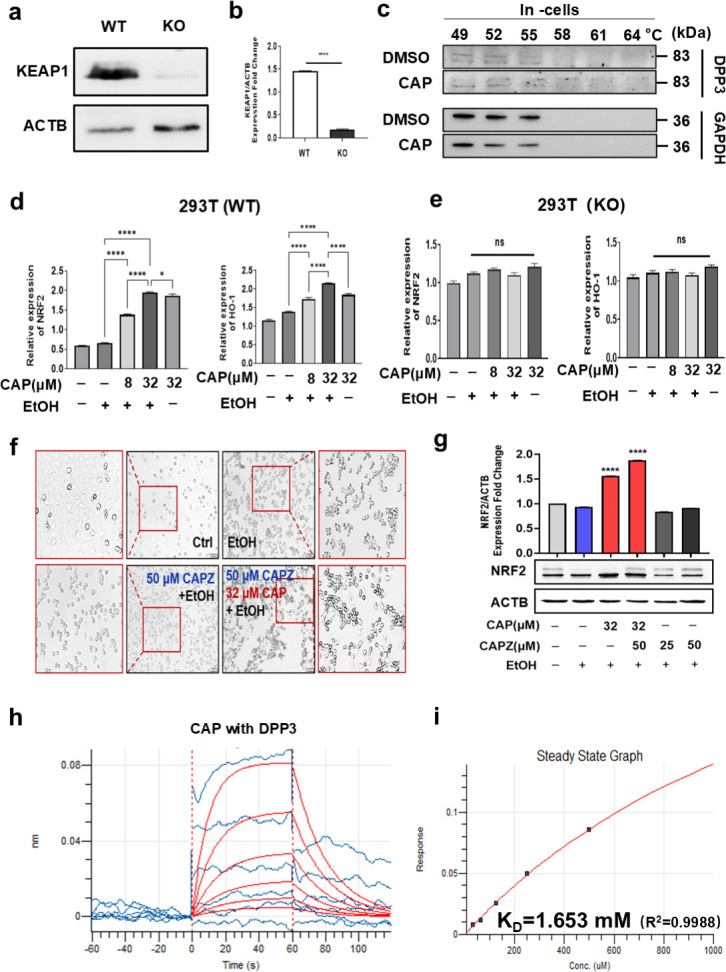
Capsaicin (CAP) protects 293T from oxidative stress. (**a and b**) Expression of KEAP1 in human KEAP1 knockout 293T cells (293T KO). (**c**) Detection of DPP3-CAP interaction in gastric mucosal epithelial cells (GES-1) using cellular thermal shift assay coupled with western blotting (CETSA-WB). (**d**) The gray analysis of nuclear factor erythroid 2–related factor 2 (NRF2) and HO-1 of each group in 293T. (**e**) The gray analysis of NRF2 and heme oxygenase 1 (HO-1) of each group in 293T(KO). (**f**) Microscopic examination of GES-1 cellular morphology following treatment regimens. Cells were pre-treated with CAP or Capsazepine (CAPZ) for 2.5 hr, followed by incubation with 5% ethanol (EtOH) for 1.5 hr. Scale bar, 10 μm. (**g**) Western blot analysis of NRF2 protein levels under varying concentrations of CAP and CAPZ, with or without EIOH treatment. (**h and i**) In vitro detection of DPP3 with CAP using bio-layer interferometry assay (BLI). Figure 3—figure supplement 1—source data 1.TIFF files that contain original western blots indicating the relevant bands and treatments. Figure 3—figure supplement 1—source data 2.Original files for western blot analysis.

Surface plasmon resonance (SPR) was used to quantitatively evaluate the in vitro binding affinity between purified KEAP1 and NRF2. The observed dissociation constant (K_D_) for the KEAP1-NRF2 complex was calculated to be 1.14±0.15 nM (Figure 3c), corroborating earlier reports of a strong association between these two proteins (Yamamoto et al., 2018). Upon exposing KEAP1 to increasing concentrations of CAP—ranging from 16 μM to 500 μM—a consistent, dose-dependent decrease in KEAP1-NRF2 binding was noted (Figure 3d). We further confirmed these observations through co-immunoprecipitation (Co-IP) assays. Treatment with 32 μM CAP led to a 52.79% reduction in the endogenous NRF2 associated with KEAP1 in cellular models (Figure 3e and f). Collectively, these results provide the first empirical evidence that CAP effectively impairs the KEAP1-NRF2 interaction.

### Knockout of KEAP1 affects the function of CAP

To investigate whether KEAP1 functions as a key target for the antioxidant properties mediated by CAP, KEAP1-knockout (KO) 293T cell lines were generated, and validation studies were performed using western blot analysis (Figure 3—figure supplement 1a and b). Initially, we observed that CAP significantly activated the NRF2 and HO-1 in wild-type 293T cells, leading to a pronounced increase in cell viability (Figure 3g and h). This finding was consistent with observations made in GES-1 and UC-MSC cells. However, in KEAP1-KO cells, an innate resilience to oxidative stress was evident, likely due to the constitutive activation of endogenous NRF2 and its downstream effector, HO-1. As a result, the CCK8 assay revealed no significant differences in cell viability between the groups (Figure 3i), corroborating previous studies that have shown KEAP1-KO mice exhibit reduced oxidative stress and inflammatory responses (Zhao et al., 2022). Importantly, in the KEAP1-KO 293T cell line, CAP displayed a reduced ability to activate NRF2 and HO-1 (Figure 3j). A grayscale quantification of these pivotal proteins was presented in Figure 3—figure supplement 1d and e. Taken together, our findings strongly support that KEAP1 is a crucial mediator of the antioxidant effects conferred by CAP and plays a pivotal role in amplifying the NRF2-ARE signaling pathway. At the same time, we also partially exclude the possibility that CAP may function through off-target effects involving the TRPV1 receptor or DPP3 with an ETGE motif (Figure 3—figure supplement 1c and f–i).

### CAP directly interacts with KEAP1

**Figure 4.**
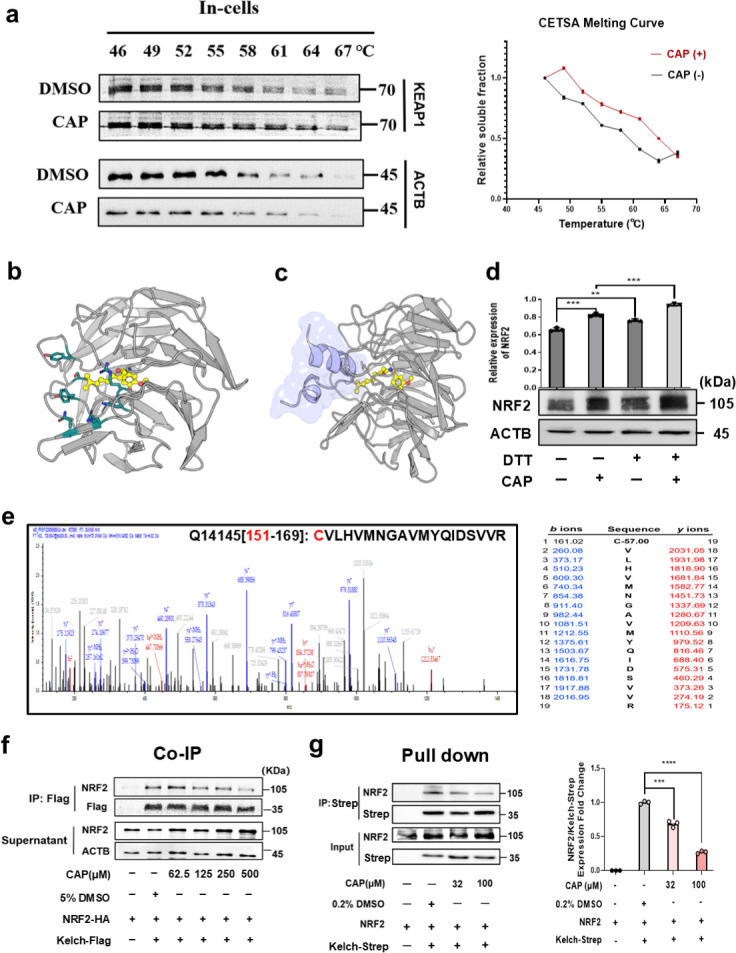
Capsaicin (CAP) specifically interacts with the Kelch domain of KEAP1. (**a**) Detection of KEAP1-CAP interaction in gastric mucosal epithelial cells (GES-1) using cellular thermal shift assay coupled with western blotting (CETSA-WB) and the melting curve generated from CETSA was analyzed using ImageJ software. The red fold line represents cells treated with CAP, while the black fold line represents cells treated with DMSO as a control. (**b**) Computational docking of CAP molecule to KEAP1 surface pockets. The Keap1 protein is represented in gray, while the CAP molecule is shown in yellow. The seven key amino acids predicted to be crucial for the interaction are highlighted in blue. (**c**) Partial overlap of CAP-binding pocket with KEAP1-NRF2 interface. The KEAP1-NRF2 interaction interface is represented in purple. (**d**) Influence of dithiothreitol (DTT) on nuclear factor erythroid 2–related factor 2 (NRF2) activation induced by CAP in GES-1 cells. Cells were pre-incubated with 400 μM DTT for 1 hr, followed by a 3 hr incubation with 8 μM CAP. (**e**) Tandem mass spectrometry (MS/MS) of analysis of KEAP1 peptide containing Cys151 following CAP treatment. (**f**) Dose-dependent examination of Kelch-NRF2 interaction in the presence of CAP in 293T cells. Cells were treated with varying concentrations of CAP (0, 62.5, 125, 250, and 500 μM) to assess the impact on the interaction between exogenously purified Kelch protein and NRF2 in the total cell lysate. (**g**) Pull-Down assay demonstrating the direct inhibition of Kelch-NRF2 Interaction by CAP. Figure 4—source data 1.TIFF files that contain original western blots indicating the relevant bands and treatments. Figure 4—source data 2.Original files for western blot analysis.

As previously documented, small molecules categorized as NRF2 agonists, such as Dimethyl fumarate (DMF), curcumin, and sulforaphane, predominantly target and inhibit the KEAP1 function (Unni et al., 2021). To probe the interplay between KEAP1 and CAP within a cellular environment, we conducted a cellular thermal shift assay (CETSA). In the absence of CAP, KEAP1 experienced marked degradation within a temperature window of 46–67°C. In contrast, the presence of CAP significantly stabilized KEAP1 against thermal denaturation, resulting in sustained levels of the KEAP1 protein in cells (Figure 4a).

**Figure 4—figure supplement 1.**
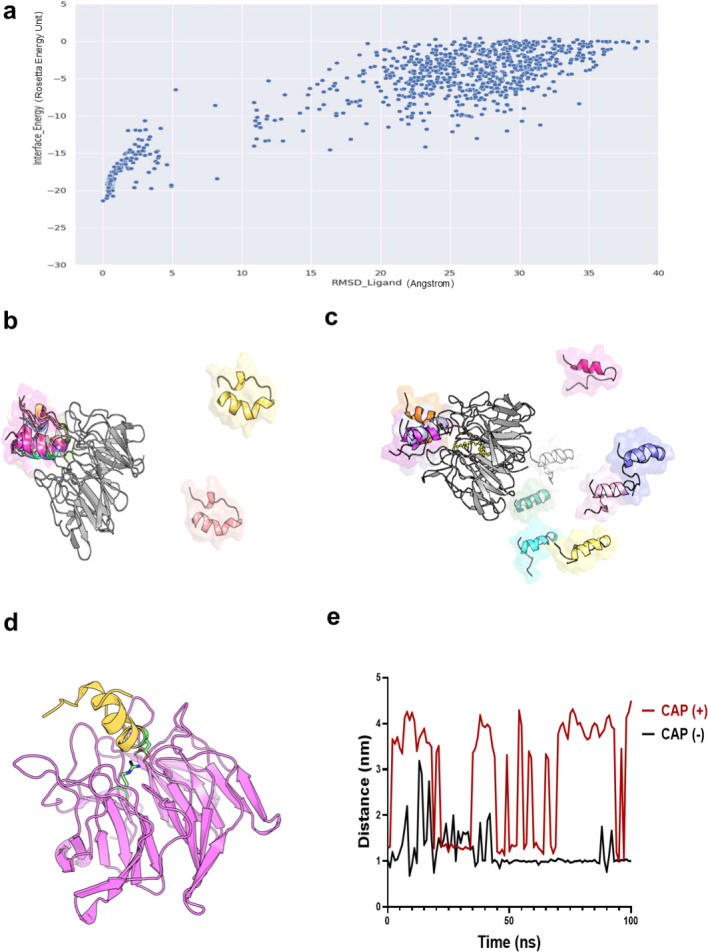
Molecular dynamics simulations of capsaicin (CAP) and Kelch. (**a**) The binding energy landscapes of the molecular docking of CAP and KEAP1. (**b**) Nuclear factor erythroid 2–related factor 2 (NRF2) associated with Keap1 in the absence of CAP. Majority of frames throughout the 100 ns trajectory featured NRF2 binding with Keap1. (**c**) The distribution of NRF2 fragments around Keap1 (gray color) in the presence of CAP (represented as yellow ball/stick). The representative conformations of NRF2 throughout the simulations were superimposed on KEAP1. (**d**) The averaged distance between NRF2 Asp29 and Keap1 Arg415 (represented as green sticks) were measured and recorded in the MD simulations. (**e**) The distance between NRF2 Asp29 and KEAP1 Arg415 in the NRF2-KEAP1 complex (black line) and the NRF2-CAP-KEAP1 complex (red line).

**Figure 4—figure supplement 2.**
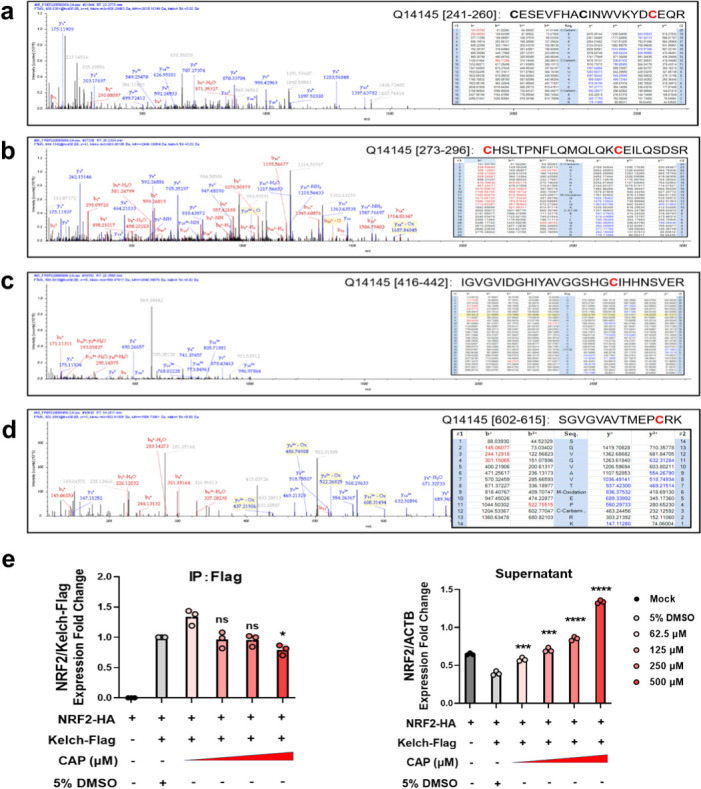
Tandem mass spectrometry (MS/MS) and co-immunoprecipitation (Co-IP) analysis of capsaicin (CAP) and Kelch. (**a**) MS/MS of KEAP1 peptide containing Cys257 after CAP treatment. (**b**) MS/MS of KEAP1 peptide containing Cys273 and Cys288 after CAP treatment. (**c**) MS/MS of KEAP1 peptide containing Cys434 after CAP treatment. (**d**) MS/MS of KEAP1 peptide containing Cys613 after CAP treatment. (**e**) The gray analysis of NRF2 in IP group and in the supernatant of each group.

**Figure 4—video 1.** Molecular dynamics simulations between nuclear factor erythroid 2–related factor 2 (NRF2) and Kelch.

**Figure 4—video 2.** Molecular dynamics simulations between nuclear factor erythroid 2–related factor 2 (NRF2) and Kelch with capsaicin (CAP).

To investigate the specific CAP-binding sites on KEAP1, we utilized computational docking and all-atom simulations to map the positioning of possible CAP-interacting residues within the surface pockets of KEAP1, with a particular focus on the Kelch domain that is responsible for NRF2 interaction (Figure 4b and Figure 4—figure supplement 1a). The most energetically favorable model demonstrated that the vanillyl headgroup of CAP was inserted into the Kelch channel, while its flexible hydrocarbon chain remained at the periphery of the NRF2-Kelch interface. Based on this docking model, we hypothesized that the CAP binding pocket partially overlapped with the KEAP1-NRF2 interacting interface (Figure 4c). Hence, we modeled interactions between NRF2 and Kelch in the presence of CAP using all-atom molecular dynamics simulations. When CAP is associated with Kelch, the NRF2-Kelch complex was destabilized (Figure 4—figure supplement 1b and c and Figure 4—video 1 and Figure 4—video 2). Indeed, for the NRF2-Kelch complex, the average distance between D29 of NRF2 and R415 of Kelch remained approximately 1.20 nm, indicating stable bindings throughout the 100 ns trajectory. For the NRF2-CAP-Kelch complex, however, the average distance fluctuated to 2.87 nm, resulting in the dissociation of NRF2 from the Kelch domain (Figure 4—figure supplement 1d and e). Our simulations support the experimental observation that CAP can act as a protein-protein interface inhibitor.

Interestingly, the majority of previously identified KEAP1 inhibitors were known to induce covalent modifications of the cysteine residues of KEAP1, thereby disrupting the interactions between KEAP1-Cul3 and KEAP1-NRF2, and consequently activating the downstream NRF2-ARE signaling pathway (Lu et al., 2016). Therefore, we introduced the reducing agent dithiothreitol (DTT) into the cell medium. Existing literature suggests that KEAP1’s active cysteines can be covalently altered by electrophilic agents like CN-2F, and such modifications are generally reversible in the presence of DTT (Liu et al., 2022). Additionally, the effectiveness of catechol moieties in NRF2 activation is often diminished by pre-treatment with DTT (Lin et al., 2015). However, our results indicated that the CAP-induced elevation of NRF2 expression levels was not inhibited by DTT (Figure 4d).

To further investigate whether CAP also forms covalent bonds with key cysteine residues on the KEAP1 surface, we targeted our scrutiny towards the functionally critical cysteines, such as Cys151, Cys273, and Cys288 (Saito et al., 2016). Employing tandem mass spectrometry (MS/MS), we assessed the molecular weights of KEAP1 samples following incubation with CAP and found no evidence of CAP-induced modifications (Figure 4e, and Figure 4—figure supplement 2a–2d). These results suggested that although CAP bound directly to KEAP1, it did not do so through covalent interaction.

### CAP specifically interacts with the Kelch domain of KEAP1

To investigate the impact of CAP on the binding affinity between the Kelch domain and NRF2, we conducted immunoprecipitation (IP) and pull-down analysis using purified Kelch-domain-only proteins. The IP results revealed a noticeable reduction in NRF2 proteins bound to the Kelch domain, corresponding with incremental concentrations of CAP. In parallel, the levels of unbound NRF2 in the supernatant progressively increased (Figure 4f and Figure 4—figure supplement 2e). Further substantiating these observations, purified NRF2 proteins were also used. Pull-down assays furnished compelling empirical evidence that CAP effectively disrupted the direct interaction between the Kelch domain and NRF2 (Figure 4g).

### CAP binds KEAP1 at allosteric sites

**Figure 5.**
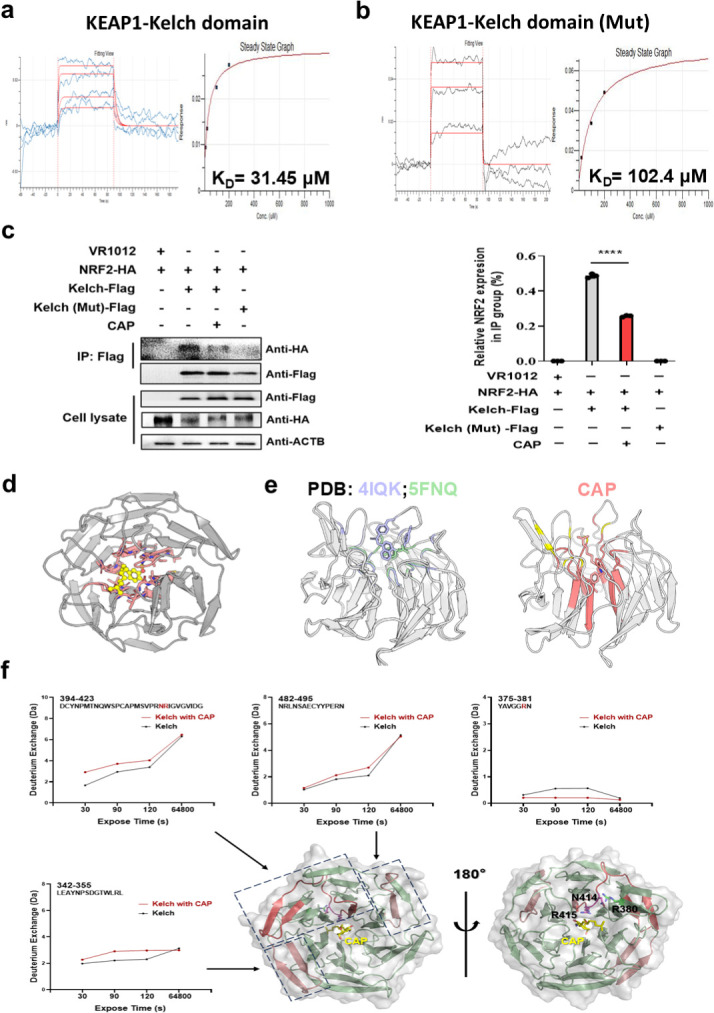
Mutation of KEAP1 affects the function of capsaicin (CAP). (**a**) In vitro detection of KEAP1-Kelch domain with CAP using bio-layer interferometry assay (BLI). (**b**) In vitro detection of KEAP1- Mutant Kelch domain (Y334A, R380A, N382A, N414A, R415A, Y572A, and S602A) with CAP using BLI. (**c**) Co-IP assay to assess the interaction between mutant Kelch and nuclear factor erythroid 2–related factor 2 (NRF2), and the impact of 32 μM CAP on Kelch-NRF2 binding in 293T cells. (**d**) Docking analysis reveals encirclement of CAP’s vanillyl headgroup by main chain atoms of the Kelch domain. (**e**) Left side: existing binding sites of two common ligands with Kelch (PDB:4IQK and 5FNQ); right side: newly discovered binding sites of CAP with Kelch. (**f**) CAP allosterically regulated the conformation of Kelch by hydrogen-deuterium exchange mass spectrometry (HDX-MS). Peptides with increased deuterium uptake ratio after CAP treatment are highlighted in red. Figure 5—source data 1.TIFF files that contain original western blots indicating the relevant bands and treatments. Figure 5—source data 2.Original files for western blot analysis.

According to the results of molecular docking and dynamic simulation, we purified both the wild-type Kelch domain (WT) and the mutant variant (Mut) of Kelch proteins. The mutant variant involved substitutions at residues Y334A, R380A, N382A, N414A, R415A, Y572A, and S602A (the orthostatic site), which are residues reported to engage NRF2 and traditional Keap1 inhibitors. Bio-Layer Interferometry (BLI) revealed that the binding affinity between CAP and the Kelch domain (WT) was 31.45 μM with R^2^=0.99 (Figure 5a). Intriguingly, the mutated Kelch domain (Mut) exhibited diminished, but still appreciable, affinity for CAP with a K_D_ of 102.4 μM with R^2^=0.98 (Figure 5b). Our biophysical examinations suggested the orthostatic NRF2-binding pocket was not entirely responsible for CAP-binding. Indeed, the Kelch (Mut)-Flag protein failed to pull down any NRF2 from cellular lysates, but it still interacted with CAP (Figure 5c). These collective findings suggest that the CAP-binding pocket and the NRF2-binding interface on KEAP1 may be different from each other, implicating CAP as an allosteric modulator of KEAP1. This specificity may arise from the interaction between the vanillyl headgroup of CAP and the main chain atoms of the Kelch domain, which appears to be largely unaffected by NRF2 binding (Figure 5d). The crystal structures depicting the binding sites between previously identified ligands and the Kelch protein (PDB: 4IQK and 5FNQ) were illustrated on the left-hand side of Figure 5e. In contrast, the right-hand side revealed our discovery of the binding sites between CAP and the Kelch protein, which were very inconsistent with previous reports. Here, to explore the potential mechanism of allosteric regulation of the Kelch domain with CAP, we performed HDX-MS assays. The addition of CAP led to an increase in the hydrogen-deuterium exchange rate in about three regions of Keap1, L342-L355, D394-G423, and N482-N495, suggesting their conformational changes. It was noteworthy that amino acid residues N414 and R415 are located in the D394-G423 region, which were the key residues for the interaction with NRF2, and CAP binding may lead to their changes, thus CAP may have weakened the interaction between KEAP1 and NRF2 by way of allosteric regulation (Figure 5f). These insights suggested that CAP could potentially represent a novel class of NRF2 agonists.

### Preparation and characterization of IR-HSA@CAP

**Figure 6.**
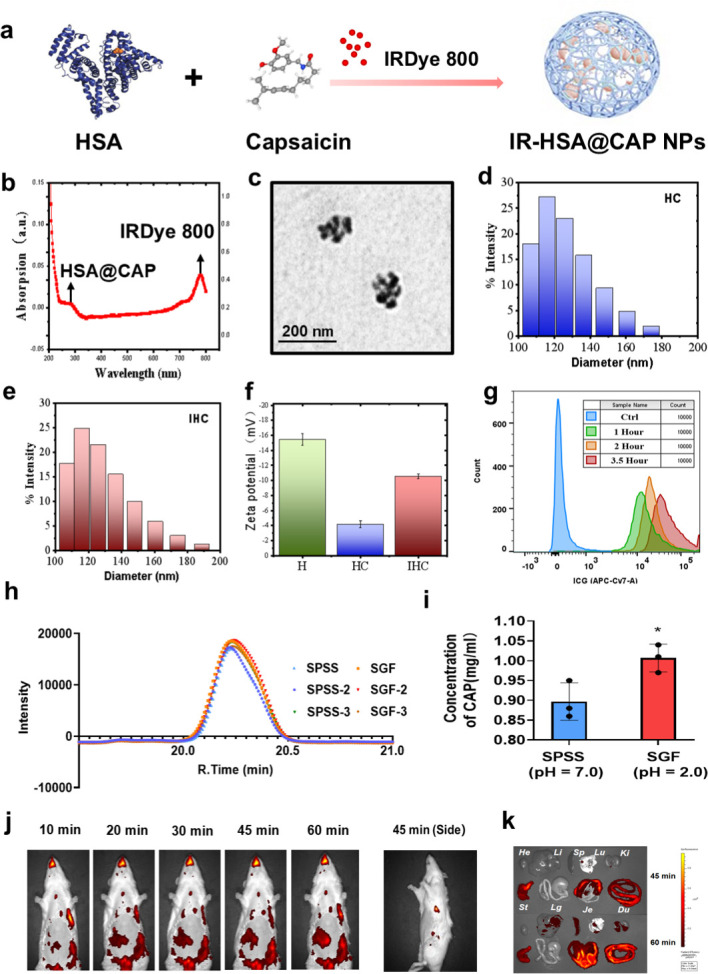
Preparation and characterization of IR-HSA@CAP NPs. (**a**) Schematic illustration of the synthesis process for IR-HSA@CAP nanoparticles (NPs). (**b**) Determination of IRDye800 binding with human serum albumin (HSA) by UV-Vis spectra. (**c**) The morphology of IR-HSA@CAP NPs was investigated by transmission electron microscope. Scale bar, 0.5 μm. (**d-e**) Particle size comparison of HSA@CAP and IR-HSA@CAP NPs. (**f**) Zeta-Potential analysis of nanomaterials. The zeta-potential values of the different materials were measured: H representing HSA, HC representing HSA@CAP NPs, and IHC representing IR-HSA@CAP NPs. (**g**) Flow cytometric (FCM) analysis demonstrates endocytosis of IR-HSA@CAP NPs by GES-1 cells. (**h–i**) Detection of CAP release in simulated gastric fluid (SGF) and stroke-physiological saline solution (SPSS) using high-performance liquid chromatography (HPLC). (**j**) In vivo imaging reveals the localization of IR-HSA@CAP NPs. (**k**) Observing the distribution of IR-HSA@CAP NPs in major organs of rats.

Encouraged by the discovery of CAP as a novel agonist of NRF2, we developed a convenient nano-drug delivery platform for the efficient utilization of CAP in vivo. We opted for Human Serum Albumin (HSA) as the encapsulating material, given its FDA-approved status for biocompatibility. HSA has also been documented to offer protective effects on the gastric mucosa (Wu et al., 2021). As a fluorescent marker, we employed IRDye800, a widely utilized near-infrared fluorescent dye in biomedical research for applications, such as protein tagging and imaging applications. Utilizing these components, we successfully formulated CAP-encapsulated IRDye800-HSA nanoparticles, referred to as IR-HSA@CAP (Figure 6a). The labeling ratio stood at 1.48 (IRDye800: HSA) and achieved a coupling efficiency of 93.29% (Figure 6b), and the encapsulation was notably high as calculated (Bhattacharyya et al., 2014).

**Figure 6—figure supplement 1.**
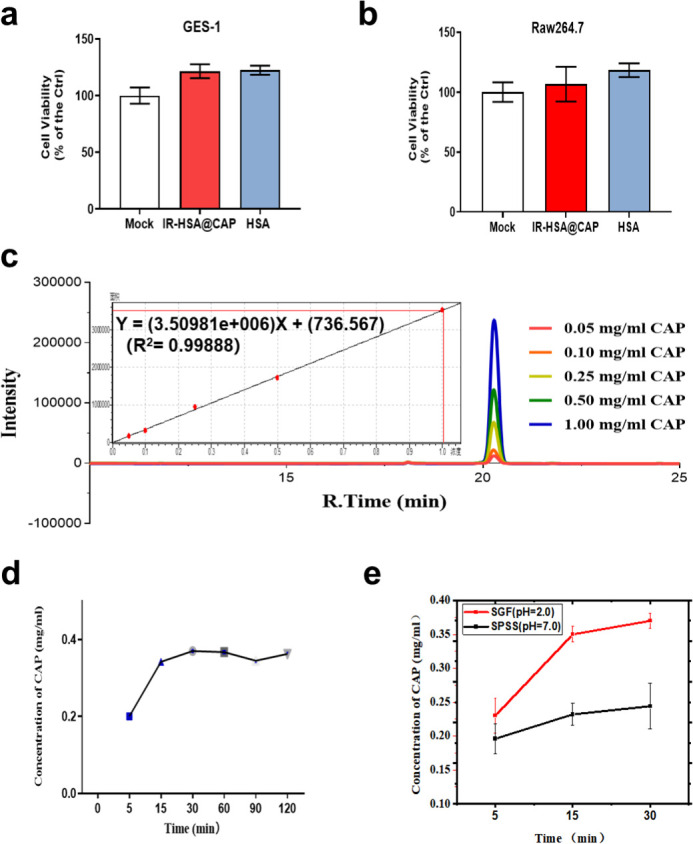
Biosafety and concentration determination of IR-HSA@CAP. (**a and b**) IR-HSA@CAP NPs including human serum albumin (HSA) (0.04 mg/ml) and CAP (0.02 mg/ml) were added into GES-1 and Raw264.7 cells for 24 hr to assess biosafety. (**c**) Standard curve for determination of capsaicin (CAP) by high performance liquid chromatography (HPLC). (**d**) The concentration of CAP released from IR-HSA@CAP NPs (including 0.4 mg/ml CAP) after a certain period of treatment in simulated gastric fluid (SGF) by HPLC. (**e**) The concentration of CAP released from IR-HSP@CAP NPs (including 0.4 mg/ml CAP) was measured in SGF and stroke-physiological saline solution (SPSS) for 30 min, respectively.

The structural attributes of the IR-HSA@CAP nanoparticles were characterized using transmission electron microscopy (TEM), the results of which were illustrated in Figure 6c. Notably, the size distributions of HSA@CAP and IR-HSA@CAP nanoparticles were remarkably similar. The former exhibited a mean particle size of 139.27±7.49 nm (Figure 6d), while the latter registered at approximately 160.97±14.78 nm (Figure 6e). In terms of zeta-potential, the HSA@CAP nanoparticles displayed an increased to –4.16±0.78 mV post-formation, as compared to native HSA, which had a potential of –15.45±1.40 mV. Interestingly, this zeta-potential returned to a value of –10.55±0.50 mV after incorporating IRDye800 in the IR-HSA@CAP (Figure 6f).

Importantly, no discernible decline in cell viability was observed over a 24 hr period in both GES-1 and RAW264.7, underscoring the non-cytotoxicity of the nanoparticles (Figure 6—figure supplement 1a and b). Furthermore, intracellular fluorescence intensity escalated continuously with time, reaching a point where nearly all cells exhibited IR-Dye800 positivity within 1 hr (Figure 6g). These findings strongly implied that the IR-HSA@CAP nanoparticles were rapidly internalized by cells and show excellent biocompatibility.

To precisely quantify the CAP content in IR-HSA@CAP nanoparticles, we established a standard curve for CAP using high-performance liquid chromatography (HPLC) at a wavelength of 282 nm (Figure 6—figure supplement 1c). To evaluate the stability and release profile of the nanoparticles in gastrointestinal conditions, we examined the release kinetics of CAP in normal saline (SPSS, pH = 7.0) and simulated gastric fluid (SGF, pH = 2.0). Initial tests revealed that virtually all encapsulated CAP could be rapidly released in the SGF (Figure 6—figure supplement 1d and e). In subsequent animal trials calibrated to the actual concentrations of CAP, we found that the CAP concentration in SGF swiftly attained a theoretical value of 1 mg/ml within 30 min, while remaining essentially constant in SPSS conditions (Figure 6h and i). These findings collectively suggest that IR-HSA@CAP nanoparticles were capable of achieving rapid release of CAP in the gastric milieu to a notable extent.

### IR-HSA@CAP NPs activated the Nrf2-ARE signaling pathway in vivo

To investigate the protective effects of IR-HSA@CAP nanoparticles against EtOH-induced gastric mucosal injury in vivo, Sprague-Dawley rats were randomly assigned to eight different experimental groups. Rebamipide was selected as the positive control due to its well-established ability to protect gastric mucosa (Moon et al., 2014).

The in vivo experimental protocol was conducted as follows: To ensure gastric emptying, rats were subjected to a 24 hr fasting period. Subsequently, they were administered SPSS, CAP, or the designated treatment in accordance with their group allocation. After a 45 min pretreatment phase, the rats were exposed to 75% EtOH for an additional 1.5 hr. Following this, the animals were humanely euthanized, and the gastric tissue samples were collected for subsequent experiments and analyses. Precise dosages administered were as follows: CAP at 1 mg/kg, HSA at 2 mg/kg, and Rebamipide at 100 mg/kg. The HSA@CAP nanoparticles used were prepared to contain 1 mg/kg of CAP and 2 mg/kg of HSA, as quantified by HPLC three times.

To elucidate the spatiotemporal distribution of CAP within the rat model, imaging was performed at various time intervals following administration. The data revealed that CAP was predominantly absorbed by the gastrointestinal tract, exhibiting significant localization in the stomach (Figure 6j and k).

**Figure 7.**
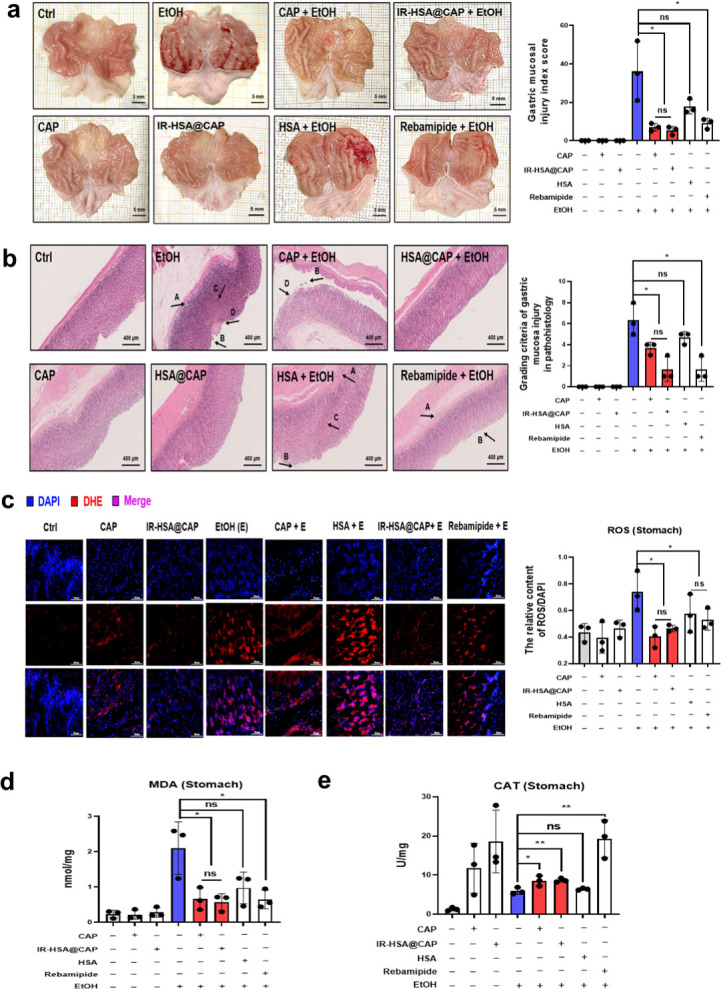
Capsaicin (CAP) activated nuclear factor erythroid 2–related factor 2–antioxidant response element (NRF2-ARE) pathway in vivo. (**a**) Impact of CAP (1 mg/kg) on histopathology of ethanol (EtOH)-induced gastric mucosal injury. Rebamipide (100 mg/kg) was used as a positive control. Tissue sections were stained and evaluated for gastric mucosal ulcer injury using the Guth scoring system. Representative images were shown with a scale bar of 5 mm. The ulcer injury (UI) index was calculated. (**b**) Histological examination of rat gastric mucosa using hematoxylin and eosin (H&E) staining. Scale bar represents 400 μm. A: Inflammatory cell infiltration; B: Epithelial exfoliation; C: Glandular disorder; D: Gastric edema. Quantitative analysis was conducted using the Masuda scoring system. (**c**) Visualization of reactive oxygen species (ROS) in rat gastric tissue under various treatments using dihydroethidium (DHE) staining and inverted fluorescence microscopy. Scale bar represents 50 μm. ROS levels were quantified using Image Pro Plus 6.0 software. (**d**) MDA levels in gastric tissues across different treatment groups. (**e**) Assessment of catalase (CAT) activity in gastric tissues.

The administration of either CAP or IR-HSA@CAP alone did not result in detectable damage to the gastric tissues, in contrast to the control group. However, significant hemorrhaging and erosion were evident in gastric tissues exposed to EtOH (Figure 7a, left). Utilizing the Guth scoring system, we quantitatively assessed the Gastric Mucosal Ulcer Injury (UI) index. The average UI index in the EtOH group increased to 36.0, indicating acute tissue damage. On the other hand, significant reductions in the UI index were observed in both the CAP and IR-HSA@CAP pretreatment groups, registering at 7.0 and 5.3, respectively. These levels were similar to the Rebamipide group, which had a UI index of 9.0 (Figure 7a, right). These findings align well with our expectations. Notably, the effective dose of CAP (1 mg/kg) was substantially lower than that of Rebamipide (100 mg/kg), underscoring the potential advantages of CAP. Additionally, while the HSA-pretreated group did exhibit some attenuation of gastric mucosal injury, the reduction was not statistically significant (UI = 17.7). This suggested that CAP was the principal active component responsible for the observed effects in the IR-HSA@CAP nanoparticles.

Subsequently, we quantified the extent of pathological damage using H&E staining coupled with the Masuda scoring system, wherein a higher index reflects more severe tissue damage. The pathological damage index registered at 6.3 in the EtOH-only group, while the HSA-treated group showed a slightly lower index at 4.7. The CAP-treated group further reduced this index to 3.7. Remarkably, the employment of either IR-HSA@CAP nanoparticles or Rebamipide led to a substantial decline in pathological damage, with both treatments registering an identical index of 1.7 (Figure 7b). These results demonstrate that the therapeutic efficacy of IR-HSA@CAP nanoparticles surpassed that of either CAP or HSA administered individually. Specifically, the reduction in tissue damage was 1.77-fold greater than that achieved with CAP alone and 2.88-fold greater compared to HSA alone.

To evaluate the capability of CAP to inhibit the EtOH-induced augmentation of ROS levels in gastric tissues in vivo, we conducted dihydroethidium (DHE) staining on cryo-sectioned gastric specimens. Pre-treatment with either CAP or IR-HSA@CAP nanoparticles substantially mitigated ROS production, with reductions of 45.42% and 36.99%, respectively, compared to the EtOH-only group (Figure 7c). Concurrently, MDA assays underscored that IR-HSA@CAP nanoparticles efficaciously curtailed lipid peroxidation levels in the gastric tissues by an approximate 72.82% (Figure 7d). Additionally, CAT activity in the tissues was enhanced by 31.85% following treatment with IR-HSA@CAP nanoparticles (Figure 7e).

**Figure 8.**
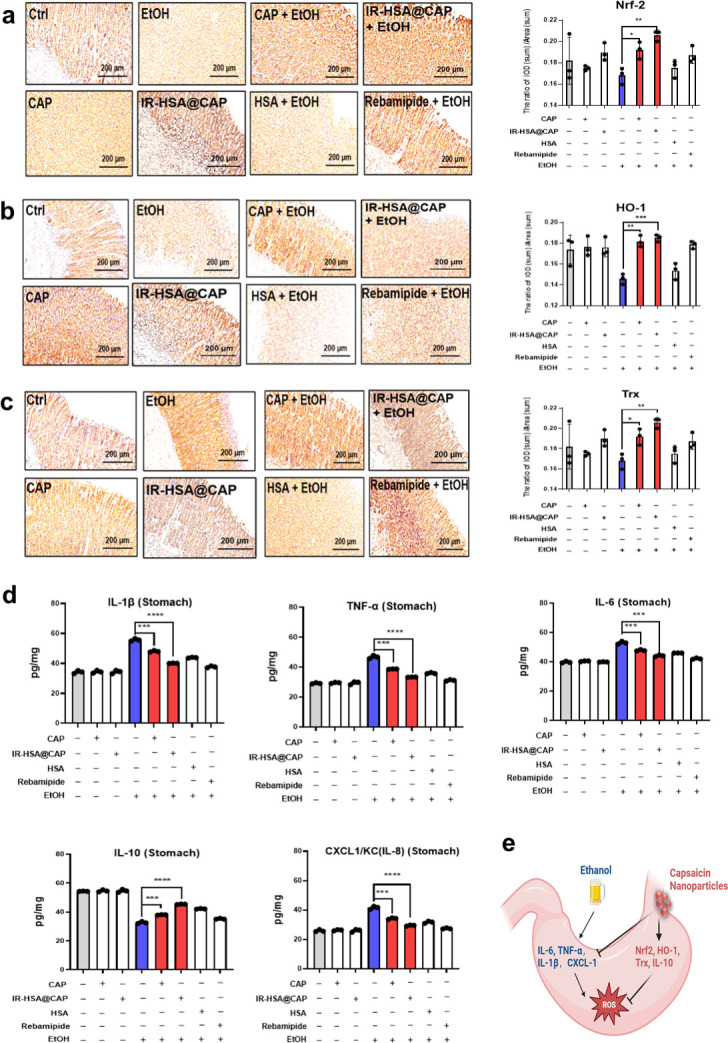
Capsaicin (CAP) also suppressed inflammation in vivo. (**a–c**) Immunohistochemical detection of antioxidant proteins and presentation of representative images and statistical analysis results. (**a**) Nuclear factor erythroid 2–related factor 2 (Nrf2), (**b**) heme oxygenase 1 (HO-1), and (**c**) thioredoxin (Trx) protein expression levels were assessed. (**d**) ELISA measurement of IL-1β, TNF-α, IL-6, CXCL1/KC (IL-8), and IL-10 levels in gastric tissues. Data were presented as mean ± SD. Significance levels were indicated as follows: ns, not significant; *p<0.05; **p<0.01; ***p<0.001; ****p<0.0001 compared to the ethanol (EtOH)-only group (marked in blue). (**e**) Mechanism diagram of CAP alleviating gastric mucosal injury caused by ethanol in rats.

Furthermore, we scrutinized the modulatory effects of IR-HSA@CAP on the Nrf2-ARE signaling pathway. Our findings, substantiated through immunohistochemistry (Figure 8a–c) and western blot analyses (Figure 8—figure supplement 1j–1m), revealed that pre-treatment with IR-HSA@CAP led to a significant upregulation of Nrf2 expression. Concurrently, IR-HSA@CAP pre-treatment also stimulated elevated expression levels of HO-1 and Trx. These collective observations suggest that IR-HSA@CAP was capable of orchestrating the Nrf2-ARE signaling axis, thereby providing a rapid and effective modulatory mechanism for oxidative stress and homeostatic regulation. To delve deeper into the anti-inflammatory potential of our treatment strategy, we analyzed homogenized gastric tissue samples for the expression profiles of various chemokines and inflammatory mediators. Intriguingly, pre-treatment with either CAP or IR-HSA@CAP elicited a marked suppression in the expression levels of pro-inflammatory cytokines, such as IL-1β, TNF-α, IL-6, and CXCL1, while simultaneously augmenting the expression of the anti-inflammatory cytokine IL-10 (Figure 8d). Not surprisingly, IR-HSA@CAP exhibited a stronger anti-inflammatory effect. The novel mechanism of CAP nanoparticles in preventing EtOH-induced gastric mucosal injury is shown in Figure 8e.

**Figure 8—figure supplement 1.**
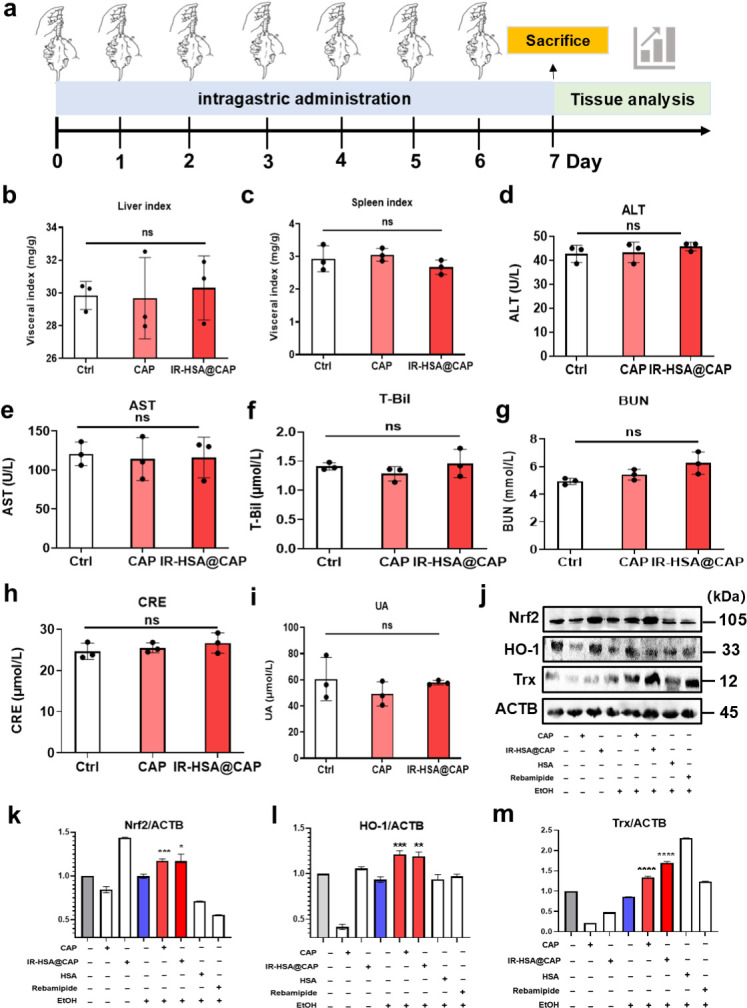
Safety evaluation of animal experiments. (**a**) A model diagram of safety trial about capsaicin (CAP) and IR-HSA@CAP NPs in rats. (**b and c**) Organ index of liver and spleen. ns, not significant. Data were presented as mean  ± SD, p-values were calculated using t-test. ns, not significant. (**d-f**) Determination of ALT, AST, and T-Bil in serum to evaluate liver function. (**g–i**) Determination of BUN, CRE, and UA in serum to evaluate renal function. Data were presented as mean  ± SD, p-values were calculated using t-test. ns, not significant. (**j–m**) Western blot analysis of antioxidant protein expression of nuclear factor erythroid 2–related factor 2 (NRF2), heme oxygenase 1 (HO-1), and thioredoxin (Trx). Figure 8—figure supplement 1—source data 1.TIFF files that contain original western blots indicating the relevant bands and treatments. Figure 8—figure supplement 1—source data 2.Original files for western blot analysis.

Finally, we assessed the biosafety of IR-HSA@CAP in rats through continuous oral administration over a one-week period (Figure 8—figure supplement 1a). No significant alterations were observed in the organ indices for either the liver or spleen (Figure 8—figure supplement 1b and c). Comprehensive blood biochemical analyses further revealed that varying concentrations of IR-HSA@CAP exerted no deleterious impact on hepatic function, as indicated by levels of ALT, AST, and T-Bil (Figure 8—figure supplement 1d–1f), or on renal function, as evidenced by measurements of BUN, CRE, and UA (Figure 8—figure supplement 1g–1i). These collective findings underscore the high biosafety profile of the IR-HSA@CAP nanoparticles.

### Protective effect of IR-HSA@CAP NPs was partially eliminated in Nrf2-KO mice

A direct proof that IR-HSA@CAP NPs act through activation of Nrf2 in protecting gastric mucosa against alcohol toxicity could be well conducted in commercially available Nrf2-knockout (KO) mice. Therefore, we conducted experiments in normal C57BL/6 mice and Nrf2-knockout mice.

**Figure 9.**
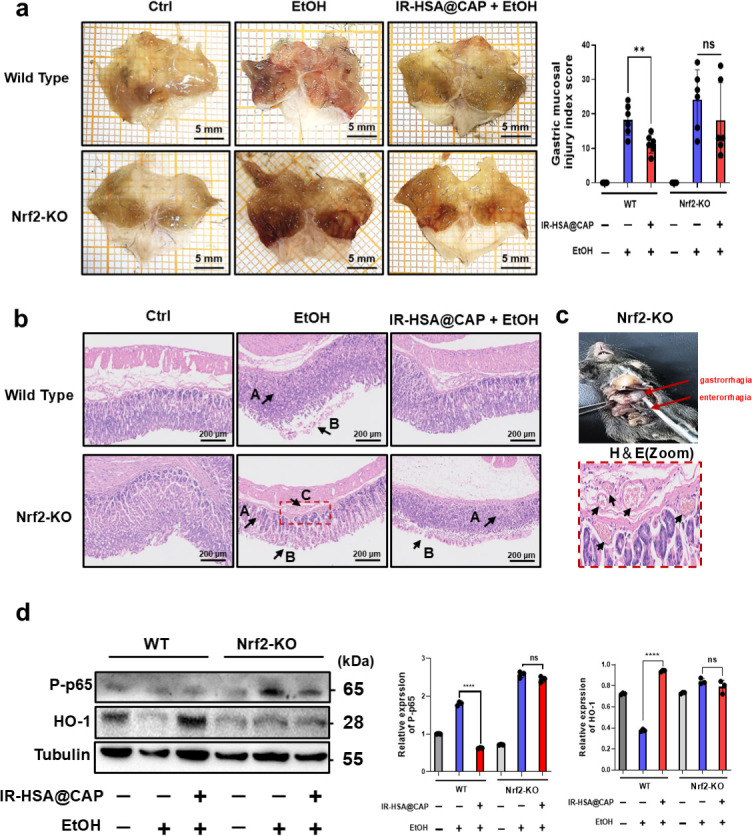
Protective effect of capsaicin (CAP) was partially eliminated in nuclear factor erythroid 2–related factor 2 -knockout (Nrf2-KO) mice. (**a**) Tissue sections were evaluated for gastric mucosal ulcer injury using the Guth scoring system. Representative images were shown with a scale bar of 5 mm. The ulcer injury (UI) index was calculated. (**b**) Histological examination of mice gastric mucosa using hematoxylin and eosin (H&E) staining. Scale bar represents 200 μm. A: Glandular disorder; B: Epithelial exfoliation; C: Inflammatory cell and erythrocyte infiltration. (**c**) Gastrointestinal bleeding and H&E staining (Zoom) in a representative Nrf2-KO mouse treated with ethanol (EtOH). (**d**) Western blotting to detect the key proteins of P-p65 and heme oxygenase 1 (HO-1). Figure 9—source data 1.TIFF files that contain original western blots indicating the relevant bands and treatments. Figure 9—source data 2.Original files for western blot analysis.

The phenotype and H&E staining were shown in Figure 9a and Figure 9b. In normal mice, EtOH can still cause significant gastric mucosal damage, and our drug also had a beneficial effect on mice. In the Nrf2-KO group, we found that these mice showed more sensitivity to EtOH. The gastric injury of model group without Nrf2 expression was more obvious than normal mice given the same amount of EtOH, because we found significant gastrointestinal bleeding (Figure 9c and a representative photograph). This suggested that Nrf2 may be important for mice to cope with gastric mucosal damage caused by EtOH.

At the same time, the gastric injury after drug treatment was only partially reduced in Nrf2-KO mice, indicating that the IR-HSA@CAP NPs played a key role by activating Nrf2 in vivo. Here, we also selected two proteins related to inflammation (phosphorylated p65) and antioxidant (HO-1, one of the most critical downstream genes of NRF2) for further western blotting experiments. The results showed that CAP could play an antioxidant and anti-inflammatory role in normal mice, while the related proteins in knockout mice were not significantly different from those in the model group (Figure 9d). Additional experiments were conducted on rats, as shown in Figure 7 and Figure 8. These results were consistent with our expectations that IR-HSA@CAP NPs act through activation of Nrf2 in protecting gastric mucosa against alcohol toxicity.

## Discussion

CAP, a natural small molecule derived from chili peppers, has been identified and verified to modulate the KEAP1-NRF2-ARE pathway in our study. This signaling pathway plays a key role in several cancerous and non-cancerous diseases (Tossetta et al., 2023b; Xia et al., 2023; Tossetta et al., 2023c; Tossetta et al., 2023a; Bukke et al., 2022). Therefore, it is a promising therapeutic target for reducing free radical damage associated with numerous conditions, including Alzheimer’s disease (Osama et al., 2020), chronic kidney disease (Guerrero-Hue et al., 2020), cardiovascular disease (Reuland et al., 2013), cancer, and aging (Schmidlin et al., 2019).

Over the past few decades, the scientific community has tirelessly sought molecules capable of activating NRF2. Hu et al*.* reported the first small-molecule inhibitor of the Keap1-Nrf2 PPI from a high-throughput screen (HTS) in 2013 (Hu et al., 2013). Davies and co-workers began with a crystallographic screen of approximately 330 fragments and developed KI-696 as a nanomolar KEAP1-NRF2 PPI inhibitor (Davies et al., 2016; Heightman et al., 2019). A spectrum of NRF2 agonists, both natural and synthetic, covalent, and non-covalent, have been unveiled. However, Kim T. Tran et al. reported that many compounds are difficult to synthesize and the binding activity of half of the compounds cannot be demonstrated convincingly (Tran et al., 2019). Practical application faces two primary challenges. First and foremost, covalent modifications to KEAP1 pose a noteworthy concern regarding ‘off-target’ effects owing to their electrophilic properties. These effects have the potential to impact various other cellular proteins, impeding their commercial success (Unni et al., 2021). Furthermore, unregulated activation of NRF2 can inadvertently foster cancer progression by protecting malignant cells and promoting their proliferation, metastatic potential, and resistance to therapeutic interventions (Sivinski et al., 2021).

Recent advancements have been geared towards the development of direct inhibitors for the KEAP1-NRF2 interaction, drawing significant attention from the scientific community (Abed et al., 2015). Interestingly, our research unequivocally identifies the non-covalent interaction between CAP and KEAP1. Dimethyl fumarate (DMF), a KEAP1-dependent NRF2 agonist, has progressed positively in phase III clinical trials. The recognition that DMF’s minimal side effects stem from its dual mode of action—both covalently modifying reactive cysteines and non-covalently activating NRF2 via the Kelch domain of KEAP1—has marked a pivotal milestone in the development of drugs targeting KEAP1 (Unni et al., 2021). We found that at lower concentrations, CAP effectively bound with the Kelch domain (Figure 5a and c) and activated NRF2, subsequently promoting the expression of downstream antioxidant genes (Figure 1i–m). In vivo studies, we observed almost no adverse reactions. Additionally, Merritt, J.C. et al. reported that CAP at high concentrations possesses potent anti-cancer properties (Merritt et al., 2022). This aligns with our CCK8 experimental findings that heightened CAP concentrations curtailed cell viability (Figure S1cd). The duality of pharmacological impact of CAP remains a topic of discussion. The interplay and mechanisms between CAP’s varying modes in both regular and cancerous cells, which underlie its antioxidant and anticancer actions, are intricate and warrant further exploration.

Notably, to date, no natural product has been confirmed as a direct inhibitor of the KEAP1-NRF2 interaction. In our findings, the dissociation constant (K_D_) between CAP and Kelch was reported as 31.45 μM (Figure 5a), a relatively weak association compared with ML334 (K_D_ = 1 μM) and some synthetically designed peptides (Crisman et al., 2023). However, the KEAP1-NRF2 interaction was significantly inhibited by CAP (Figure 3d and e). Of particular significance and surprise, our investigation revealed that CAP and Kelch establish interactions at allosteric sites. Furthermore, HDX-MS was first used to identify the potential allosteric regulatory site of KEAP1 by natural small molecule. In particular, the deuterium exchange of the new peptide (D394-G423) we found was significantly increased, and it was also observed in a newly screened natural small molecule and Kelch (data not shown). Our comprehensive analysis of the CAP-KEAP1 binding dynamics, particularly the unique mode of interaction with Kelch, offers valuable insights for the development of innovative NRF2 activators.

Nonetheless, several concerns warrant further examination. The impact of CAP on the gastric mucosa, as evidenced in animal studies, is contingent upon its dosage and duration of administration. A low dose of CAP applied short-term can mitigate indomethacin-induced gastric microhemorrhage. Conversely, a high dose applied prophylactically over an extended period does not confer this benefit (Luo et al., 2011; Peng and Li, 2010; Baylie and Brayden, 2011). It was similarly observed in our studies, where the effective concentration window for natural CAP is a somewhat narrow. For individuals with pre-existing gastric conditions, the epithelial cells of the gastric mucosa might exhibit heightened sensitivity to CAP. This not only diminishes its protective capability but might also exacerbate ulcer severity. Concurrently, the IR-HSA@CAP NPs we employed demonstrated prompt gastric release in vivo, underscoring the superior attributes of nanomedicine. This could amplify the antioxidant and anti-inflammatory potency of CAP, thereby resulting in enhanced therapeutic outcomes. Yet challenges persist, particularly the inability to achieve a treatment that targets the stomach exclusively. A limitation of this study is the use of only three rats per group for the in vivo experiments. Considering ongoing research into nanomaterials for CAP, there is an opportunity to further refine CAP’s delivery strategy from a pharmacological standpoint, and in future related studies, we will strive to increase the sample size to enhance the robustness of our findings.

In summary, the present study provides evidence that CAP effectively mitigates oxidative stress through the activation of NRF2 and its non-covalent binding to KEAP1’s Kelch domain. The unique binding site between CAP and Kelch distinguishes from the commonly reported small molecule inhibitors, hinting at its potential as a novel class of NRF2 agonists. Moving forward, the development of derivatives inspired by CAP holds promise as a compelling avenue for the creation of therapeutic agents aimed at attenuating oxidative damage.

## Materials and methods

### Reagents and chemicals

CAP used in vitro was purchased from Solarbio Life Science (CAS:404-86-4, Cat No. IC0060). CAP used in vivo was purchased from MCE (Cat No. HY-10448). The anhydrous ethanol (EtOH) was purchased from Tianjin Jiangtian Chemical Technology Co. LTD in China (CAS:64-17-5, Cat No.268). Primary antibodies of anti-HA-Tag (Cat No. AE008), anti-Strep II-Tag (Cat No. AE066), and anti-MYC-Tag (Cat No. AE010) were purchased from ABclonal Technology. The primary antibody to anti-Flag (Cat No. 80010–1-RR), anti-Actin (catalog no. 66009–1-Ig), anti-GAPDH (Cat No. 60004–1-Ig), anti-NRF2 (Cat No. 16396–1-AP), and anti-NRF2 in revised version (Cell Signaling Technology, Cat. No. 12721), anti-GSS (Cat No. 15712–1-AP), anti-HMOX1(Cat No. 10701–1-AP) anti-TXN (Cat No. 14999–1-AP), and anti-KEAP1(Cat No. 30041–1-AP) were purchased from Proteintech in China. Anti-HA affinity matrix (Cat No. 11815016001) was purchased from Roche. Anti-Flag affinity matrix (catalog no. A2220) and albumin from human serum (HSA, Cat No. A3782) was purchased from Sigma. The FITC Goat anti-Rabbit IgG (H+L) was purchased from SIMUBIOTECH (Cat No. S2003). Reactive Oxygen Species Assay Kit (Cat No. CA1410), and the CCK-8 cell proliferation and cytotoxicity assay kit (Cat No. CA1210) were acquired from Solarbio Life Science. The enhanced chemiluminescence (ECL) reagent (Cat No. SQ201) was obtained from EpiZyme. Cycloheximide (CHX, Cat No. HY-12320), DTT (Cat No. HY-15917), and Bortezomib (PS-341, Cat No. HY-10227) were purchased from MCE. Human KEAP-1 Protein was bought from Sino Biological (Cat No.11981-HNCB). Recombinant human NRF2 protein was bought from Abcam (Cat No. ab132356). IRDye 800CW was bought from *LI-COR*. Dimethyl sulfoxide (DMSO) was bought from Sigma (Cat No. D8418) for use as a drug solvent and control. Fetal Bovine Serum (FBS) was bought from Gibco (Cat No. 10099–141) as one of the components of the culture medium. Lipofectamine 2000 was bought from Thermo Fisher (Cat No. 11668027) for plasmid transfection.

### Strain, strain background

Sprague Dawley Rats (CD IGS) Rats (SD rats, Cat No.101) were bought from Beijing Vital River Laboratory Animal Technology Co., Ltd. C57BL/6 (Cat No. SM-001) and Nrf2-knockout (Nrf2-KO) mice (Cat No. NM-KO-190433) on C57BL/6 J background were purchased from Shanghai Model Organisms Center, Inc.

### Cell lines and cell culture

Human GES-1 were bought from Procell Life Science & Technology Co., Ltd, HEK293T human embryonic kidney 293 cells (293T), and Human umbilical cord mesenchymal stem cells (UC-MSC) were obtained from the American Type Culture Collection (ATCC) and cultured at 37 °C in 5% CO_2_. KEAP1 Knockout 293T Cell lines (293T-KO) were purchased from ABclonal (RM02350). All cell lines were authenticated by short tandem repeat (STR) profiling and tested negative for mycoplasma contamination. 293T and 293T-KO were maintained in Dulbecco’s modified Eagle’s medium (DMEM) supplemented with 10% fetal bovine serum (FBS) and 1% penicillin-streptomycin (100 IU/mL). GES-1 and UC-MSC were maintained in Roswell Park Memorial Institute 1640 medium (RPMI-1640), supplemented with 10% fetal bovine serum (FBS) and 1% penicillin-streptomycin (100 IU/mL), RAW 264.7 also needs an additional 1% Glutamax. The cell passage ratio was 1:2-1:4, every 2–3 days.

### Plasmid construction

The plasmids of pCDNA3.1-KEAP1-FLAG (Cat No. P8893) and pCMV-NRF2 (human)-HA-Neo (Cat No. P45110) were purchased from the platform of MiaoLingPlasmid (http://www.miaolingbio.com/). In order to better express NRF2 in 293T, we inserted the NRF2 (human)-HA into EcoRV/XbaI of VR1012 through the pEASY-Basic Seamless Cloning and Assembly Kit purchased from Transgen Biotech (Cat No. CU201-02). The plasmid of Ub-K48-Myc and the no-load plasmids of pcDNA3.1 and VR1012 are available in our own laboratory. The Kelch domain of KEAP1(Lys312-Cys624) (Begnini et al., 2022) was downloaded and then the optimized (*for Escherichia coli; Homo sapiens*) sequence was cloned into the vector of pET-28a (+) by adding 5' (NdeI) and 3' (XhoI), so the plasmid of Kelch in pET-28a (+) was constructed from the company of Azenta. At the same time, a Kelch plasmid with mutation at 7 sites (R415A, N382A, R380A, N414A, S602A, Y334A, and Y572A) was designed, which was also synthesized directly using the same method described above.

### CCK-8 cell viability and cytotoxicity assay

GES-1 cells cultured in 96-well plates were used for CCK8 experiments. After the cells had been cultured for more than 24 hr and spread all over the bottom of the plate (10^4^ cells/ml), the experiments were carried out. The cells were washed twice with PBS, low, medium, and high concentrations of ethanol (EtOH) were added into the medium of 1640 (0.5%, 3.5% and 5% v/v) and cultured for 1.5 hr at 37 °C in 5% CO_2_. After the 1.5 hr, carefully discard the supernatant before slowly adding the well-diluted CCK8 solution, and the absorbance at 450 nm was measured using a microplate reader according to the manufacturer’s instructions. To identify the effective concentration of CAP, CAP dissolved in DMSO (original concentration of 50 mM) was diluted to the specified concentration in 1640 medium (2, 4, 8, 16, and 32 μM), mixed well and added to the cells for 2.5 hr. and then added the corresponding concentration of EtOH directly for another 1.5 hr. After a total of 4 hr of treatment, CCK8 cell viability was measured according to the above method. UC-MSC cells was treated like GES-1, using 200 μM H_2_O_2_ to replace EtOH. What’s more, in order to judge the safety of nanomaterials, HSA@CAP NPs, including HSA (0.04 mg/ml) and CAP (0.02 mg/ml) were added into GES-1 and RAW264.7 cells for 24 hr and then the cytotoxicity assay was conducted the same as the tests for cell viability.

### Effect of CAP and EtOH on the morphology of GES-1

GES-1 cells with good condition were cultured in 24-well plates at a concentration of 5×10^5^/well. Then, 5% EtOH was added in the EtOH group and the cells pre-added with 2 μM or 8 μM of CAP were termed as the ‘2 μM CAP+EtOH’ group and the ‘8 μM CAP+EtOH’ group. Cell morphology of each group was observed under the fluorescence microscope using only the bright field. In subsequent cell experiments, we also adopted the same modeling method and set such four groups, hereinafter referred to as the model construction of this experiment.

### Flow cytometry of apoptosis

GES-1 cells cultured in 6-well plates were used for detecting apoptosis. Annexin V-FITC Apoptosis Detection Kit (Solarbio Life Science) was used to determine the cell apoptosis rate according to the manufacturer’s instructions. In 6-well plates, GES-1 cells were seeded. The treatment of the cells was consistent with that of the morphological examination. Then, we washed them with cold PBS three times and used a cell scraper to carefully collect cells in each group before staining with Annexin V-FITC and PI at room temperature (RT) for 15 min. Finally, flow cytometry was used to detect apoptotic cells using a FACS flow cytometer (BD Biosciences, USA). The results were analyzed by FlowJo software (version 10.8.1, TreeStar, USA).

### ROS detection

For testing the intracellular ROS, 10^6^ GES-1 cells were cultured on a 12-well plate and placed in an incubator at 37 °C under 5% CO_2_. After CAP pretreatment for 2.5 hr and EtOH incubation for 1.5 hr, the intracellular ROS level was measured. 1 ml of DCFH-DA (10 μM) was added and incubated in the dark at 37 °C for 30 min. Then, we collected cells and washed them with PBS three times, and finally, the ROS activity was detected by inverted fluorescence microscope and flow cytometry. For the detection of ROS in gastric tissue, 5 µm cryosections of frozen gastric tissues were stained with 10 µM Dihydroethidium (DHE) for 30 min in the dark at RT. Pictures were taken at 200-fold magnification and quantification by Ipp6.0.

### Measurements of MDA, SOD, and CAT

GES-1 cells with good condition were cultured in 6-well plates. After the four groups described above are constructed, the cells were collected and counted, and detected by chemiluminescence according to the kit instructions. For in vitro cell experiments, the cells were collected into the centrifuge tube, and then the supernatant was discarded after centrifugation. 1 mL extraction solution was added for every 5 million cells and ultrasonic crushing was performed (power 200 w, ultrasonic 3 s, interval 10 s, repeat 30 times). Centrifuge 8000 g at 4 °C for 10 min, take supernatant and put it on ice for test. Gastric tissue of each rat was homogenized in freshly prepared ice-cold 0.05  M potassium phosphate buffer (pH 7.4) with 20 times dilution. The supernatant was obtained by centrifuging the tissue homogenate at 15,000×g at 4 °C for 10 min, which was used for the evaluation of the gastric MDA level, SOD, and CAT activities, and the results were expressed as nmol/mg protein or U/mg protein. SOD activity was determined using a SOD assay kit (BC0175, Solarbio, China); CAT activity was evaluated using a CAT assay (BC0205, Solarbio, China) and the MDA level was also determined by an MDA assay (BC0025, Solarbio, China).

### Mitochondrial staining and branch length analysis

After different treatments on GES-1 lived in confocal dishes, PK Mito Orange (PKMO-1, Genvivo Biotech, China) and DAPI were added to the medium, followed by incubation at 37 °C for 15 min. The culture medium was then discarded and the cells washed gently three times with cell medium. Images were obtained under the Leica STELLARIS 5 Confocal Microscope Platform. Quantitative analysis of the mean branch length of mitochondria was calculated using MiNA software developed by ImageJ.

### Detection of mitochondrial membrane potential (*MMP,* △Ψm)

After the treatment, △Ψm of GES-1 cells were measured with a mitochondrial membrane potential detection kit (CA1310, Solarbio, China). In detail, after pretreatment with CAP for 2.5 h, cells were treated with 5% EtOH for 10 min or CCCP, a positive control reagent in the kit, for 20 min. Cells were collected and incubated in a cell incubator at 37 °C with JC-10 staining solution for 20 min. Then, we followed the instructions of the kit and analyzed it by flow cytometry.

### ATP detection

After pretreatment with CAP for 2.5 hr, GES-1 cells were treated with 5% EtOH for 10 min, ATP in cell supernatant and intracellular were detected using an ATP test kit (S0026, Beyotime, China). The principle of the kit is that ATP is needed to generate fluorescence when firefly luciferase catalyzes luciferase. The fluorescence production is detected by the chemiluminescence instrument, which is proportional to the concentration of ATP within a certain range.

### Lactic acid (LA) detection

After CAP pretreatment for 2.5 hr and EtOH incubation for 1.5 hr, the intracellular LA level was measured using a lactic acid assay kit (A019-2-1, Nanjing Jiancheng Bioengineering Institute, China). Follow the kit instructions and test at 530 nm (EnSpire Multilabel Reader).

### Network targets analysis of CAP

Network targets analysis was performed to obtain the potential mechanism of effects of CAP on ROS (Guo et al., 2019; Zhou et al., 2022 ; Li, 2015). Biological effect profile of CAP was predicted based on our previous network-based algorithm: drugCIPHER (Zhao and Li, 2010; Zhang et al., 2022a; Lin et al., 2017; Xu et al., 2023; Zhang et al., 2012; Zhou et al., 2021). Enrichment analysis was conducted based on R package clusterProfiler v4.9.1 and pathways or biological processes enriched with significant p-value less than 0.05 (Benjamini-Hochberg adjustment) were remained for further studies. Then pathways or biological processes related to ROS and significantly enriched were filtered and classified into three modules, including ROS, inflammation and immune. Network targets of CAP against ROS were constructed based on above analyses.

### Proteomics and omics analysis

GES-1 cells were divided into three groups: Ctrl; EtOH and CAP + EtOH，and each group also had three biological replicates. After medium or 8 μM CAP pretreatment for 2.5 hr and medium or 5% EtOH incubation for another 1.5 hr, respectively, they were washed with PBS and 300 μl SDT lysis solution was added into a 10 cm petri dish. In order to obtain the differential expression genes (DEGs), this project adopted the quantitative proteomics technology of TMT, which mainly included two stages of mass spectrometry experiment and data analysis. The DEGs (Fold change ≥1.2, *p*-value ≤0.05) were enriched by GO and KEGG through the DAVID database (https://david.ncifcrf.gov/), and the bioinformatic analysis was performed using the OmicStudio tools at https://www.omicstudio.cn/tool.

### Western blotting

At the specified time after processing, the cells and supernatant were collected directly into the centrifuge tube. After centrifugation at 6,500 rpm for 5 min, cells were washed three times with PBS and lysed in RIPA buffer. The protein samples were separated by 12% SDS-PAGE (80 V, 30 min and 130 V, 55 min), transferred to nitrocellulose (100 V, 105 min), sealed with 5% skim milk (30 min at RT), probed with the indicated primary antibodies (overnight at 4 °C), washed three times with TBST (120 rpm, 6 min) and exposed to species-specific horseradish peroxidase (HRP)-conjugated secondary antibodies (60 rpm, 60 min). After washing three times with TBST, immunoreactive bands were visualized by enhanced chemiluminescence. The gray value of protein was analyzed by ImageJ software. GAPDH and ACTB served as the reference for equivalent total protein volume. WB of rat tissue was operated on ice at 4 °C during the whole process. The tissue was cut into fine fragments and the high efficiency RIPA tissue lysate (R0010, Solarbio, China) was added at the ratio of 200 µl lysate per 20 mg gastric tissue. The lysate was homogenized with a glass homogenizer until fully lysed. After centrifuging at 12,000 rpm for 5 min, the supernatant was taken to conduct subsequent target protein detection.

### Quantitative PCR (RT-qPCR)

Total RNA from the cells was extracted using a QIAzol lysis reagent. The RNA was reverse-transcribed into cDNA using TransScript first-strand cDNA synthesis supermix according to the manufacturer’s instruction (AE301-02, TransGen Biotech, China). Hieff UNICON Universal Blue qPCR SYBR Green Master Mix was used for qPCR (11184ES03, Yeasen, China). The relative amount of target gene mRNA was normalized to that of *GAPDH* mRNA in the same sample. The primers’ sequences used in cell experiments were designed through PrimerBank (https://pga.mgh.harvard.edu/primerbank/) and the primers’ sequences of PKM2 and LDHA were designed through a reference (Sun et al., 2022). The sequences of qPCR primers in the rat experiments were listed using *r-ACTB* and etc. All the primer sequences of RT-qPCR were listed in Table 1.

**Table 1.** The primer sequences of RT-qPCR.

Genes	Forward Sequences (5’–3’)	Reverse Sequences (5’–3’)
*GAPDH* *GSS* *HMOX1* *NQO1* *TXN* *KEAP1* *PKM2* *LDHA r-ACTB r-NQO1* *r-HMOX1*	GAA GGT GAA GGT CGG AGT C GGG AGC CTC TTG CAG GAT AAA AAG ACT GCG TTC CTG CTC AAC GAA GAG CAC TGA TCG TAC TGG C GTG AAG CAG ATC GAG AGC AAG CTG GAG GAT CAT ACC AAG CAG G GTG CCG CCT GGA CAT TGA CTC GCT CAT CGT CTC AAA CCC AGT GG GAA GTG TGA CGT TGA CAT CCG AAG CGT CTG GAG ACT TCT GGG AAG AGG CTA AGA CCG CCT TC	GAA GAT GGT GAT GGG ATT TC GAA TGG GGC ATA GCT CAC CAC AAA GCC CTA CAG CAA CTG TCG GGA TAC TGA AAG TTC GCA GGG CGT GGC TGA GAA GTC AAC TAC TA GGA TAC CCT CAA TGG ACA CCA C ATT CAG CCG AGC CAC ATT CAT CC ACT CCC AGC CTT TCT CCC ATC AG TGC TGA TCC ACA TCT GCT GGA CCT CTG GCG AAG AAA CTC TG GCA TAA ATT CCC ACT GCC AC

### Immunofluorescence staining and confocal microscopy

At the indicated times of treatment with CAP and EtOH, GES-1 cells were fixed in 4% paraformaldehyde (PFA) for 15 min, permeabilized with 0.5% Triton X-100 for 10 min, blocked with 1% bovine serum albumin (BSA) for 20 min, and then probed with specific antibodies (dilution ratio of the primary antibody of NRF2, 1:100; secondary antibody, 1:500). Then, 10 mg/mL DAPI was used to label nuclei. Images were captured with a confocal laser scanning microscope system. The colocalization of signals from two channels was detected using ImageJ software.

### Nucleoplasm separation

After different treatments on GES-1 cultured in 6-well plates, cells were centrifuged at 6000 rpm for 5 min and discarded the supernatant. All subsequent operations were performed on ice at 4 °C. First, the cells were decomposed for 5 min by adding 100 μl of cytoplasmic lysate (containing 10 mM HEPES, 10 mM KCl, 0.1 mM EDTA, 0.5% NP-40 and adding extra 1:1000 PMSF and 1:2000 DTT). Then blow 200 times with a 200 µl Eppendorf. After centrifugation at 500 g for 5 min, the supernatant was the cytoplasmic lysate. Finally, 200 µl of NP40-free lysate was added for nuclear washing and precipitation for 3 times (discard the supernatant). After mixing with an oscillator, the mixture was centrifuged at 500 g for 5 min to obtain the nuclear proteins. All samples were boiled at 99 °C for 10 min for western blot experiments.

### Coimmunoprecipitation (Co-IP)

First, 3 mg pcDNA3.1 or Keap1-FLAG plasmid with polyethylenimine (PEI) reagent was transfected into 293T cells (5×10^6^ cells/ml). After 24 hr, the cells were treated with 32 μM capsaicin for 2.5 hr and then suspended with phosphate-buffered saline (PBS) and centrifuged at 6500 rpm for 5 min to collect cell precipitate. The cells were lysed in 700 ml lysis buffer (50 mM Tris-HCl, pH = 7.5, 150 mM NaCl, 1% NP40, and 0.5% Triton X-100), and the lysates were incubated with anti-FLAG affinity matrix at 4 °C for 10 hr. The lysates were washed six times with PBS, eluted with glycine buffer (pH = 2), boiled in SDS loading buffer, and analyzed by WB. For the ubiquitin experiment, 3 mg Ub-K48-Myc and 3 mg VR1012 or NRF2-HA plasmids with PEI were co-transfected into 293T. After 36 hr, the cells were treated with 32 μM capsaicin for 2.5 hr. The rest of the steps were the same except that anti-HA affinity matrix was used.

### Pull-down

Purified Kelch-Strep protein (10 μg) was incubated with 10 μL of specific anti-Strep affinity matrix for a duration of 4 hr at 4 °C. Solutions containing 32 μM and 100 μM CAP were prepared, and 1 μg of NRF2 protein was added to each reaction system. The reaction systems were further incubated at 4 °C for another 2 hr. Subsequently, the unbound proteins were subjected to wash steps consistent with Co-IP procedures, followed by result assessment through western blot experiments.

### The degradation of NRF2

To test the degradation of NRF2, the drugs CHX and PS-341 were used. GES-1 cells were incubated with or without 8 μM CAP for 3 hr, followed by treatment with CHX (50 μg/mL) for 15 min or 30 min. Total cell lysates were collected and analyzed by WB for total NRF2 protein expression. What’s more, GES-1 cells were incubated with or without PS-341(100 nM) and CAP (8 μM) for 3 hr to compare the activation of NRF2 between co-incubation and single drug use. The total amount of NRF2 was quantified with ImageJ.

### Surface plasma resonance (SPR)

When the Human KEAP-1 Protein was fixed on the metal surface of the chip as a ligand protein, the mobile phase containing the analytical substance (NRF2 or Capsaicin) flowed through the stationary phase. The experiment was conducted in strict accordance with the relevant standard procedures of SPR. The analysis software used for the results of this experiment was TraceDrawer (Ridgeview Instruments ab, Sweden), and the analysis method was the One-to-One analysis model. The design of experimental methods and parameters involved are shown in Table 2:

**Table 2.** Experimental method design and parameters.

Project details		
Ligand	Human KEAP-1 Protein	
Analyte	Capsaicin, NRF2	
Running buffer	PBS (pH7.4), 1% DMSO PBS (pH7.4)	
Regeneration solution	10 mM Glycine-HCl	
Association and dissociation flow rate	20 μl/min, Association 240 s, Dissociation 360 s	
Temperatures	25 °C	
Sensor Chip	Sensor Chip COOH	Nicoya
OpenSPR	OpenSPRTM	Nicoya

### Bio-layer interferometry assay (BLI)

The Octet RED96e system (Molecular Device, ForteBIO) is ideally suited for the characterization of protein-small molecule binding kinetics and binding affinity. The protein Kelch (WT) and Kelch (Mut) were purified, biotinylated, and diluted to 50  μg/ml. The proteins were then immobilized on SSA sensors. The sensors were then blocked, washed, and moved into wells containing various concentrations of the test compound of CAP in kinetic buffer. Program settings default to programs that bind proteins and small molecules in the system. The binding signals were identified and the results were analyzed using the OctetHT V10.0 software. R^2^ is an estimate of the goodness of fit with values close to 1 indicating an excellent fit.

### Cellular thermal shift assay (CETSA)

GES-1 cells on 24-well plates were treated with 1640 medium containing Capsaicin (8 μM) or 0.1% DMSO for 2.5–3 hr. Cells were collected from each group and divided into seven PCR tubes on average. The cells were heated at the specified temperature (46–67 °C) for 3 min and cooled at room temperature. The cells were lysed with liquid nitrogen and freeze-thawed four times. The cell lysates were centrifuged at 12,000 rpm for 30 min at 4 °C. The protein supernatant was analyzed by WB, and was quantified by ImageJ software.

### Mass spectrometry (MS-MS)

The final concentration of capsaicin was 50 μM (DMSO less than 0.5% by volume) after 20 μg Keap1 protein solution was added with 2 μl capsaicin (1 mM) and was dissolved in HEPES (pH = 7.4). Incubate at 30 °C for 3 hr in the dark. Separated by 10% SDS-PAGE gel (80 V 30 min and 130 V 20 min); the overexpressed KEAP1 was detected by Coomassie Bright Blue staining, and the corresponding bands were removed from the gel for intra-gel digestion as described. In short, the gel tablets were reduced with DTT (10 mM) and alkylated with iodoacetamide (50 mM). The rest of the process is followed and the digested protein samples are manipulated and analyzed by Novogene Co. Ltd., China.

### Computational simulations

The flexible docking between the KEAP1 Kelch domain and the small molecule CAP was performed by RosettaLigand (Meiler and Baker, 2006). The crystal structure of Kelch (PDB: 3WN7) and 3D model of CAP (ZINC1530575, https://zinc.docking.org/substances/ZINC000001530575/) were used as the input structures. The Rosetta software was obtained from https://www.rosettacommons.org and version number is 2021.36+release.57ac713 57ac713a6e1d8ce6f60269b3988b1adac1d96fc6. Rosetta Transform mover with the box size of 8.0 Å was used for global searching and the HighResDocker mover was used for fine-tuning the binding positions. 1000 docking poses were generated and the interface binding energies was calculated using the default Ref2015_wts score functions. The docking results were ranked by the binding energy and clustered for the representative conformations.

The 100 ns all-atom simulations on the complex of NRF2-KEAP1 or NRF2-CAP-KEAP1 were performed with the Gromacs 2019.6 package (Van Der Spoel et al., 2005). The initial poses of inhibitor-binding conformations were adopted from the docking results and from PDB 3WN7. The system was solvated in a box (81×81×81 Å3) with TIP3P waters and 0.15 M NaCl. The CHARMM36 force field was adopted, wherein the topologies of inhibitors were generated by the CHARMM_GUI Ligand Modeler (Kim et al., 2017). The energy minimizations were performed to relieve unfavorable contacts, followed by 10 ns equilibration steps. Subsequently, the simulations were performed at 300 K (velocity-rescale thermostat) and constant pressure (one bar, Parrinello-Rahman NPT ensemble). The nonbonded interaction cut-off for electrostatics calculations was set as 10 Å and the particle mesh Ewald (PME) method was used for calculation of long-range electrostatic interactions. LINCS constraints were applied to h-bonds and the time step was 2 fs. Throughout the trajectories, the representative binding conformations were clustered based on their structural similarities.

### Protein purification

The synthetic cDNA encoding Kelch (WT) and Kelch (Mut) were cloned into pET28a vector, respectively. The plasmid vectors were transformed into *E. coli* BL21 (DE3) to express proteins, which were induced by 0.4 mM isopropyl β-D-1-thiogalactopyranoside (IPTG) at 16 °C for 18 hr, followed by culturing at 37 °C until the OD600 value reached 0.6. The cells were isolated by centrifugation, resuspended and lysed in lysis buffer. The lysates were centrifuged for 60 min at 12,000 rpm to obtain the supernatant. Gravity flow purification of the proteins in the supernatant were obtained using Ni-affinity chromatography and eluted in buffer containing 200 mM imidazole. The final proteins were purified and validated by Superdex 75 10/300 GL resin.

### Hydrogen-deuterium exchange mass spectrometry (HDX-MS)

Kelch protein (1 mg/mL) was incubated with CAP (50 μM) or 0.2% DMSO in 4 °C overnight. The protein was equilibrated in D_2_O buffer at room temperature and quenched with ice-cold quench buffer (4 M Guanidine hydrochloride, 200 mM Citric acid, and 500 mM TECP in water solution at pH 1.8, 100% H_2_O) at indicated times, then immediately put the sample tube on ice. Then, 1 μM pepsin solution was added for digestion. At 2 min, the sample was placed into a Thermo-Dionex Ultimate 3000 HPLC system autosampler for injection.

For LC-MS/MS analysis, the peptides were separated by a 20 min gradient elution at a flow rate of 115 µl/min with a Thermo-Dionex Ultimate3000 HPLC system, which was directly interfaced with a Thermo Scientific Q Exactive mass spectrometer. Peptides were separated on a reverse phase column (Acquity UPLC BEH C18 column 1.7 µm, 2.1×50 mm, Waters, UK). Mobile phase consisted of 1% formic acid, and mobile phase B consisted of 100% acetonitrile and 1% formic acid. The Q Exactive mass spectrometer was operated in the data-dependent acquisition mode using Xcalibur 2.0.0.0 software and there was a single full-scan mass spectrum in the Orbitrap (350–2000 m/z, 70,000 resolution). The mass spectrometer was operated at a source temperature of 250 °C and a spray voltage of 3.0 kV. Peptic peptides were identified using an in-house Proteome Discoverer (Version PD1.4, Thermo Fisher Scientific, USA).

The search criteria were as follows: no enzyme was required; two missed cleavage was allowed; precursor ion mass tolerances were set at 20 ppm for all MS acquired in an Orbitrap mass analyzer; and the fragment ion mass tolerance was set at 0.02 Da for all MS2 spectra acquired. The peptide false discovery rate (FDR) was calculated using Percolator provided by PD. When the q value was smaller than 1%, the peptide spectrum match (PSM) was considered to be correct. FDR was determined based on PSMs when searched against the reverse, decoy database. Peptides only assigned to a given protein group were considered as unique. The FDR was also set to 0.01 for protein identifications. The deuterium exchange levels were determined by subtracting the centroid mass of undeuterated peptide from the centroid mass of deuterated peptide using HDExaminer (Version PD1.4, Thermo Fisher Scientific, USA).

### Preparation of IR-HSA@CAP NPs

500 μl CAP (20 mg/ml) was uniformly injected into 10 ml of HSA solution (2 mg/mL), and stirred for 10 min at 700–800 rpm/min at RT. 50% glutaraldehyde was diluted 100 times and 80 μl was injected into the flask. Keep stirring at the same speed for 3 hr. After termination of reaction, dialysis bag was used overnight to remove excess glutaraldehyde. HSA@CAP NPs were obtained and stored at 4 °C under shading. To obtain IR-HSA@CAP NPs, we added 40 μl of IRDye800 (5 mg/ml) into 700 μl of HSA@CAP NPs (2 mg/ml HSA). After incubation at RT for 1 hr, the solution was added with a 30 kDa protein concentration tube, and centrifuged at 12,000 rpm for 5 min. Some of the unbound antibody probes were centrifuged. Then eluted 4–5 times with 1X PBS (0.01 M) until there was no color in the collection tube. Protein concentration and labeling (DOL) were determined by measuring absorption at 280 nm and 780 nm.

### Particle size and Zeta potential

The morphology of the prepared IR-HSA@CAP NPs was observed by Transmission Electron Microscopy (TEM, TECNAI G2 F20). The sample was prepared as follows: IR-HSA@CAP NPs were diluted 150 times before ultrasonic treatment, so that the particles were evenly distributed in the solution. 10 μl sample solution was slowly dropped onto the surface of the copper mesh, covered with a protective cover to prevent dust pollution in the air, and waited for the sample solvent to fully volatilize at RT. The hydration particle size and potential of the samples were measured using Litesizer 500. 2–3 ml of aqueous solution diluted 1000 times by HSA@CAP and IR-HSA@CAP NPs were added into the plastic sample plate, and the hydration particle size and potential were detected at 25 °C. The same sample was measured at least three times in parallel to calculate the mean.

### High performance liquid chromatography (HPLC)

The peak time and area corresponding to the HPLC were used to calculate the capsaicin content in IR-HSA@CAP. HPLC procedure: sample size was 10 μl, the temperature of C18 was adjusted to 30 °C, acetonitrile (10–94%) and 0.1% phosphoric acid water were used as mobile phase and time range was 0~28 min. To evaluate the release of capsaicin in simulated gastric fluid (SGF: 3.2 g pepsin, 2 g NaCl, 7 ml concentrated hydrochloric acid in 1 L ddH_2_0, pH = 2.0), 200 μl of evenly mixed IR-HSA@CAP was added to 800 μl of freshly configured SGF/SPSS, and mixed at 37 °C. At the corresponding time point, it was evenly mixed with anhydrous methanol at 1:1 and added 1 ml mixed solution into an injection bottle for measurement.

### Endocytosis and in vivo imaging of IR-HSA@CAP NPs

In vivo experiments, healthy adult male Sprague-Dawley (SD) rats, weighing 230–260 g were used. The newly prepared IR-HSA@CAP NPs solution was diluted and added into the medium, and incubated with GES-1 cells for 1 hr, 2 hr, and 3.5 hr. After the supernatant was discarded, the cells were washed with PBS for five times, and the ICG fluorescence in the cells was detected by APC-Cv7-A channel of flow cytometry. SD rats (24 hr after fasting) were given 1 ml IR-HSA@CAP NPs by intragastric administration. The fluorescence signals of rats were detected after administration. At the same time, images of side position were photographed at 45 min and 60 min, and dissected major organs (heart, liver, spleen, lung, and kidney) and digestive system (stomach, large intestine, colon, and duodenum) in vitro to determine the specific location of CAP.

### Pathological examination of gastric tissue (H&E staining)

As previously mentioned, the stomach tissue is soaked in 4% paraformaldehyde tissue fixation solution for 24 hr. Then the stomach tissue was cleaned, dehydrated by different concentrations of ethanol solution, transparent in xylene, and finally embedded in paraffin. Sections of 3 µm thickness were prepared, dried and dewaxed, stained with hematoxylin and eosin (H&E), dehydrated and sealed, and any structural changes were detected microscopically (OLYMPUS, DP26).

### Detection of cytokines in gastric tissue (ELISA)

Interleukin (IL)-1β, IL-6, CXCL1/KC (IL-8), IL-10, and tumor necrosis factor-alpha (TNF-α) levels in the gastric tissue lysate were determined to assess gastric inflammation. ELISA kits from Solarbio, catalogue numbers SEKR-0002, SEKR-0005, SEKR-0014, SEKR-0006, and SEKR-0009 were used for IL-1β, IL-6, CXCL1, IL-10, and TNF-α, respectively following the manufacturer’s instructions.

### Protocol for normal mice and Nrf2-KO mice

To ensure gastric emptying, mice were subjected to a 24 hr fasting period. Subsequently, they were administered SPSS or IR-HSA@CAP NPs. After a 45 min pretreatment phase, the mice were exposed to 80% EtOH for an additional 1 hr. Following this, the animals were humanely euthanized, and the gastric tissue samples were collected for subsequent experiments and analyses. Precise dosages administered were as follows: IR-HSA@CAP nanoparticles used were prepared to contain 1 mg/kg of CAP and 2 mg/kg of HSA, as quantified by HPLC three times.

### Statistical analysis

The differences in the mean values between the two groups were analyzed using the student’s t-test, while the differences among multiple groups were analyzed using a one-way ANOVA test in the GraphPad Prism software. Therefore, we checked and proofread the statistical analysis. Statistical significance was indicated at a *p*-value <0.05. (ns, no significance; *p<0.05; **p<0.01; ***p<0.001; ****p<0.0001).

## Data Availability

All data generated or analysed during this study are included in the manuscript, figures, figure supplements and source data files.

## Additional files

MDAR checklist

Source data 1. Raw quantification data.

## Appendix 1

**Appendix 1—key resources table:**

Reagent type (species) or resource	Designation	Source or reference	Identifiers	Additional information
Strain, strain background (*Rattus norvegicus*)	Sprague Dawley Rats (CD IGS) Rats (SD rats)	Beijing Vital River Laboratory Animal Technology Co., Ltd.	Cat. # 101	
Strain, strain background (*Mus musculus*)	C57BL/6Smoc (C57BL/6)	Shanghai Model Organisms Center, Inc	Cat. # SM-001	
Strain, strain background (*Mus musculus*)	Nrf2-knockout (Nrf2-KO) mice	Shanghai Model Organisms Center, Inc	Cat. # NM-KO-190433	
Cell line (*Homo sapiens*)	293T cells	The American Type Culture Collection (ATCC)	Cat# CRL-3216	
Cell line (*Homo sapiens*)	UC-MSC cells	ATCC	Cat#PCS-500–010	
Cell line (*Mus musculus*)	RAW 264.7	ATCC	Cat#TIB-7	
Cell line (*Homo sapiens*)	GES-1 cells	Procell Life Science & Technology Co., Ltd.	Cat#CL-0563	
Cell line (*Homo sapiens*)	KEAP1 Knockout 293T cells	ABclonal Biotechnology Co., Ltd	Cat#RM02350	
Antibody	Anti-HA-Tag (Mouse monoclonal)	ABclonal Biotechnology Co., Ltd	Cat# AE008	WB (1:10000)
Antibody	Anti-Strep II-Tag (Mouse monoclonal)	ABclonal Biotechnology Co., Ltd	Cat# AE066	WB (1:10000)
Antibody	Anti-MYC-Tag (Mouse monoclonal)	ABclonal Biotechnology Co., Ltd	Cat# AE010	WB (1:5000)
Antibody	Anti-DYKDDDDK -Tag (Rabbit monoclonal)	Proteintech Group, Inc	Cat# 80010–1-RR	WB (1:10000)
Antibody	Anti-Actin (Mouse monoclonal)	Proteintech Group, Inc	Cat# 66009–1-Ig	WB (1:1000)
Antibody	Anti-GAPDH (Mouse monoclonal)	Proteintech Group, Inc	Cat# 60004–1-Ig	WB (1:20000)
Antibody	Anti- NRF2/NFE2L2 (Rabbit polyclonal)	Proteintech Group, Inc	Cat# 16396–1-AP	WB (1:1000)
Antibody	Anti-NRF2 (Rabbit monoclonal)	Cell Signaling Technology, Inc	Cat# 12721	WB (1:1000)
Antibody	Anti-GSS (Cat No. Rabbit polyclonal)	Proteintech Group, Inc	Cat# 15712–1-AP	WB (1:1000)
Antibody	Anti-HO-1/HMOX1 (Rabbit polyclonal)	Proteintech Group, Inc	Cat# 10701–1-AP	WB (1:1000)
Antibody	Anti-Thioredoxin 1/TXN (Rabbit polyclonal)	Proteintech Group, Inc	Cat# 14999–1-AP	WB (1:5000)
Antibody	Anti-KEAP1(Rabbit polyclonal)	Proteintech Group, Inc	Cat# 30041–1-AP	WB (1:1000)
Antibody	FITC Goat anti-Rabbit IgG (H+L)	SIMUBIOTECH	Cat# S2003	IF (1:2000)
Recombinant DNA reagent	pcDNA3.1-Flag	Addgene	Cat#210342	
Recombinant DNA reagent	pcDNA3.1-KEAP1-FLAG	MiaoLing Plasmid Platform	Cat# P8893	
Recombinant DNA reagent	pCMV-NFE2L2 (human)-HA-Neo	MiaoLing Plasmid Platform	Cat# P45110	
Recombinant DNA reagent	VR1012	This paper		Construction information described in the Materials and methods section
Recombinant DNA reagent	VR1012- NFE2L2	This paper		Construction information described in the Materials and methods section
Recombinant DNA reagent	pUb-K48-Myc	This paper		Construction information described in the Materials and methods section
Recombinant DNA reagent	pET-28a-KEAP1(Kelch)-Flag	This paper		Construction information described in the Materials and methods section
Recombinant DNA reagent	pET-28a-KEAP1(Kelch)-Strep	This paper		Construction information described in the Materials and methods section
Recombinant DNA reagent	pET-28a-KEAP1(Mut)-Strep	This paper		Construction information described in the Materials and methods section
peptide, recombinant protein	Recombinant Human KEAP1 Protein	Sino Biological Inc	Cat#11981-HNCB	
peptide, recombinant protein	Recombinant Human Nrf2 protein	Abcam	Cat#ab132356	
Commercial assay or kit	pEASY-Basic Seamless Cloning and Assembly Kit	TransGen Biotech, China	Cat#CU201-02	
Commercial assay or kit	BCA Protein Assay Kit	Beyotime Biotechnology	Cat#P0012	
Commercial assay or kit	ATP test kit	Beyotime Biotechnology	Cat#S0026	
Commercial assay or kit	Reactive Oxygen Species Assay Kit	Solarbio Life Science	Cat#CA1410	
Commercial assay or kit	CCK-8 Cell Proliferation And Cytotoxicity Assay Kit	Solarbio Life Science	Cat#CA1210	
Commercial assay or kit	Annexin V-FITC Apoptosis Detection Kit	Solarbio Life Science	Cat#CA1020	
Commercial assay or kit	Anti-HA Affinity Matrix	Roche	Cat#11815016001	
Commercial assay or kit	Anti-Flag affinity matrix	Sigma	Cat#A2220	
Commercial assay or kit	enhanced chemiluminescence (ECL) reagent	Epizyme Biomedical Technology Co., Ltd	Cat#SQ201	
Commercial assay or kit	SOD assay Kit	Solarbio Life Science	Cat#BC0175	
Commercial assay or kit	CAT assay Kit	Solarbio Life Science	Cat#BC0205	
Commercial assay or kit	MDA assay Kit	Solarbio Life Science	Cat#BC0025	
Commercial assay or kit	Mitochondrial Membrane Potential Kit(JC-10 Assay)	Solarbio Life Science	Cat#CA1310	
Commercial assay or kit	PK Mito Orange	Genvivo Biotech, China	Cat#PKMO-1	
Commercial assay or kit	Lactic Acid assay kit	Nanjing Jiancheng Bioengineering Institute	Cat#A019-2-1	
Commercial assay or kit	EasyScript First-Strand cDNA Synthesis SuperMix	TransGen Biotech, China	Cat#AE301-02	
Commercial assay or kit	Hieff UNICON Universal Blue qPCR SYBR Green Master Mix	Yeasen Biotechnology (Shanghai) Co., Ltd.	Cat#11184ES03	
Commercial assay or kit	ELISA kit of Interleukin (IL)- 1β	Solarbio Life Science	Cat#SEKR-0002	
Commercial assay or kit	ELISA kit of IL-6	Solarbio Life Science	Cat#SEKR-0005	
Commercial assay or kit	ELISA kit of CXCL1/KC(IL-8)	Solarbio Life Science	Cat#SEKR-0014	
Commercial assay or kit	ELISA kit of IL-10	Solarbio Life Science	Cat#SEKR-0006	
Commercial assay or kit	ELISA kit of tumor necrosis factor-alpha (TNF-α)	Solarbio Life Science	Cat#SEKR-0009	
Chemical compound, drug	Capsaicin (CAP)	Solarbio Life Science	Cat# IC0060	Used in vitro
Chemical compound, drug	Capsaicin (CAP)	MCE	Cat# HY-10448	Used in vivo
Chemical compound, drug	anhydrous ethanol (EtOH)	Tianjin Jiangtian Chemical Technology Co. LTD	Cat#268	
Chemical compound, drug	albumin from human serum (HSA)	Sigma	Cat#A3782	
Chemical compound, drug	DL-dithiothreitol (DTT)	MCE	Cat#HY-15917	
Chemical compound, drug	Cycloheximide (CHX)	MCE	Cat#HY-12320	
Chemical compound, drug	Bortezomib (PS-341)	MCE	Cat#HY-10227	
Chemical compound, drug	IRDye 800CW NHS Ester	LI-COR Biosciences	Cat#929–70020	
Software, algorithm	ImageJ	NIH	RRID:SCR_003070	
Software, algorithm	GraphPad Prism	GraphPad Software, Inc	RRID:SCR_002798	
Other	Dimethyl sulfoxide (DMSO)	Sigma	Cat#D8418	See Materials and Methods, Section 1
Other	Fetal Bovine Serum (FBS)	Gibco	Cat#10099–141	See Materials and Methods, Section 1
Other	Lipofectamine 2000	Thermo Fisher	Cat#11668027	See Materials and Methods, Section 1
