# Supplementary figures and images for: Capsaicin acts as a novel NRF2 agonist to suppress ethanol induced gastric mucosa oxidative damage by directly disrupting the KEAP1-NRF2 interaction
Source: eLife. 2026 Jun 9;13:RP97632. doi: 10.7554/eLife.97632 (PMC13249423; doi:10.7554/eLife.97632)

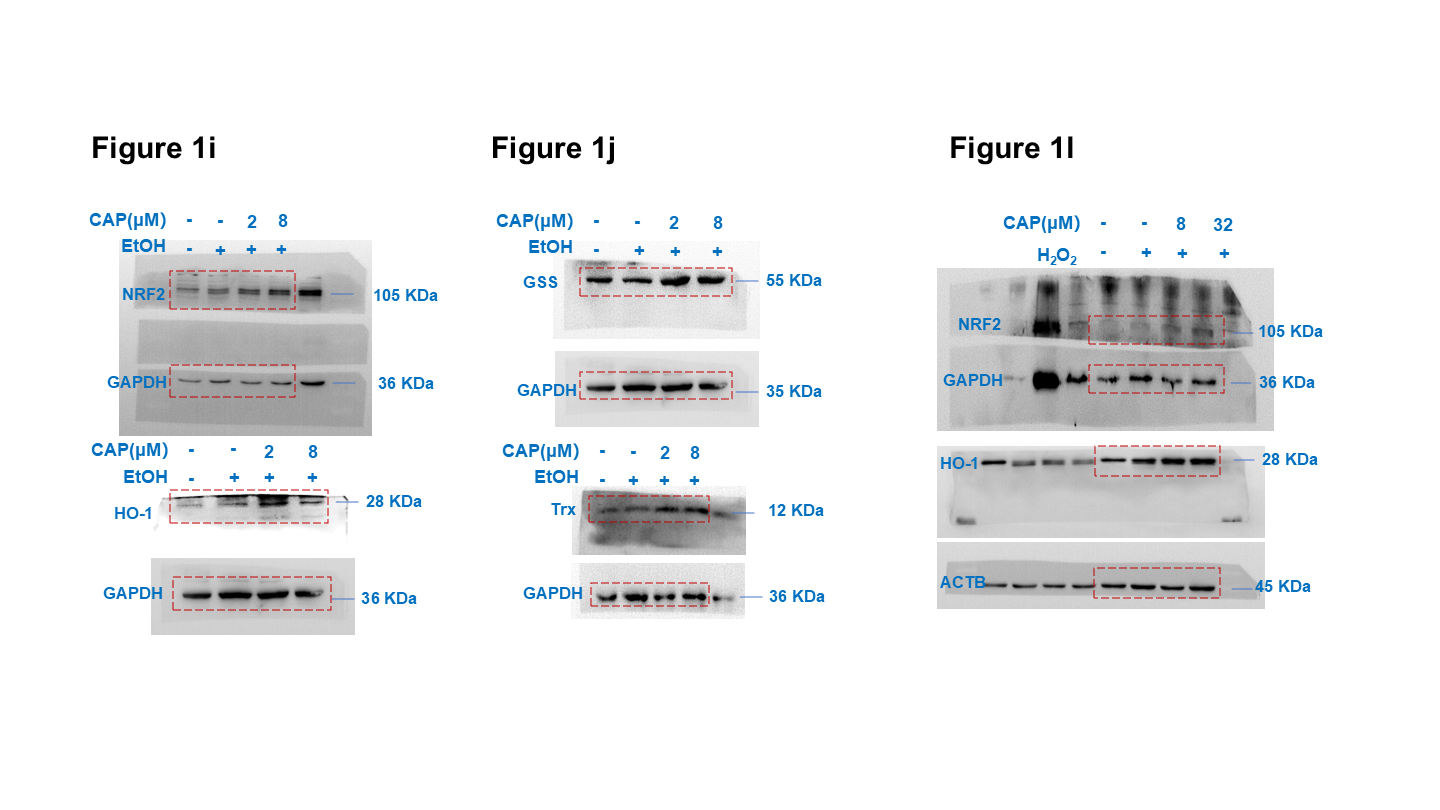

Supplement: Figure 1—source data 1. [file elife-97632-fig1-data1.zip › Figure 1-source data 1.tiff]

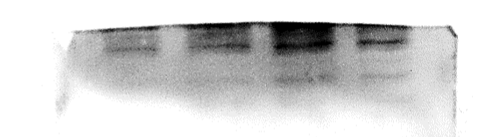

Supplement: Figure 1—source data 2. [file elife-97632-fig1-data2.zip › Figure 1-source data 2/Figure-1i HO-1.tif]

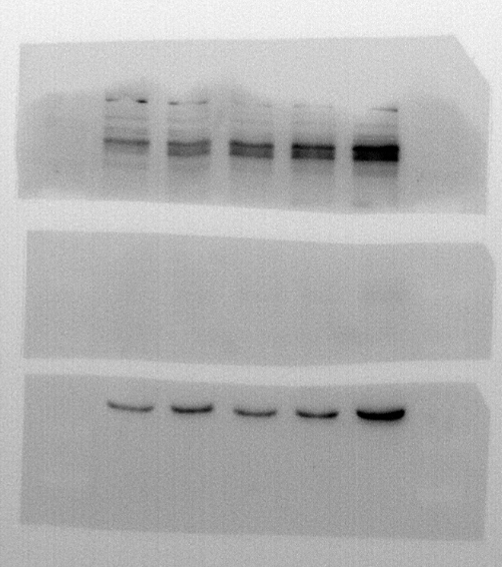

Supplement: Figure 1—source data 2. [file elife-97632-fig1-data2.zip › Figure 1-source data 2/Figure-1i NRF2 GAPDH.tif]

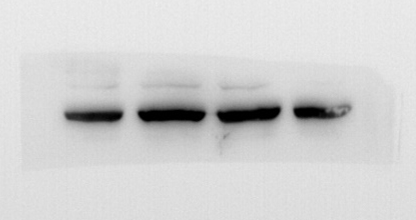

Supplement: Figure 1—source data 2. [file elife-97632-fig1-data2.zip › Figure 1-source data 2/Figure-1j GAPDH.tif]

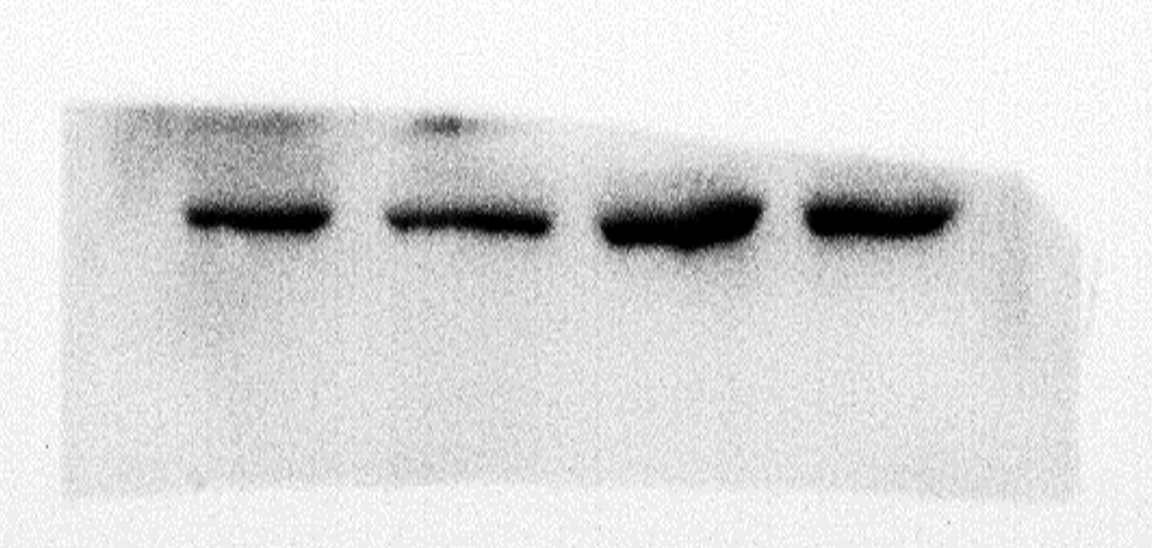

Supplement: Figure 1—source data 2. [file elife-97632-fig1-data2.zip › Figure 1-source data 2/Figure-1j GSS.tif]

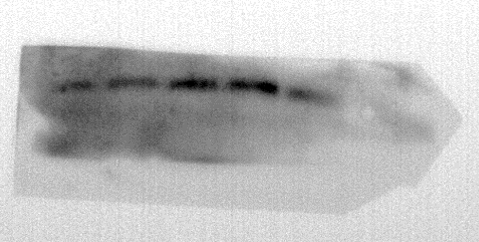

Supplement: Figure 1—source data 2. [file elife-97632-fig1-data2.zip › Figure 1-source data 2/Figure-1j Trx.tif]

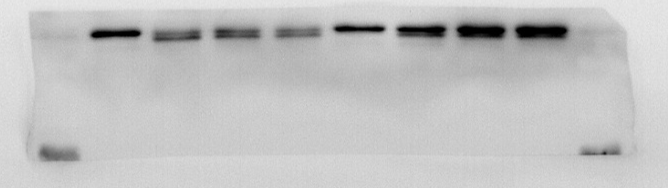

Supplement: Figure 1—source data 2. [file elife-97632-fig1-data2.zip › Figure 1-source data 2/Figure-1l HO-1.tif]

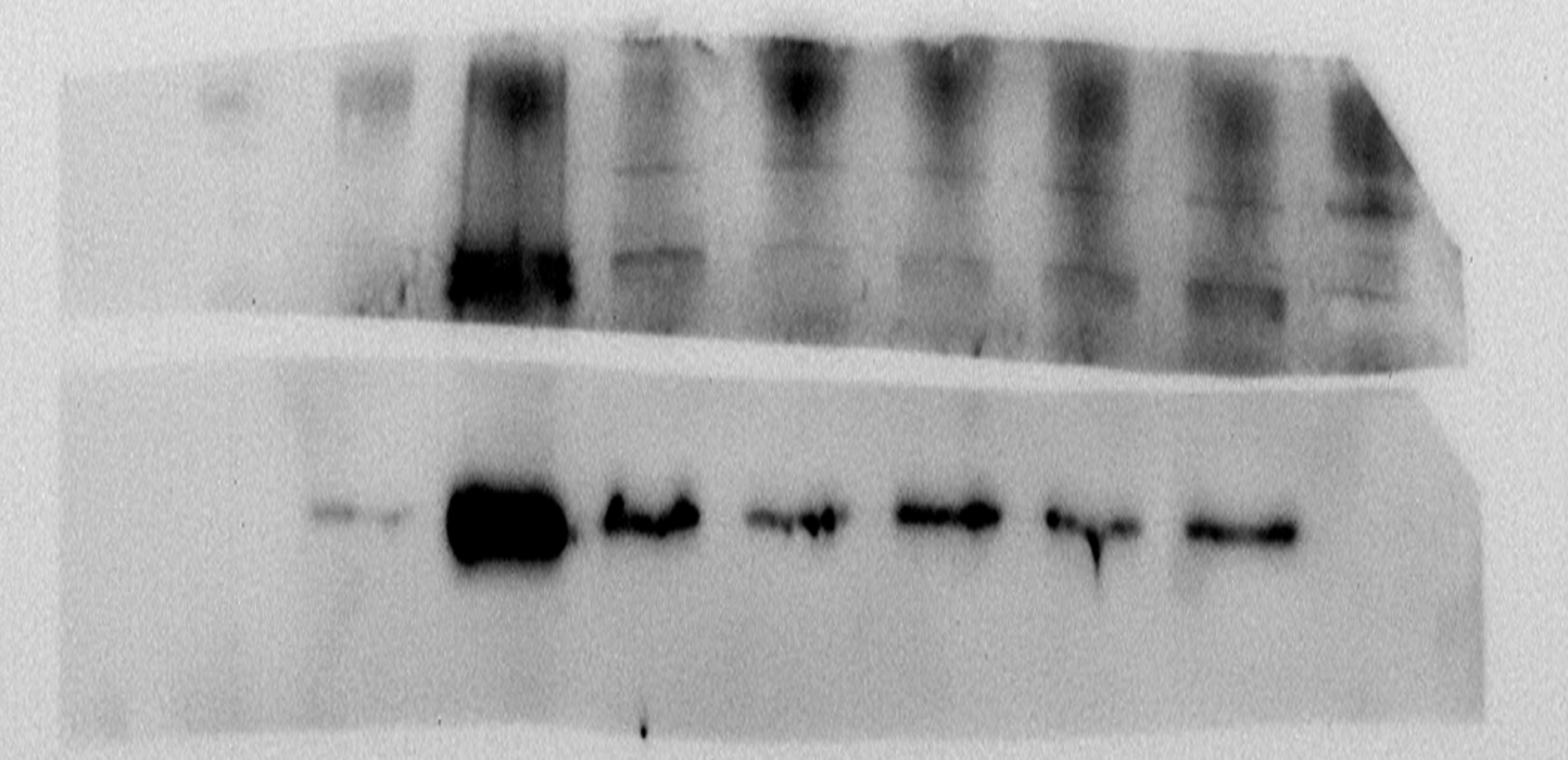

Supplement: Figure 1—source data 2. [file elife-97632-fig1-data2.zip › Figure 1-source data 2/Figure-1l NRF2 GAPDH.tif]

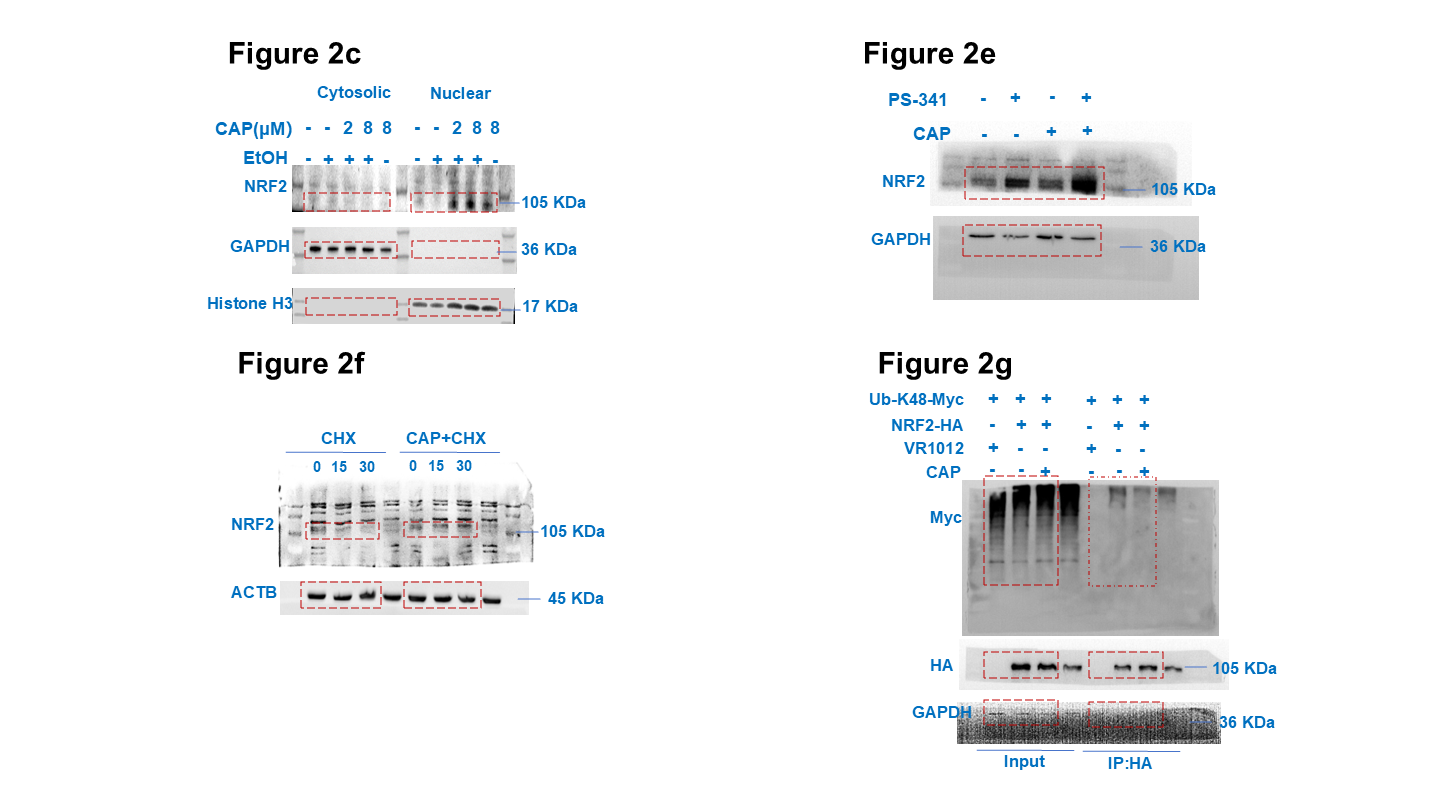

Supplement: Figure 2—source data 1. [file elife-97632-fig2-data1.zip › Figure 2-source data 1.tiff]

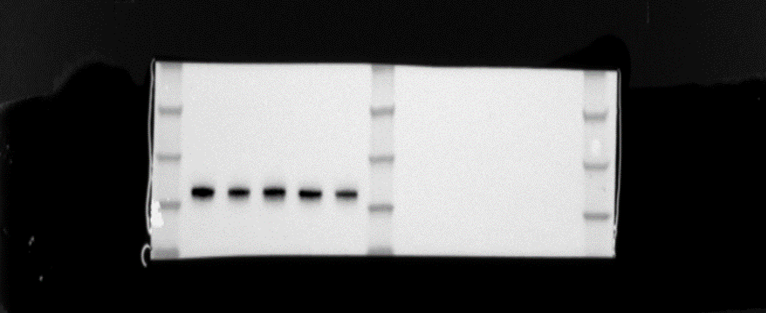

Supplement: Figure 2—source data 2. [file elife-97632-fig2-data2.zip › Figure 2-source data 2/Figure-2c GAPDH.tif]

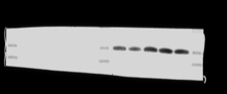

Supplement: Figure 2—source data 2. [file elife-97632-fig2-data2.zip › Figure 2-source data 2/Figure-2c Histone H3.tif]

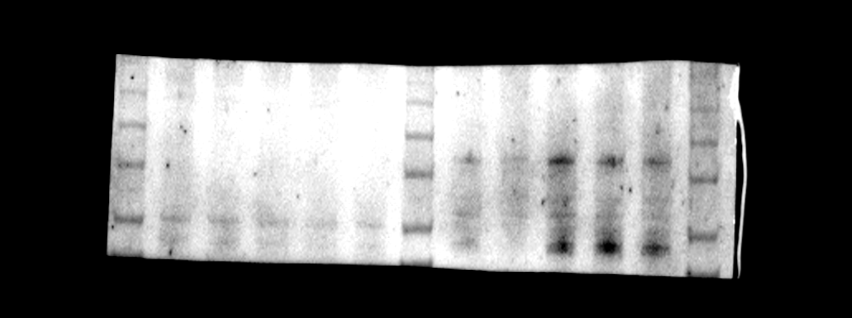

Supplement: Figure 2—source data 2. [file elife-97632-fig2-data2.zip › Figure 2-source data 2/Figure-2c NRF2.tif]

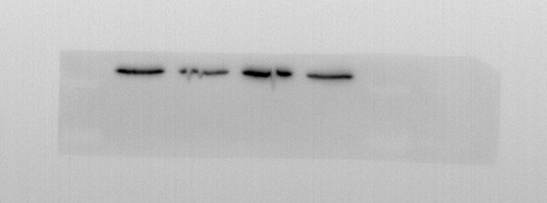

Supplement: Figure 2—source data 2. [file elife-97632-fig2-data2.zip › Figure 2-source data 2/Figure-2e GAPDH.tif]

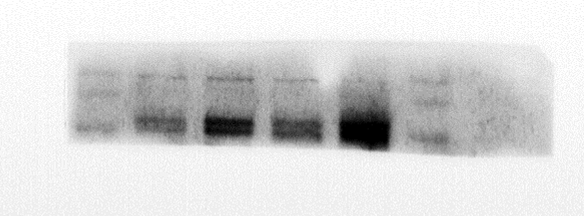

Supplement: Figure 2—source data 2. [file elife-97632-fig2-data2.zip › Figure 2-source data 2/Figure-2e NRF2.tif]

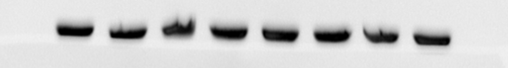

Supplement: Figure 2—source data 2. [file elife-97632-fig2-data2.zip › Figure 2-source data 2/Figure-2f ACTB.tif]

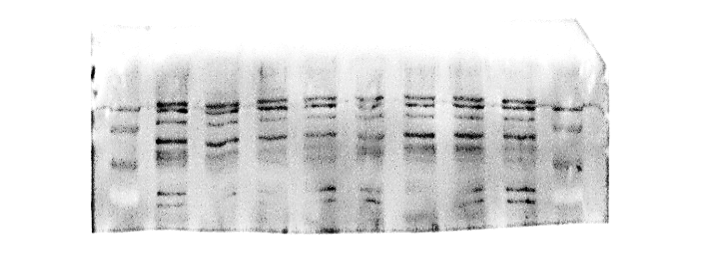

Supplement: Figure 2—source data 2. [file elife-97632-fig2-data2.zip › Figure 2-source data 2/Figure-2f NRF2.tif]

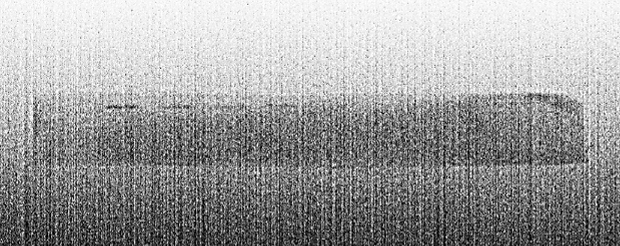

Supplement: Figure 2—source data 2. [file elife-97632-fig2-data2.zip › Figure 2-source data 2/Figure-2g GAPDH.tif]

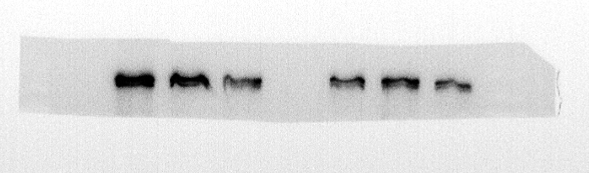

Supplement: Figure 2—source data 2. [file elife-97632-fig2-data2.zip › Figure 2-source data 2/Figure-2g HA.tif]

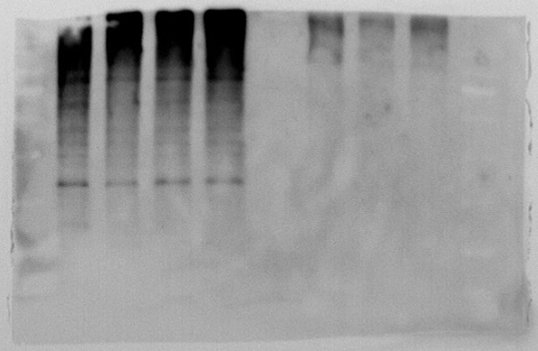

Supplement: Figure 2—source data 2. [file elife-97632-fig2-data2.zip › Figure 2-source data 2/Figure-2g Myc.tif]

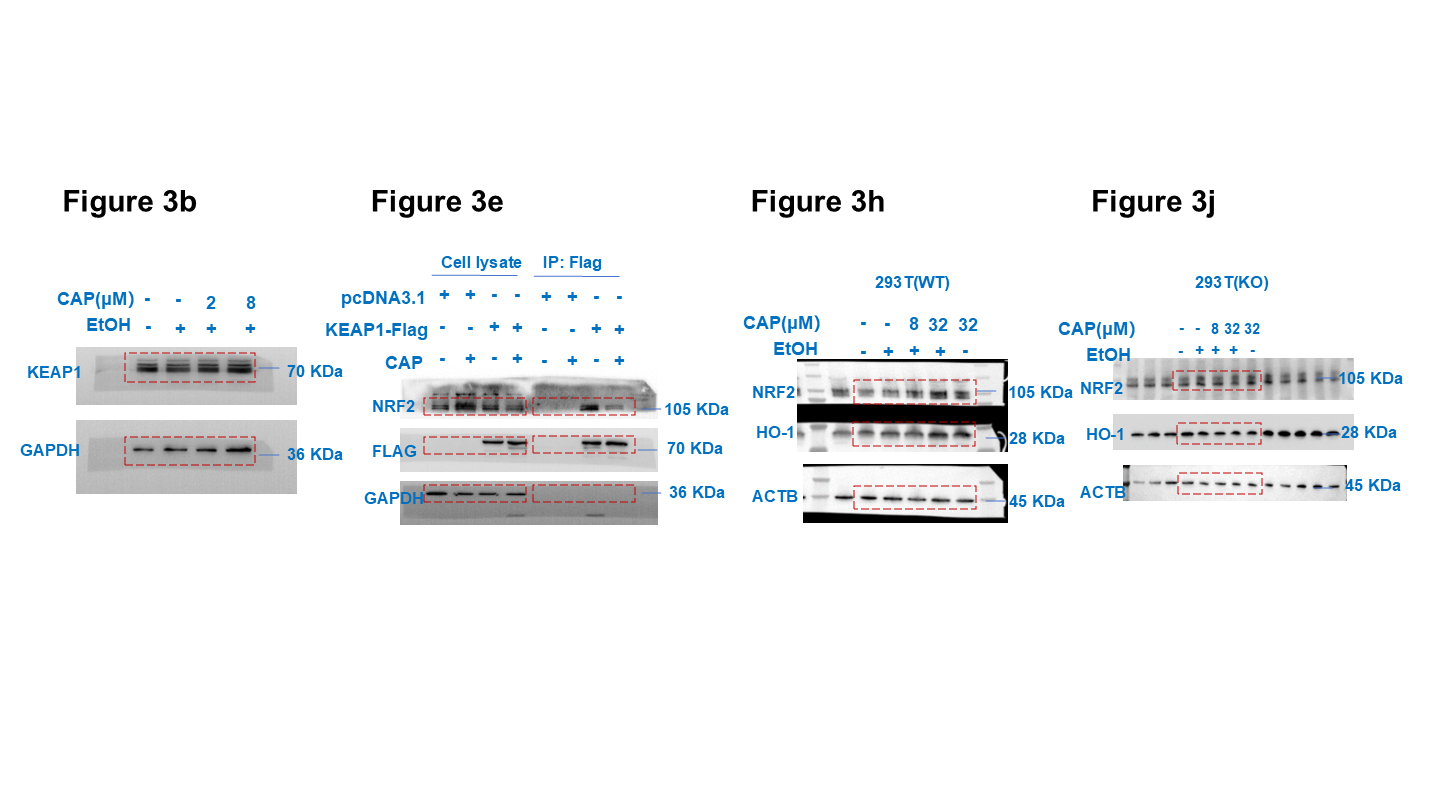

Supplement: Figure 3—source data 1. [file elife-97632-fig3-data1.zip › Figure 3-source data 1.tiff]

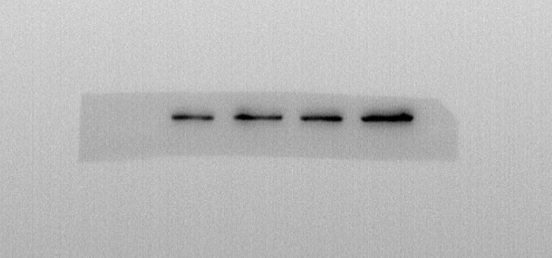

Supplement: Figure 3—source data 2. [file elife-97632-fig3-data2.zip › Figure 3-source data 2/Figure-3b GAPDH.tif]

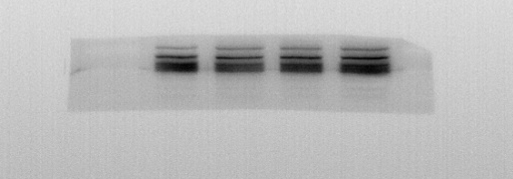

Supplement: Figure 3—source data 2. [file elife-97632-fig3-data2.zip › Figure 3-source data 2/Figure-3b KEAP1.tif]

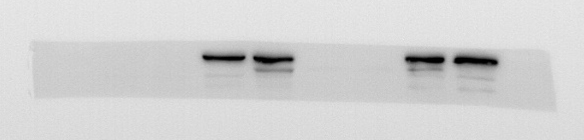

Supplement: Figure 3—source data 2. [file elife-97632-fig3-data2.zip › Figure 3-source data 2/Figure-3e FLAG.tif]

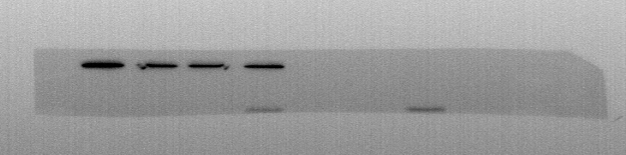

Supplement: Figure 3—source data 2. [file elife-97632-fig3-data2.zip › Figure 3-source data 2/Figure-3e GAPDH.tif]

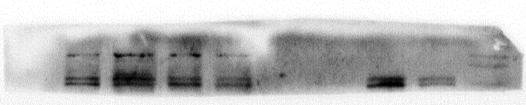

Supplement: Figure 3—source data 2. [file elife-97632-fig3-data2.zip › Figure 3-source data 2/Figure-3e NRF2.tif]

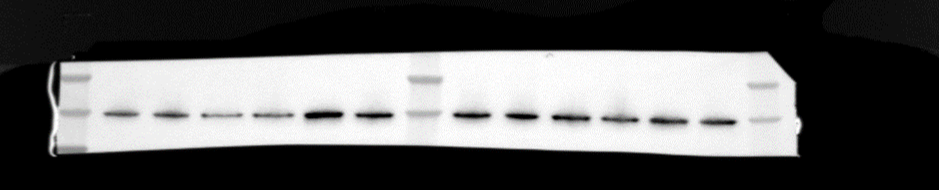

Supplement: Figure 3—source data 2. [file elife-97632-fig3-data2.zip › Figure 3-source data 2/Figure-3h ACTB.tif]

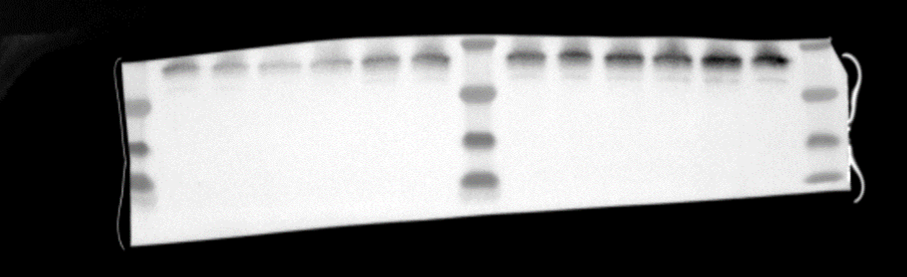

Supplement: Figure 3—source data 2. [file elife-97632-fig3-data2.zip › Figure 3-source data 2/Figure-3h HO-1.tif]

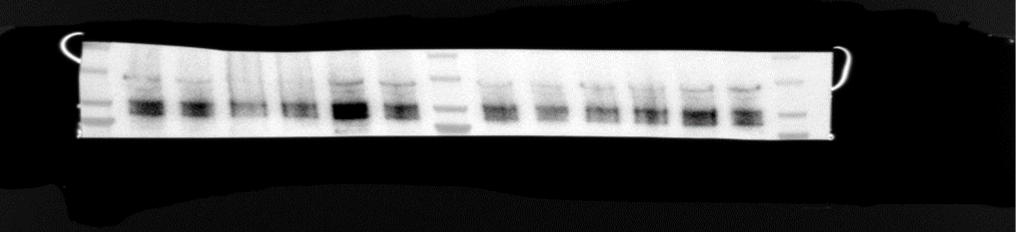

Supplement: Figure 3—source data 2. [file elife-97632-fig3-data2.zip › Figure 3-source data 2/Figure-3h NRF2.tif]

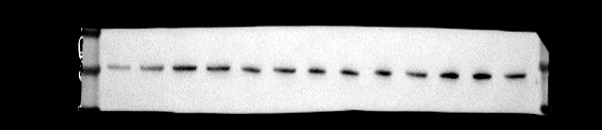

Supplement: Figure 3—source data 2. [file elife-97632-fig3-data2.zip › Figure 3-source data 2/Figure-3j ACTB.tif]

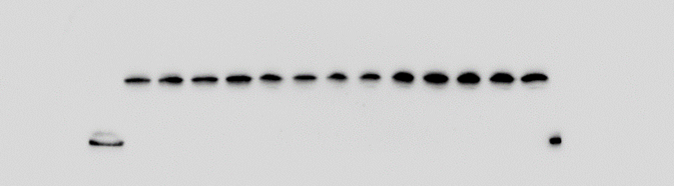

Supplement: Figure 3—source data 2. [file elife-97632-fig3-data2.zip › Figure 3-source data 2/Figure-3j HO-1.tif]

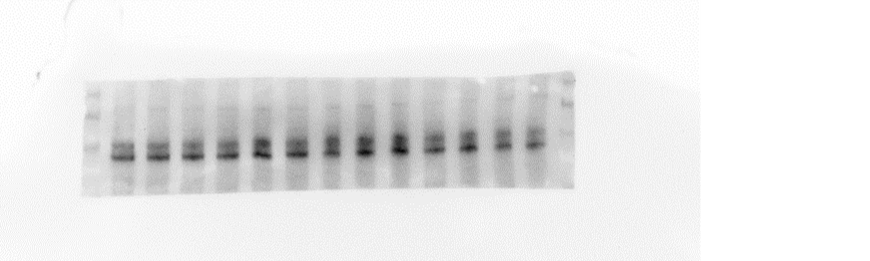

Supplement: Figure 3—source data 2. [file elife-97632-fig3-data2.zip › Figure 3-source data 2/Figure-3j NRF2.tif]

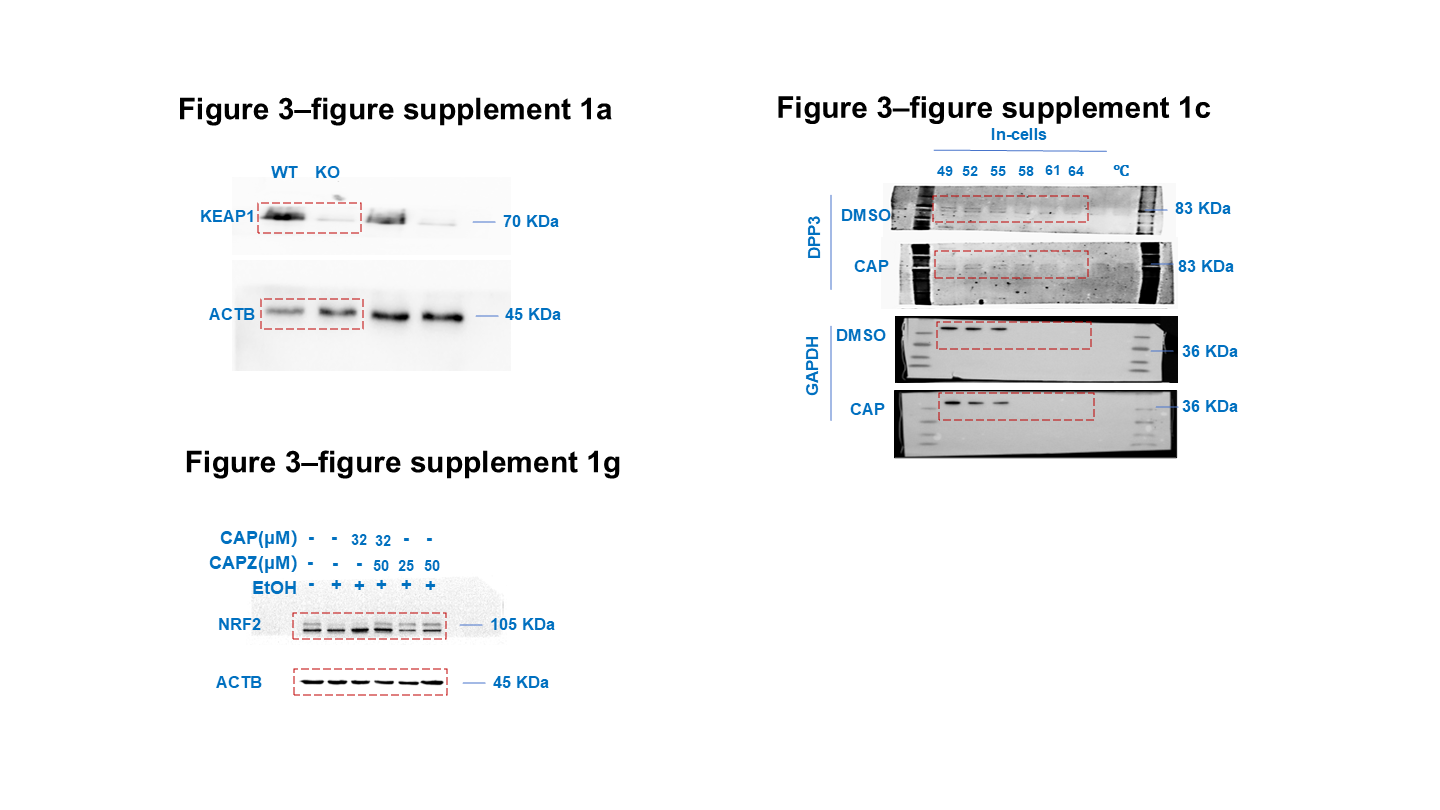

Supplement: Figure 3—figure supplement 1—source data 1. [file elife-97632-fig3-figsupp1-data1.zip › Figure 3-figure supplement 1-source data 1.tiff]

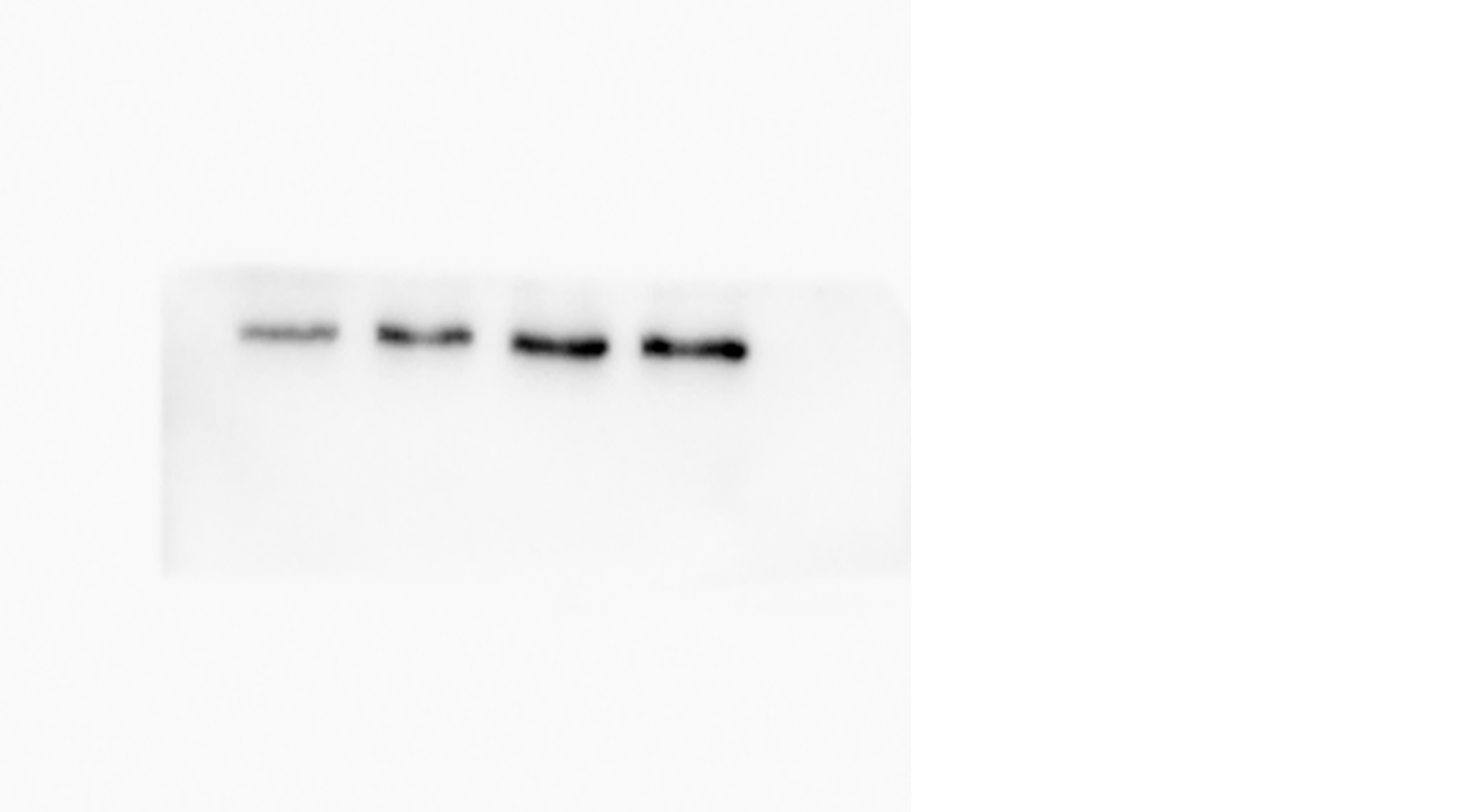

Supplement: Figure 3—figure supplement 1—source data 2. [file elife-97632-fig3-figsupp1-data2.zip › Figure 3-figure supplement 1-source data 2/Figure3-figure supplement 1a ACTB.tif]

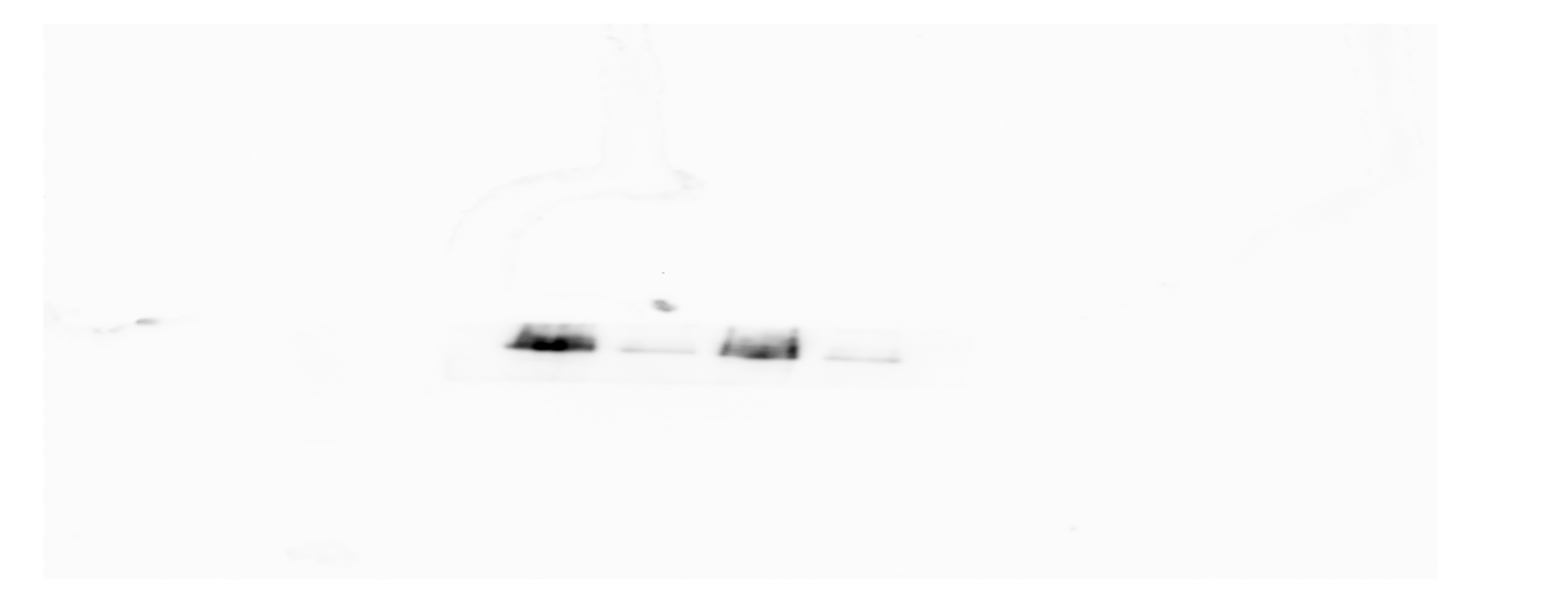

Supplement: Figure 3—figure supplement 1—source data 2. [file elife-97632-fig3-figsupp1-data2.zip › Figure 3-figure supplement 1-source data 2/Figure3-figure supplement 1a KEAP1.tif]

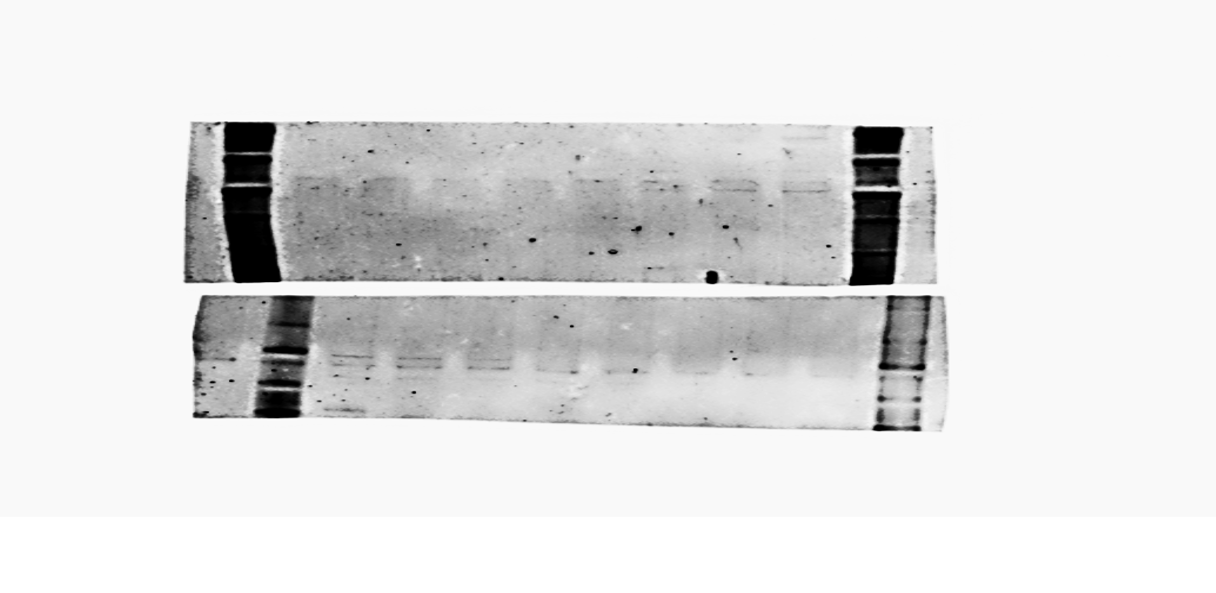

Supplement: Figure 3—figure supplement 1—source data 2. [file elife-97632-fig3-figsupp1-data2.zip › Figure 3-figure supplement 1-source data 2/Figure3-figure supplement 1c DPP3.tif]

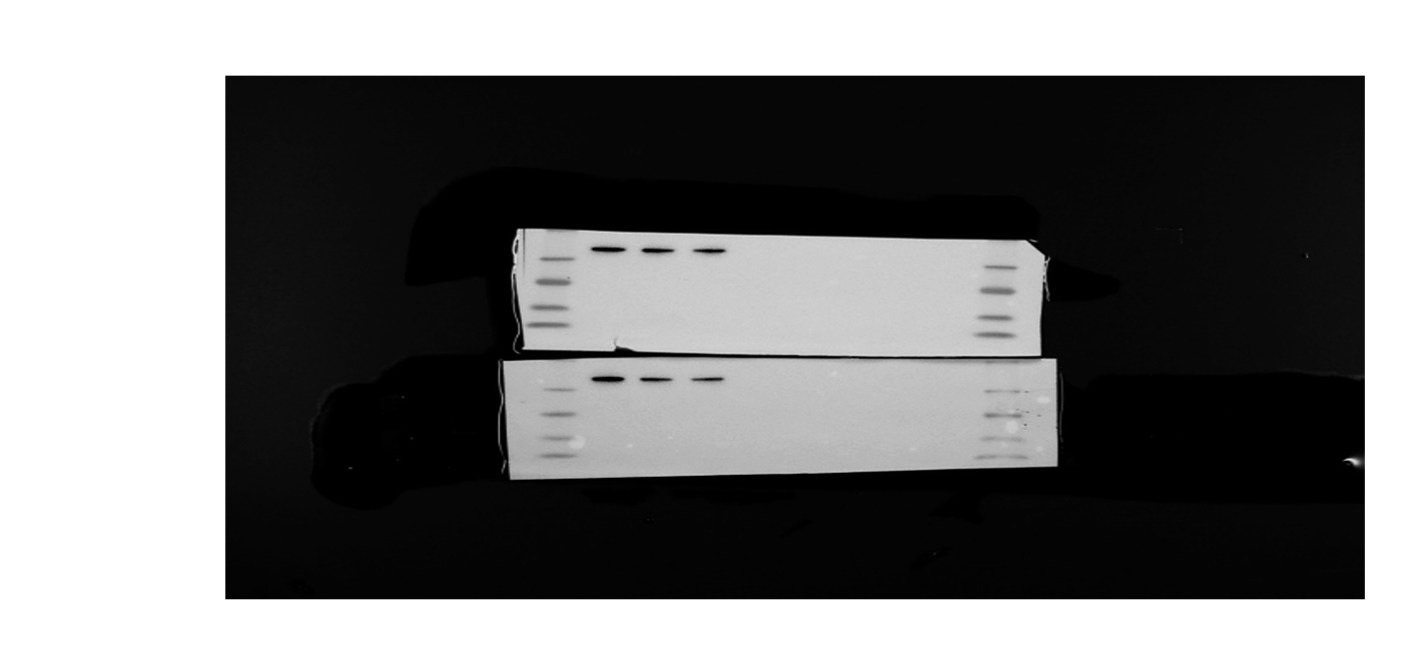

Supplement: Figure 3—figure supplement 1—source data 2. [file elife-97632-fig3-figsupp1-data2.zip › Figure 3-figure supplement 1-source data 2/Figure3-figure supplement 1c GAPDH.tif]

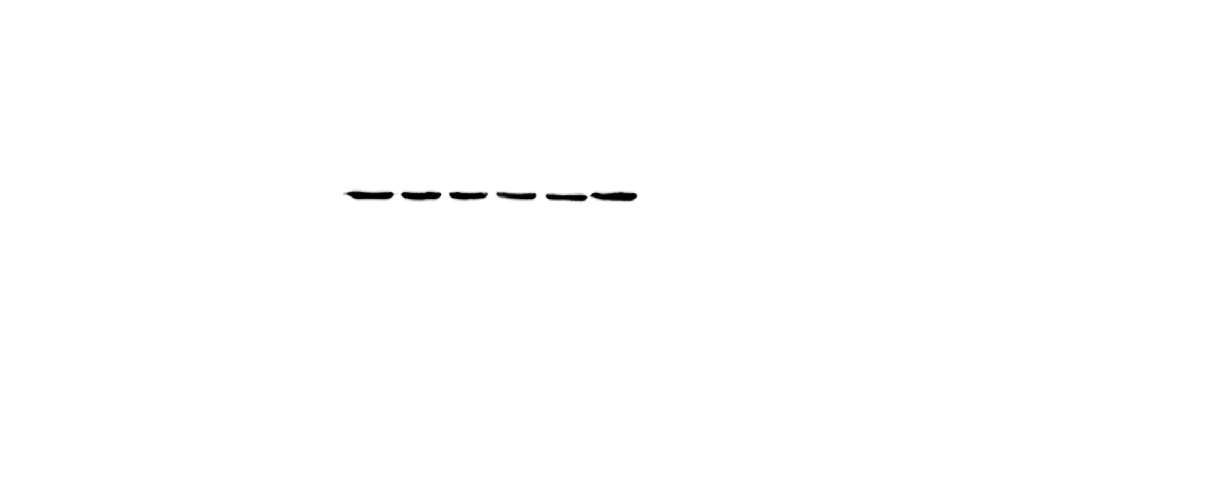

Supplement: Figure 3—figure supplement 1—source data 2. [file elife-97632-fig3-figsupp1-data2.zip › Figure 3-figure supplement 1-source data 2/Figure3-figure supplement 1g ACTB.tif]

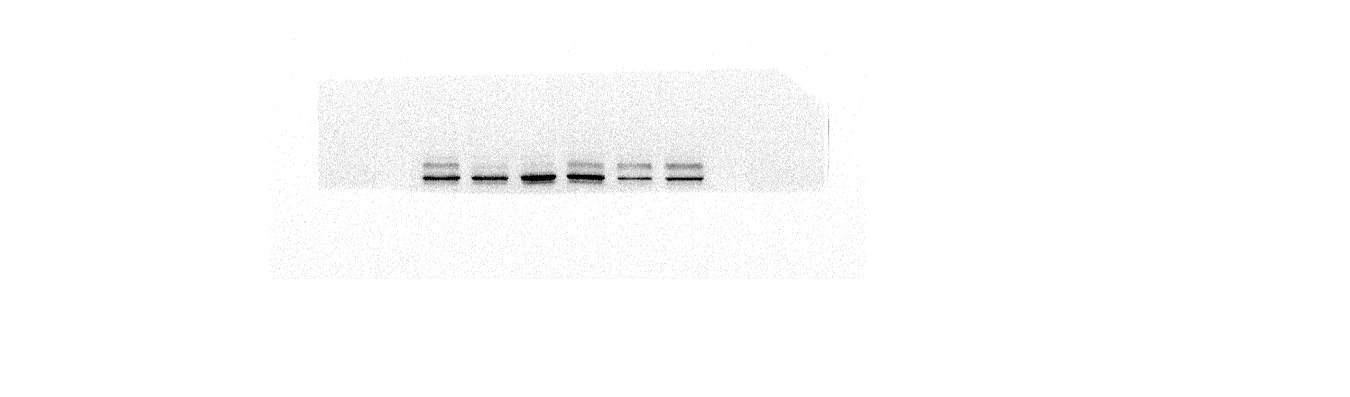

Supplement: Figure 3—figure supplement 1—source data 2. [file elife-97632-fig3-figsupp1-data2.zip › Figure 3-figure supplement 1-source data 2/Figure3-figure supplement 1g NRF2.tif]

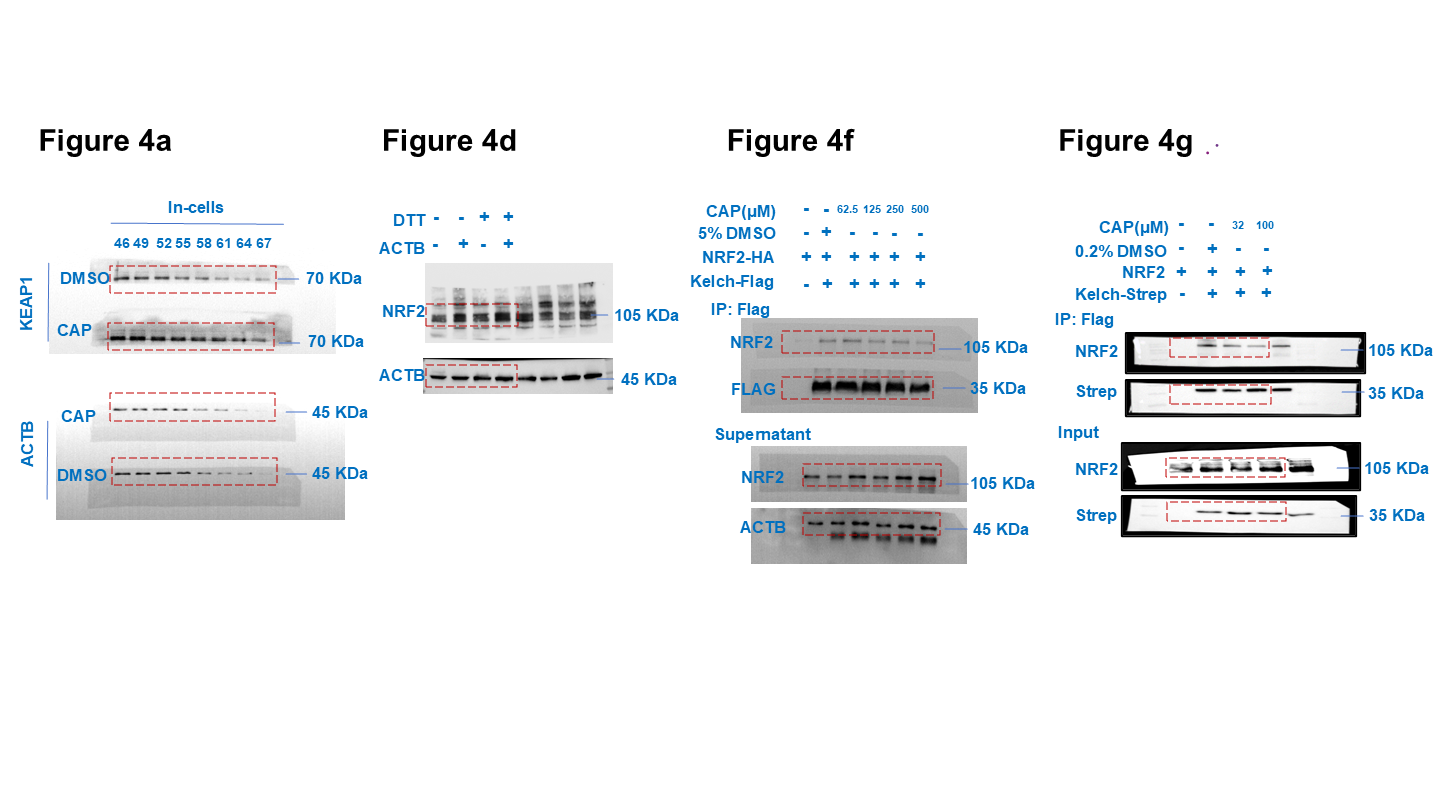

Supplement: Figure 4—source data 1. [file elife-97632-fig4-data1.zip › Figure 4-source data 1.tiff]

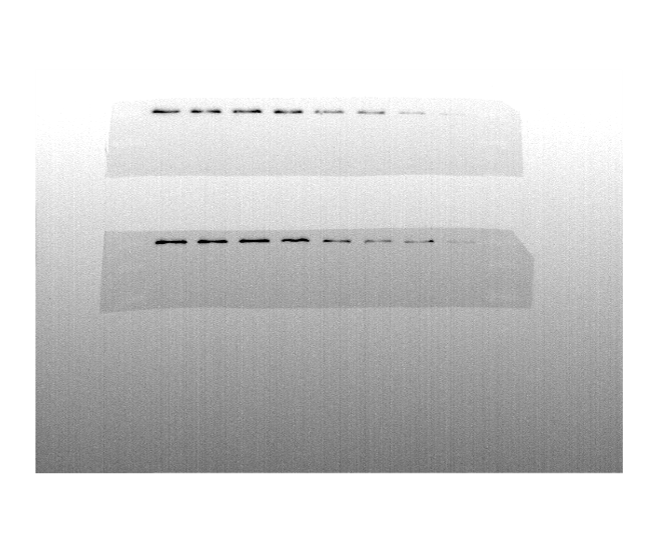

Supplement: Figure 4—source data 2. [file elife-97632-fig4-data2.zip › Figure 4-source data 2/Figure-4a ACTB.tif]

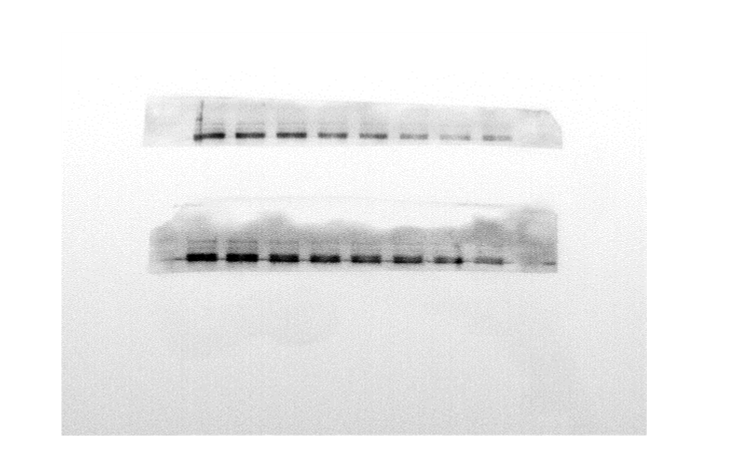

Supplement: Figure 4—source data 2. [file elife-97632-fig4-data2.zip › Figure 4-source data 2/Figure-4a KEAP1.tif]

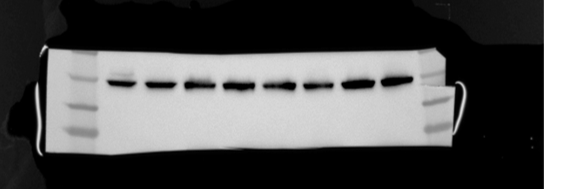

Supplement: Figure 4—source data 2. [file elife-97632-fig4-data2.zip › Figure 4-source data 2/Figure-4d ACTB.tif]

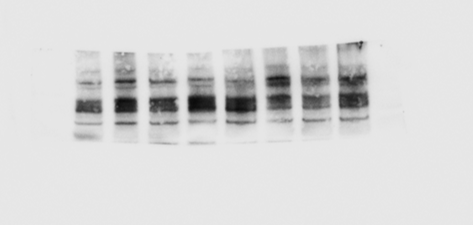

Supplement: Figure 4—source data 2. [file elife-97632-fig4-data2.zip › Figure 4-source data 2/Figure-4d NRF2.tif]

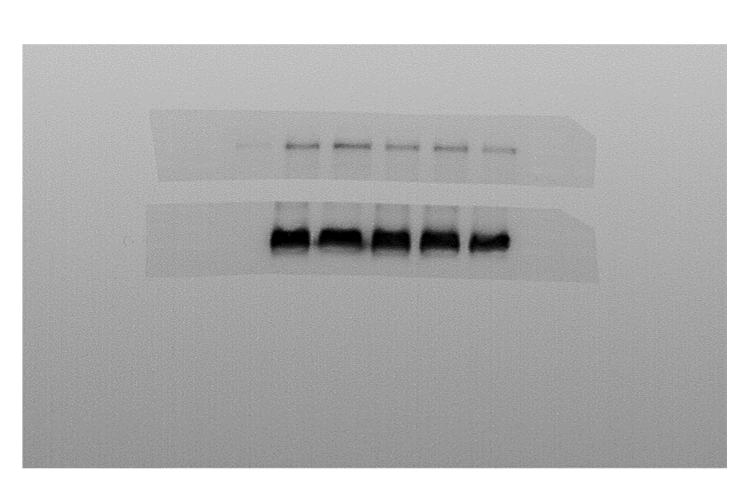

Supplement: Figure 4—source data 2. [file elife-97632-fig4-data2.zip › Figure 4-source data 2/Figure-4f IP NRF2 FLAG.tif]

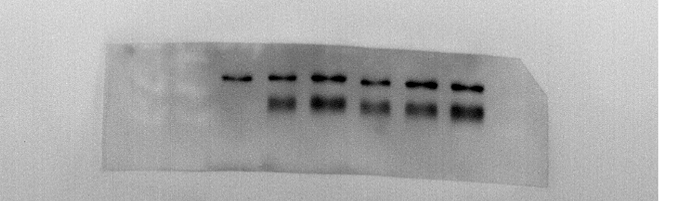

Supplement: Figure 4—source data 2. [file elife-97632-fig4-data2.zip › Figure 4-source data 2/Figure-4f Supernatant ACTB.tif]

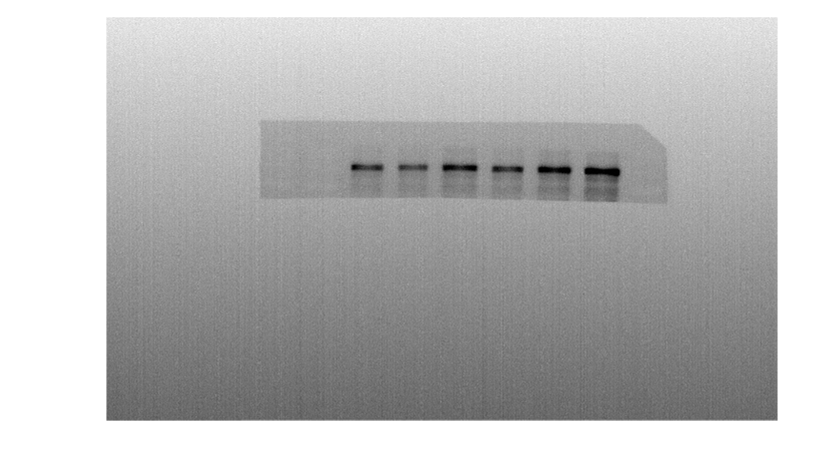

Supplement: Figure 4—source data 2. [file elife-97632-fig4-data2.zip › Figure 4-source data 2/Figure-4f Supernatant NRF2.tif]

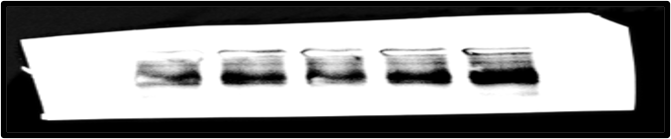

Supplement: Figure 4—source data 2. [file elife-97632-fig4-data2.zip › Figure 4-source data 2/Figure-4g Input NRF2.tif]

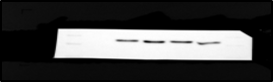

Supplement: Figure 4—source data 2. [file elife-97632-fig4-data2.zip › Figure 4-source data 2/Figure-4g Input Strep.tif]

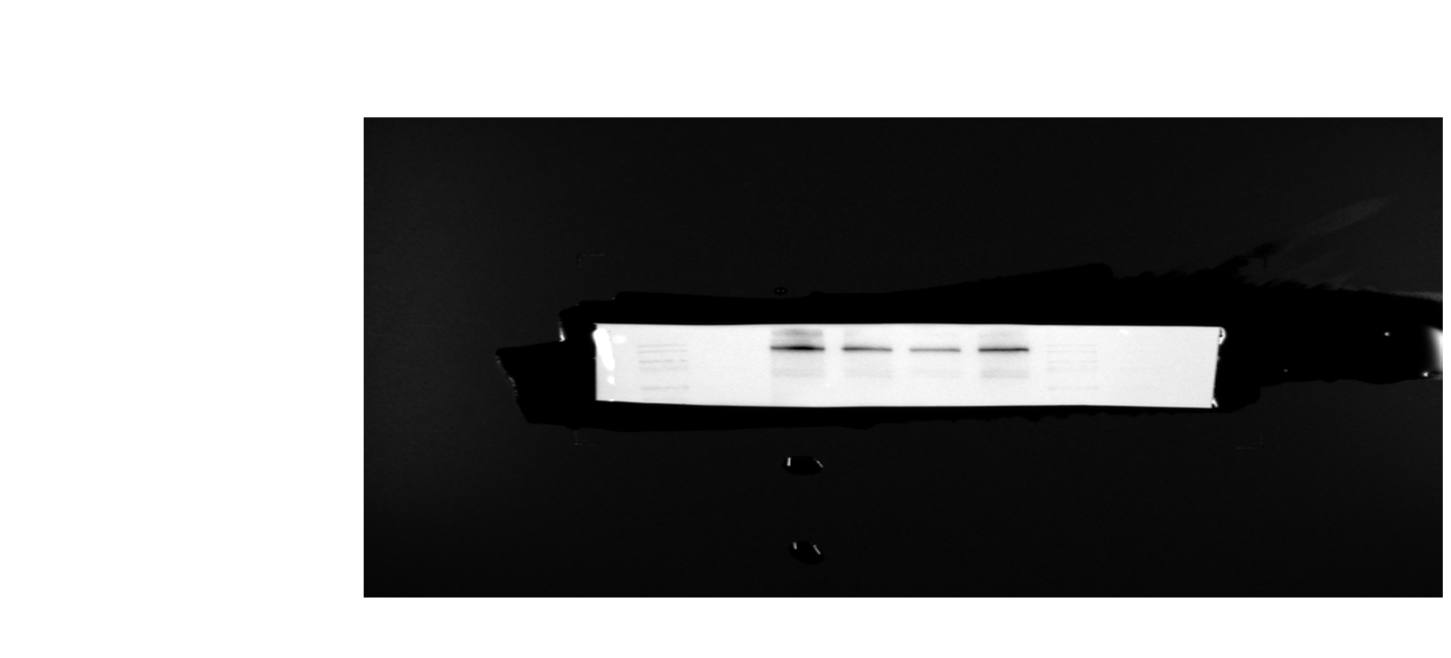

Supplement: Figure 4—source data 2. [file elife-97632-fig4-data2.zip › Figure 4-source data 2/Figure-4g IP NRF2.tif]

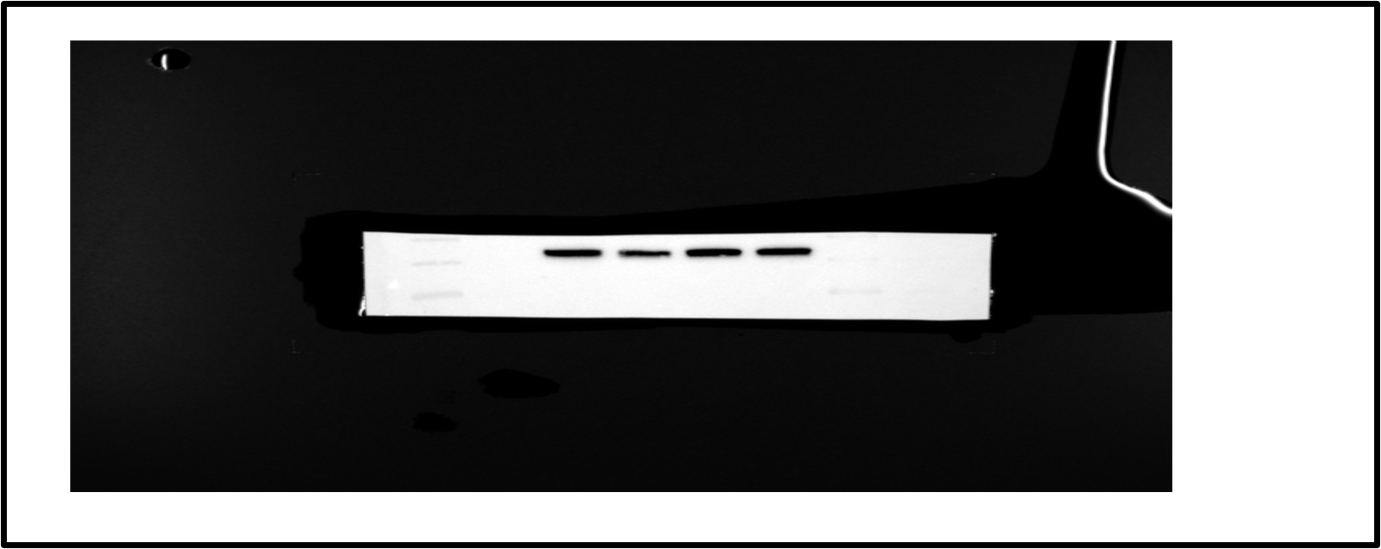

Supplement: Figure 4—source data 2. [file elife-97632-fig4-data2.zip › Figure 4-source data 2/Figure-4g IP Strep.tif]

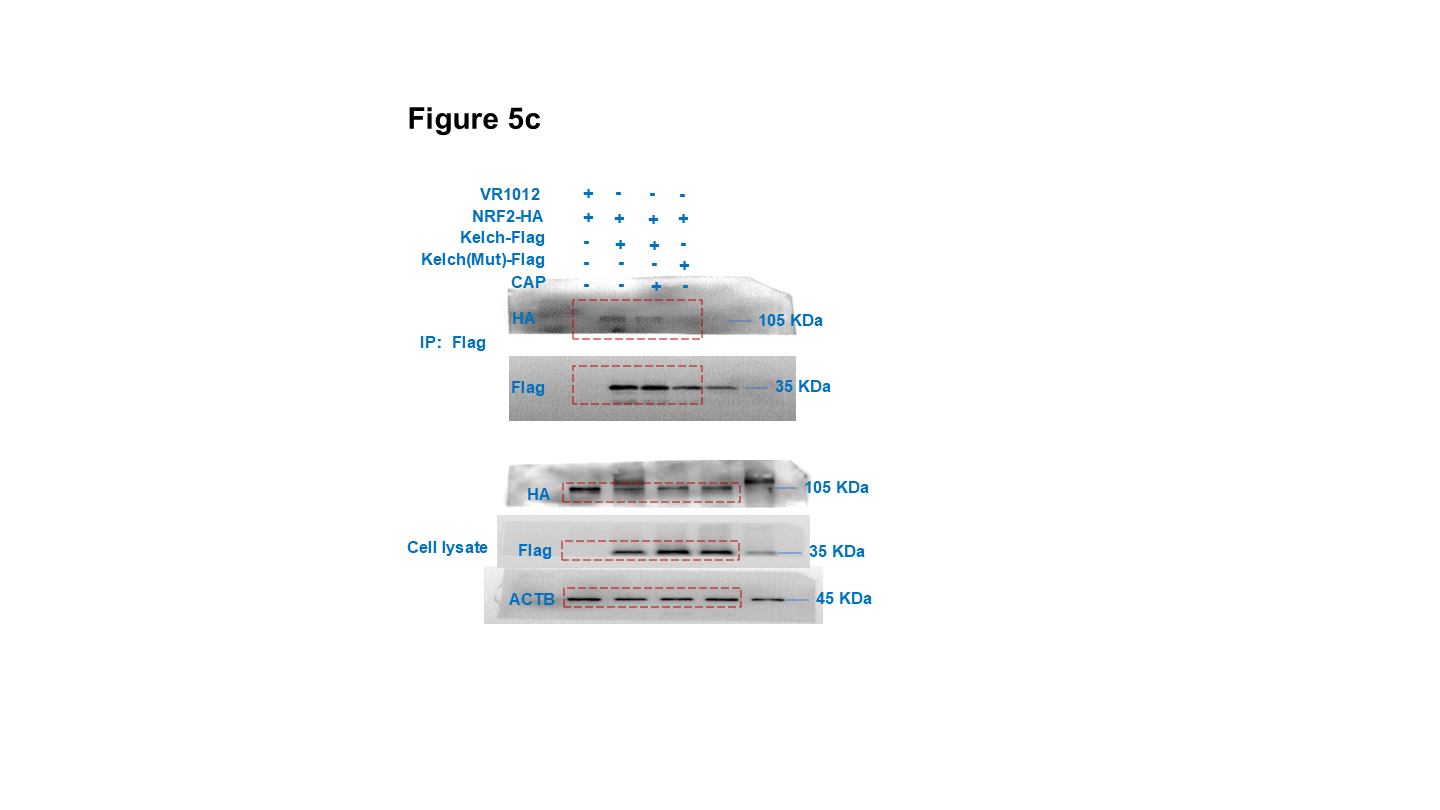

Supplement: Figure 5—source data 1. [file elife-97632-fig5-data1.zip › Figure 5-source data 1.tiff]

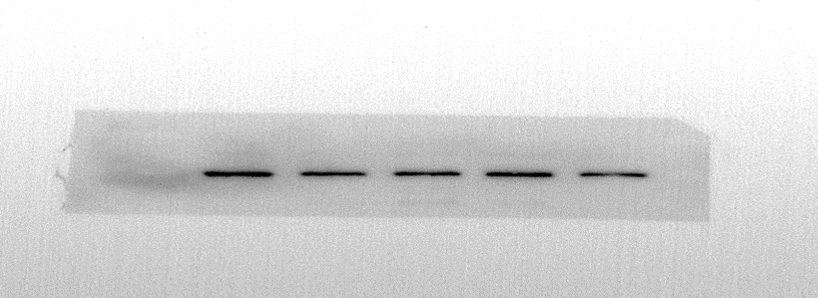

Supplement: Figure 5—source data 2. [file elife-97632-fig5-data2.zip › Figure 5-source data 2/Figure-5c Cell lysate ACTB.tif]

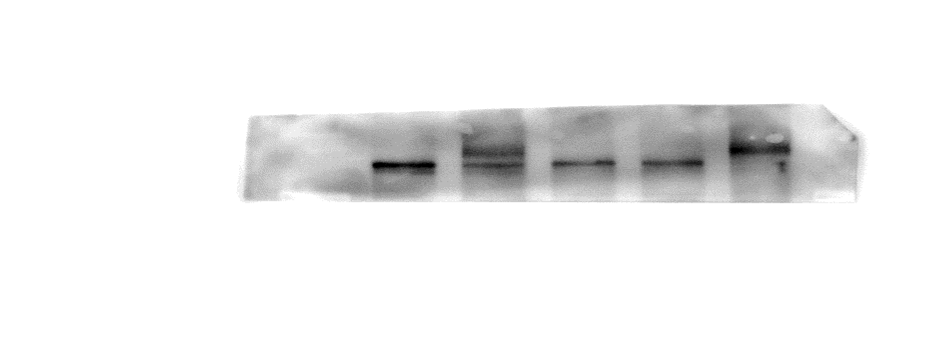

Supplement: Figure 5—source data 2. [file elife-97632-fig5-data2.zip › Figure 5-source data 2/Figure-5c Cell lysate Flag.tif]

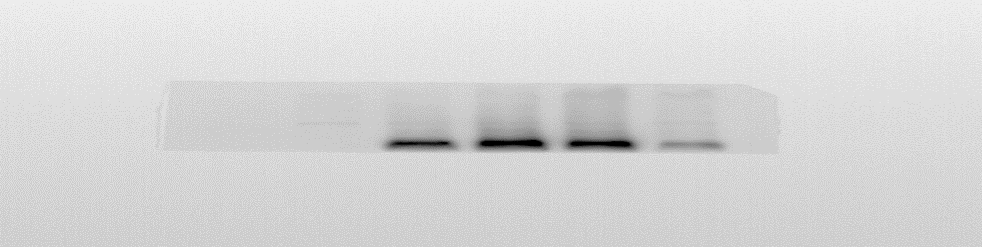

Supplement: Figure 5—source data 2. [file elife-97632-fig5-data2.zip › Figure 5-source data 2/Figure-5c Cell lysate HA.tif]

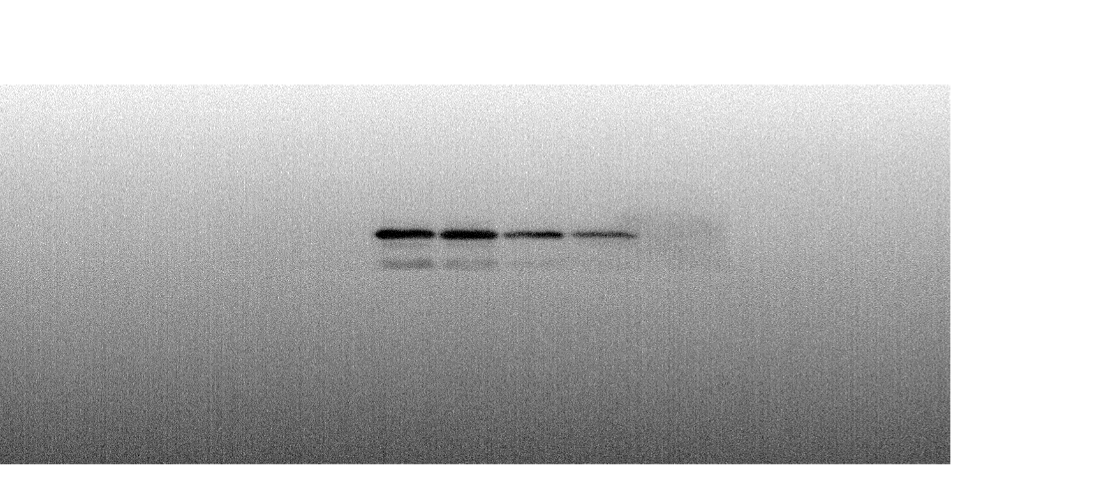

Supplement: Figure 5—source data 2. [file elife-97632-fig5-data2.zip › Figure 5-source data 2/Figure-5c IP Flag.tif]

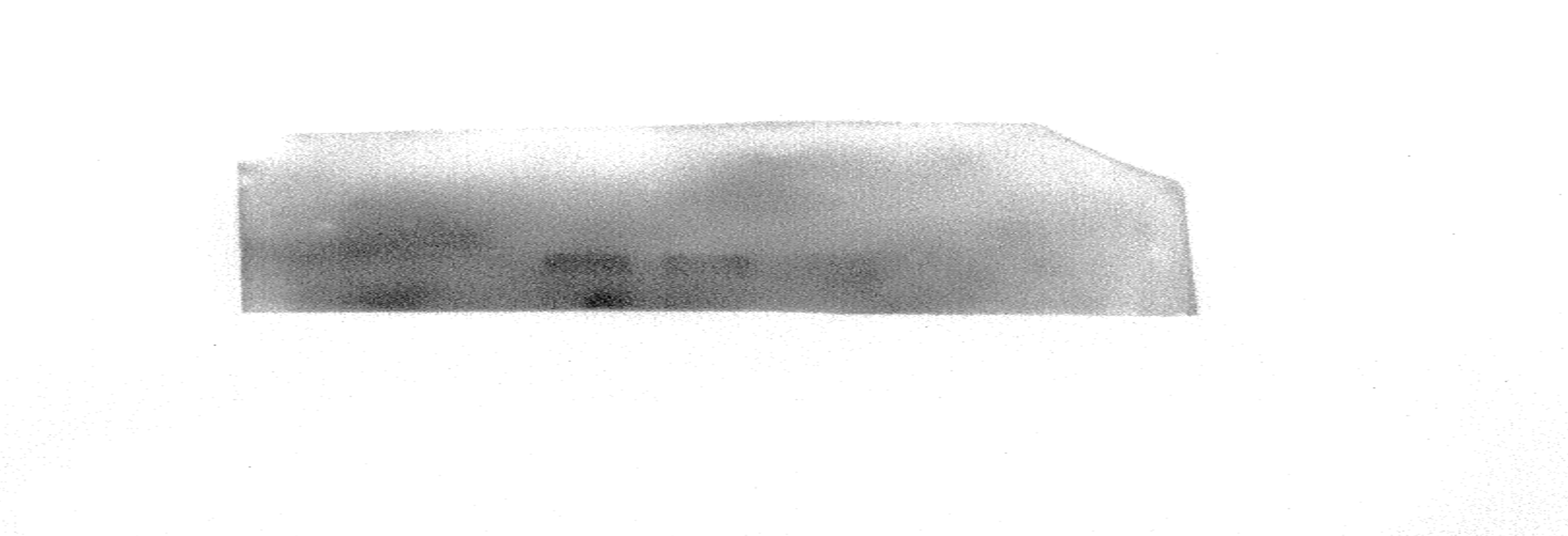

Supplement: Figure 5—source data 2. [file elife-97632-fig5-data2.zip › Figure 5-source data 2/Figure-5c IP HA.tif]

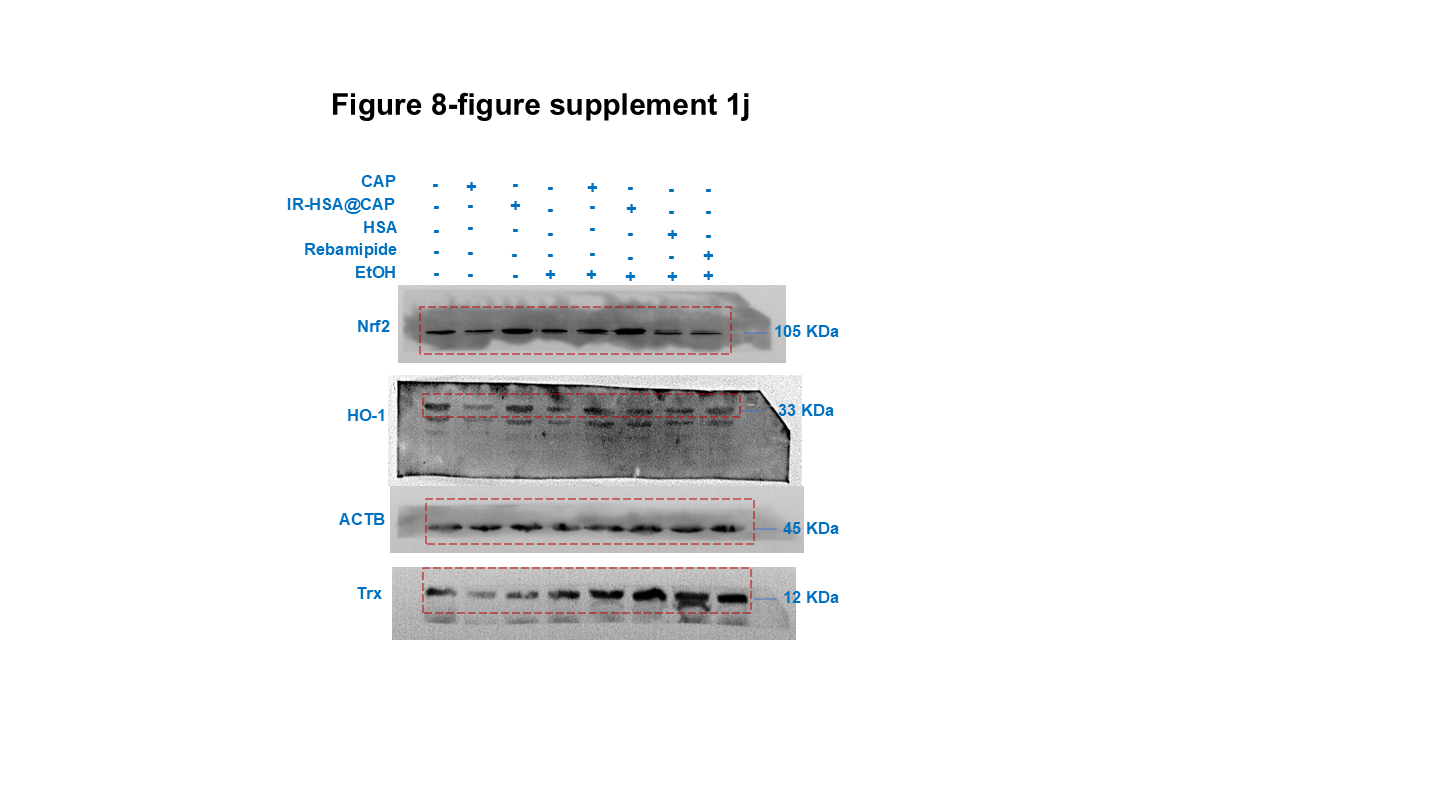

Supplement: Figure 8—figure supplement 1—source data 1. [file elife-97632-fig8-figsupp1-data1.zip › Figure 8-figure supplement 1-source data 1.tiff]

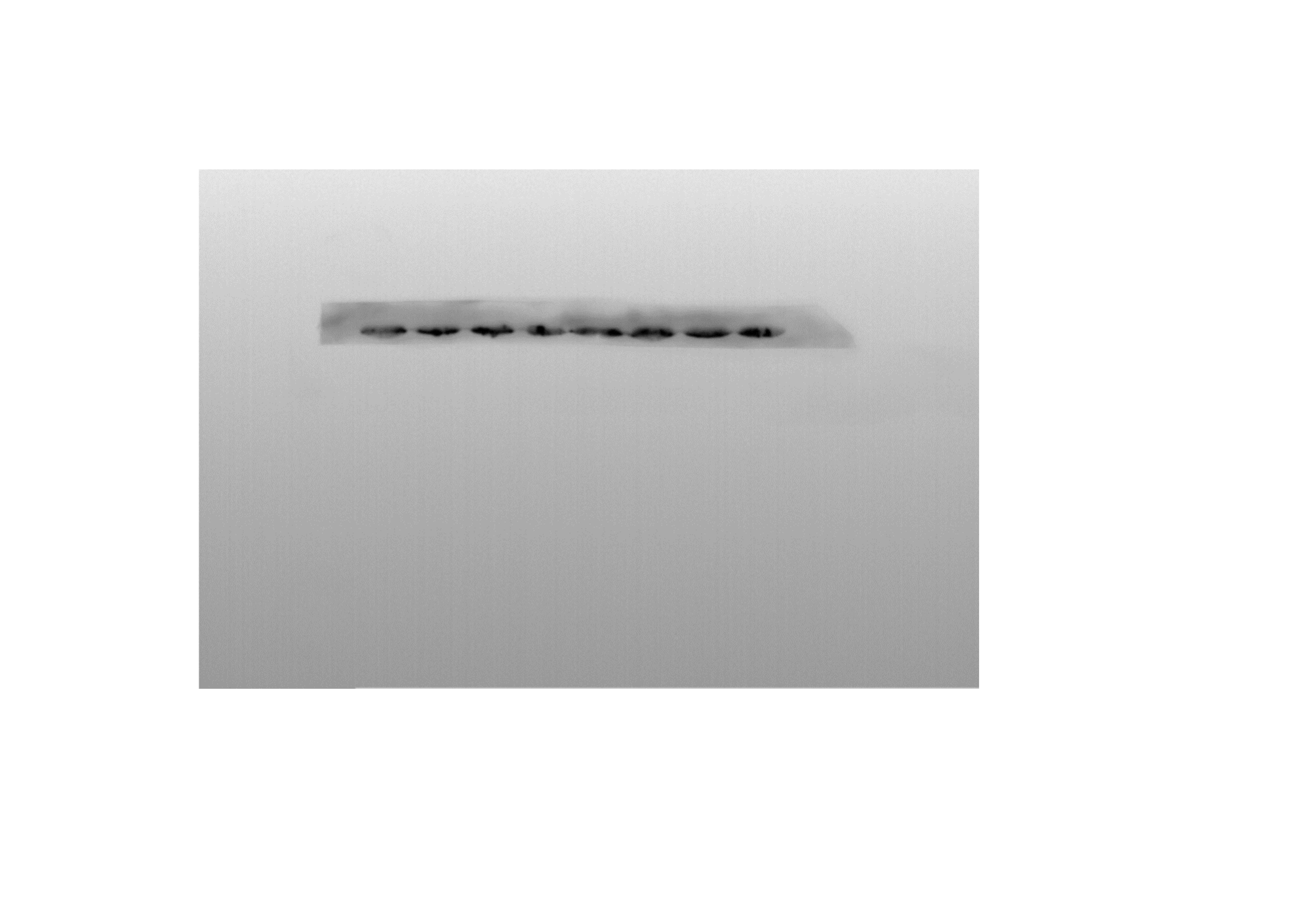

Supplement: Figure 8—figure supplement 1—source data 2. [file elife-97632-fig8-figsupp1-data2.zip › Figure 8-figure supplement 1-source data 2/Figure8-figure supplement 1j ACTB.tif]

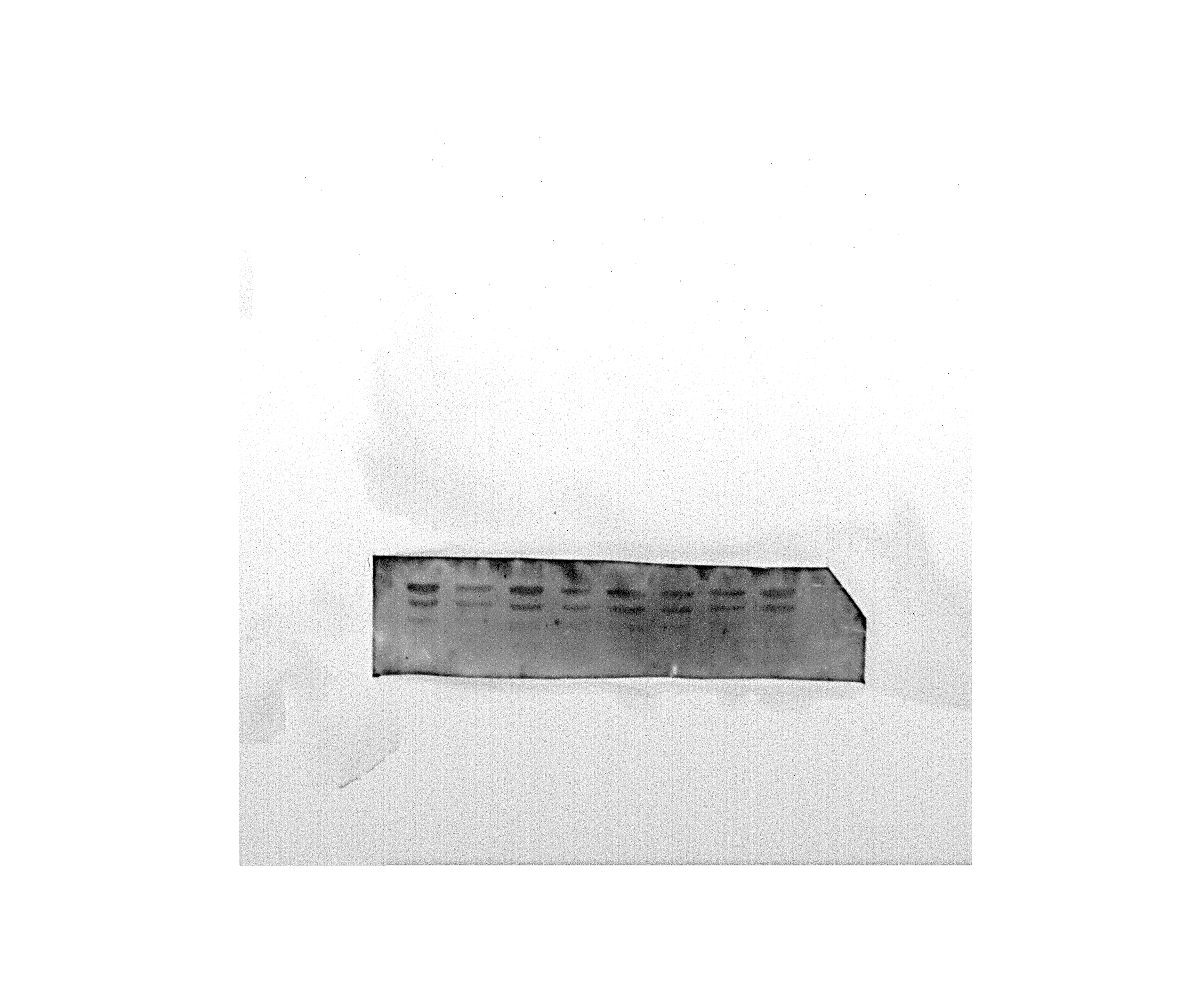

Supplement: Figure 8—figure supplement 1—source data 2. [file elife-97632-fig8-figsupp1-data2.zip › Figure 8-figure supplement 1-source data 2/Figure8-figure supplement 1j HO-1.tif]

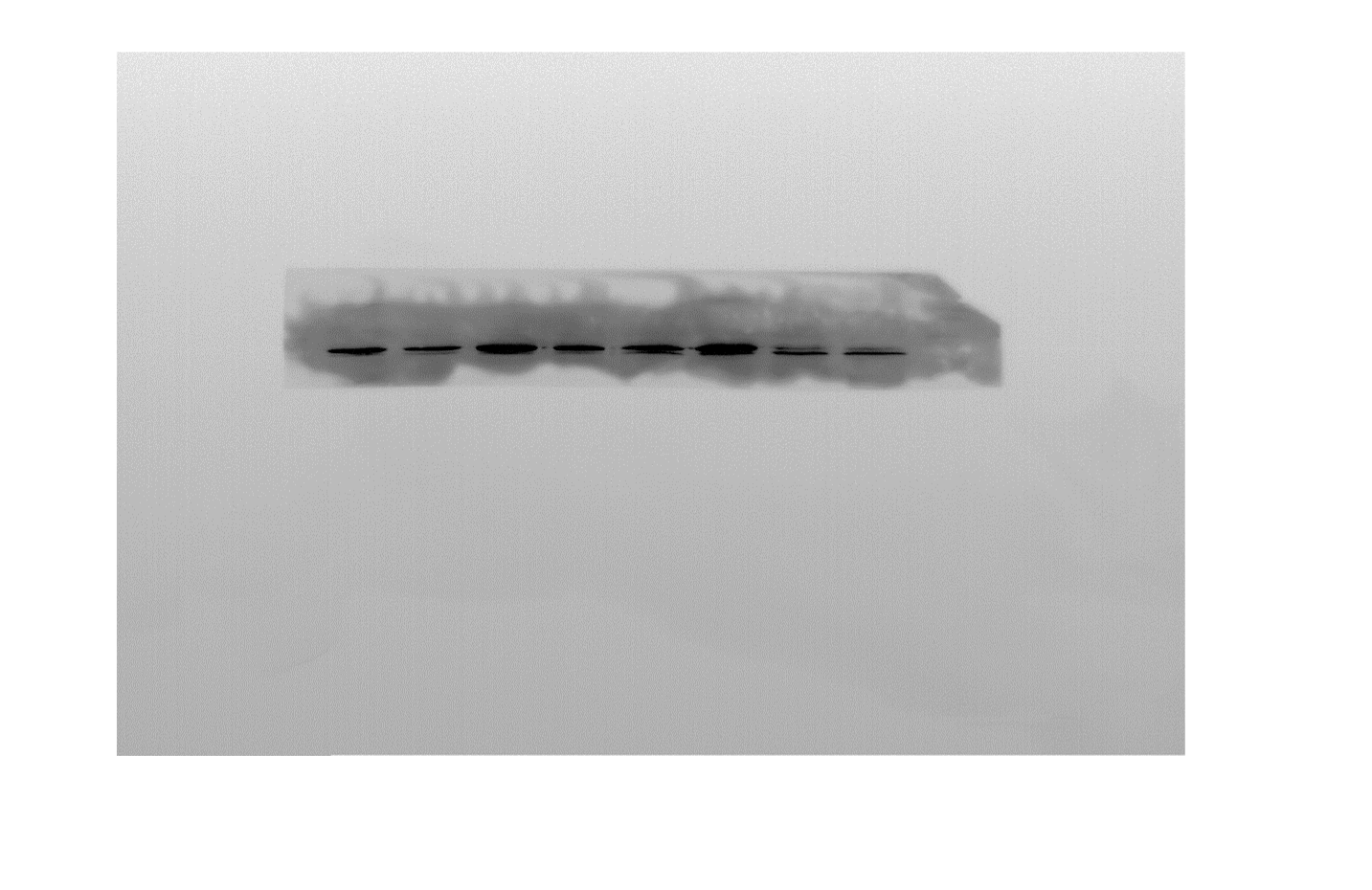

Supplement: Figure 8—figure supplement 1—source data 2. [file elife-97632-fig8-figsupp1-data2.zip › Figure 8-figure supplement 1-source data 2/Figure8-figure supplement 1j Nrf2.tif]

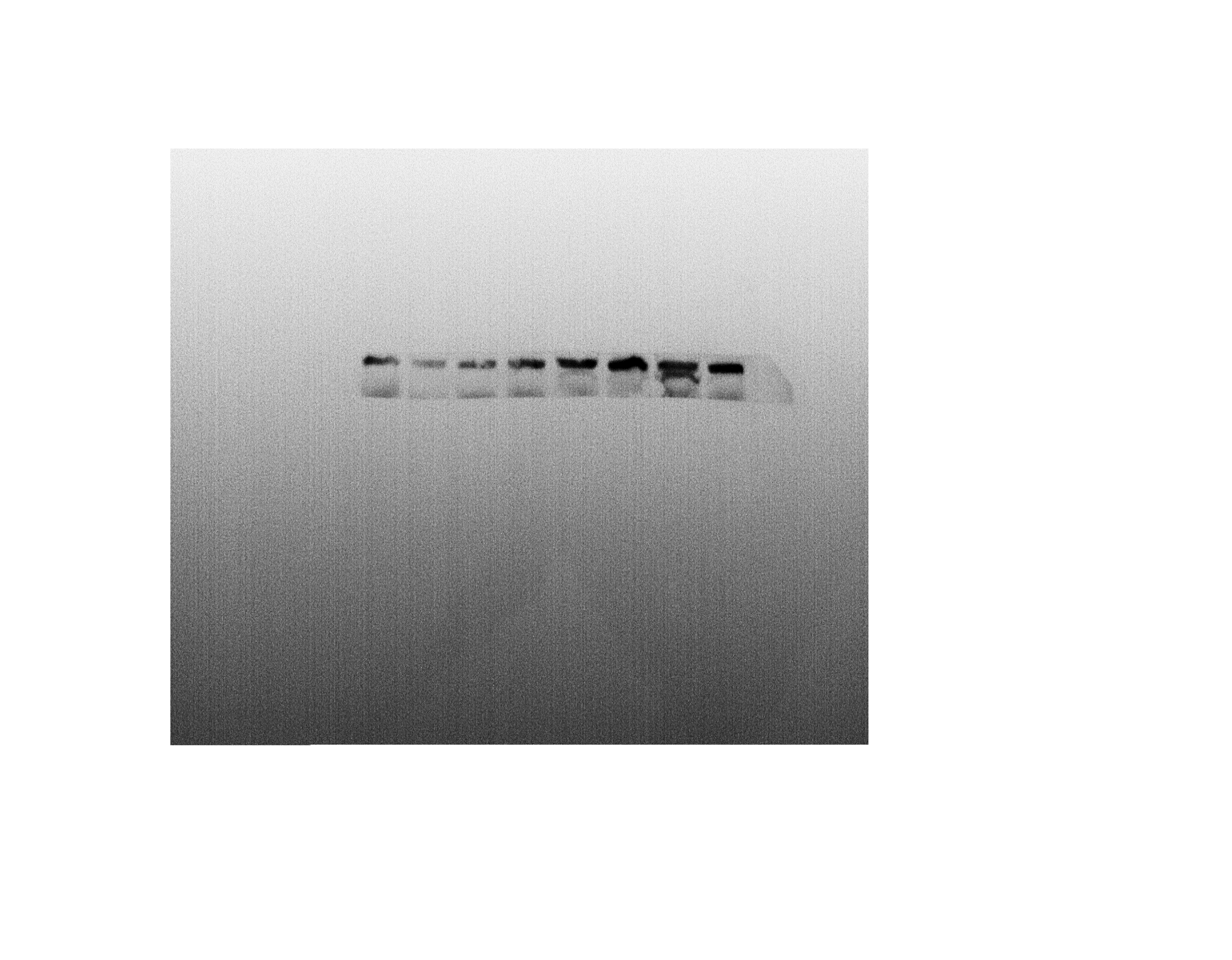

Supplement: Figure 8—figure supplement 1—source data 2. [file elife-97632-fig8-figsupp1-data2.zip › Figure 8-figure supplement 1-source data 2/Figure8-figure supplement 1j Trx.tif]

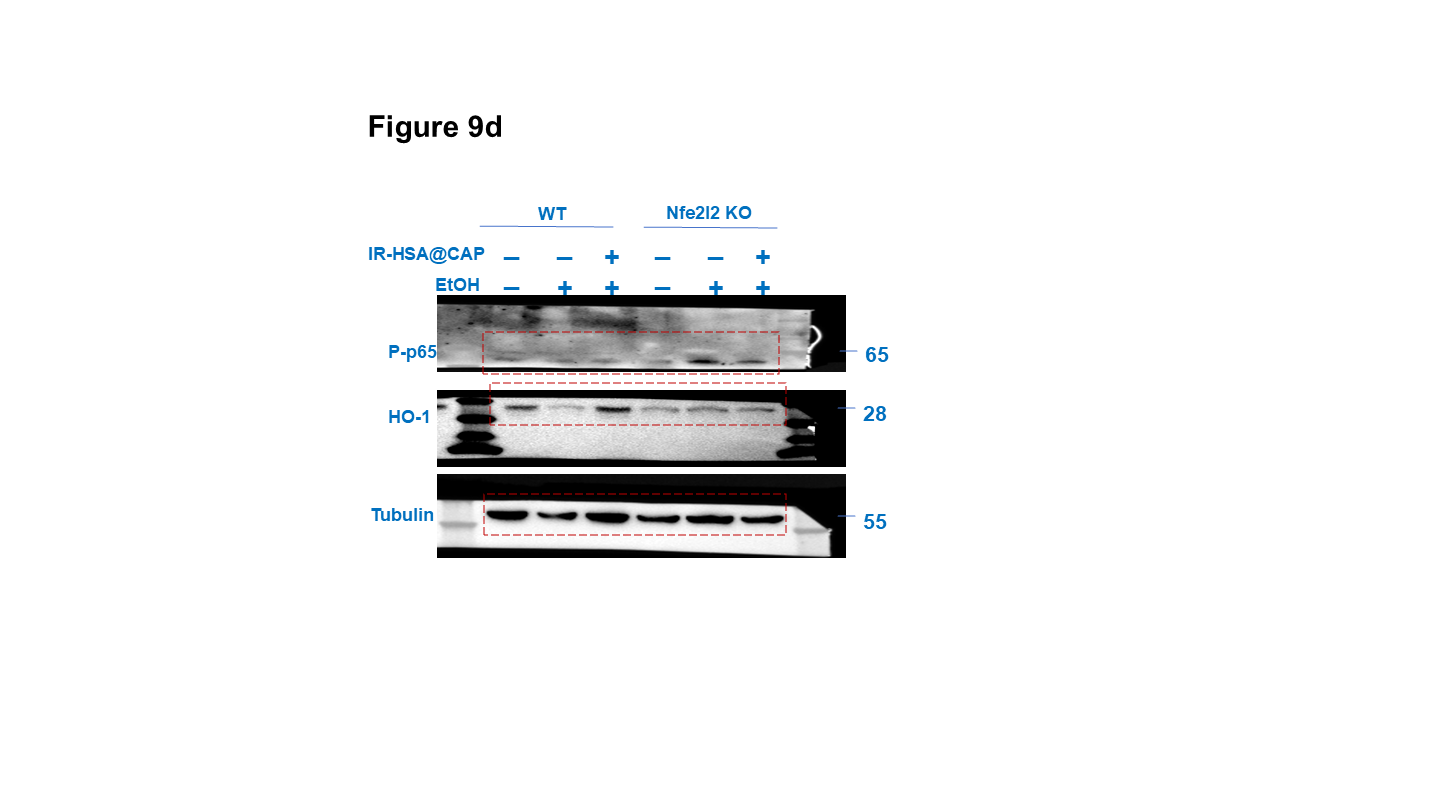

Supplement: Figure 9—source data 1. [file elife-97632-fig9-data1.zip › Figure 9-source data 1.tiff]

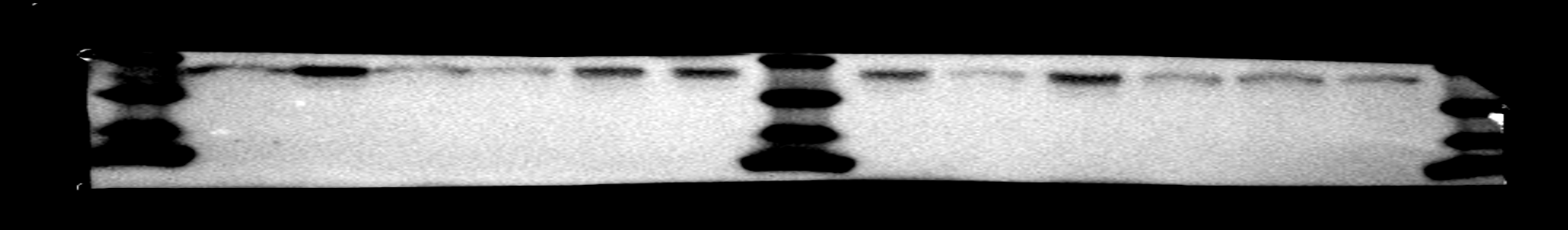

Supplement: Figure 9—source data 2. [file elife-97632-fig9-data2.zip › Figure 9-source data 2/Figure-9d HO-1.tif]

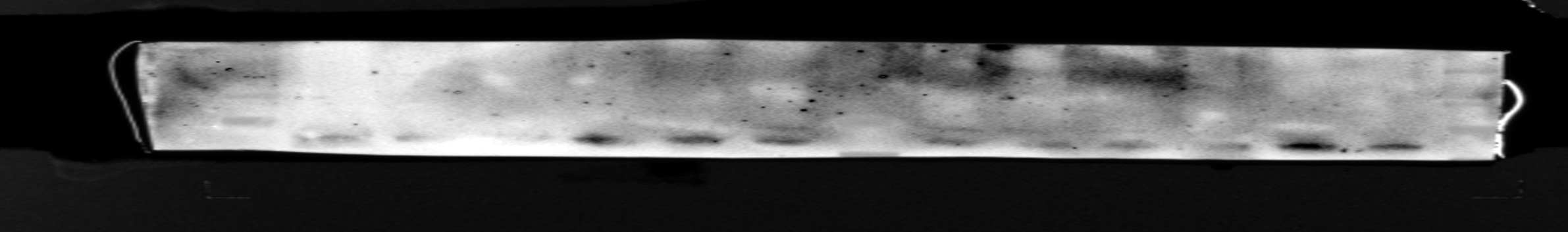

Supplement: Figure 9—source data 2. [file elife-97632-fig9-data2.zip › Figure 9-source data 2/Figure-9d P-p65.tif]

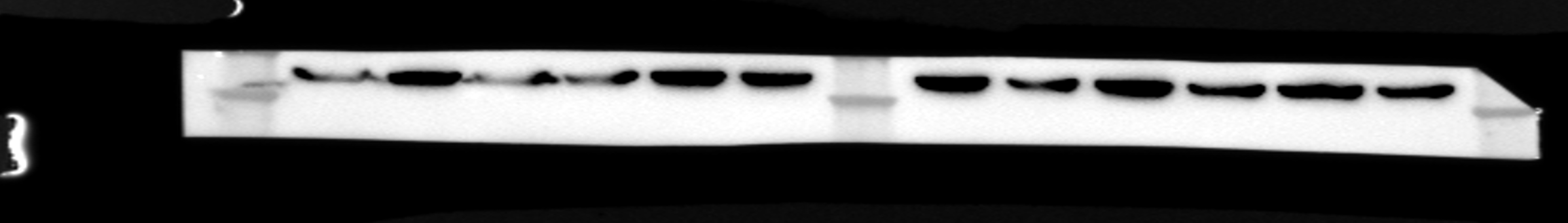

Supplement: Figure 9—source data 2. [file elife-97632-fig9-data2.zip › Figure 9-source data 2/Figure-9d Tubulin.tif]
