# Supplementary material for: Capsaicin acts as a novel NRF2 agonist to suppress ethanol induced gastric mucosa oxidative damage by directly disrupting the KEAP1-NRF2 interaction
Source: eLife. 2026 Jun 9;13:RP97632. doi: 10.7554/eLife.97632 (PMC13249423; doi:10.7554/eLife.97632)
Supplement: Peer review history

# Editor report: eLife Assessment

**DOI:** 10.7554/eLife.97632.4.sa0

**Authors:** Benoit Kornmann

**Affiliations:** University of Oxford United Kingdom

**Keywords:** Solid, Valuable

This **valuable** study suggests that capsaicin nanoparticle administration in rats activates the transcription factor Nrf2 by directly binding to its repressor, KEAP1, leading to the induction of cytoprotective genes and preventing alcohol-induced gastric damage, offering a potential avenue for treating alcoholism-related gastric disorders. The authors provide **solid** evidence through a wealth of biochemical experiments in vitro, in cultured cells as well as in a rat model. The work will be of great interest to researchers studying oxidative damage in a variety of different diseases and the exploitation of molecules for therapeutic approaches.

# Referee report: Reviewer #1 (Public review)

**DOI:** 10.7554/eLife.97632.4.sa1

The paper by Gao et al. describes the effect of capsaicin on the NRF2/KEAP1 pathway. The authors carried out a set of in vitro and in vivo experiments that addressed the mechanisms of the protective effect of capsaicin on ethanol-induced cytotoxicity.

The authors conclude that capsaicin activates NRF2, which leads to the induction of cytoprotective genes, preventing oxidative damage. The paper shows that capsaicin may directly bind to KEAP1 and that it is a noncovalent modification of the Kelch domain.

The authors also designed new albumin-coated capsaicin nanoparticles, which were tested for the therapeutic effect in vivo.

Comments on latest version:

The manuscript has been substantially improved. I have no further comments.

# Referee report: Reviewer #2 (Public review)

**DOI:** 10.7554/eLife.97632.4.sa2

Summary:

The paper by Gao et al. describes that capsaicin (CAP) might act as a novel NRF2 agonist capable of suppressing ethanol (EtOH)-induced oxidative damage in the gastric mucosa by disrupting the KEAP1-NRF2 interaction. Initially the authors established and validated a cell model for EtOH-induced oxidative stress which they used to experiment with different CAP concentrations and to determine changes in a variety of parameters such as cell morphology, ROS production, status of redox balance to mitochondrial function, amongst others.

The proposed mechanism by which CAP activates NRF2 and mitigates oxidative stress is thought to be via non-covalent binding to the Kelch-domain of KEAP1. A variety of assays such as BLI, CETSA, Pull-down, Co-IP, and HDX-MS were employed to investigate the KEAP1 binding behavior of CAP both in vitro and in GES1 cells. Consequently, the authors developed in vivo nanoparticles harboring CAP and tested those in a rat model. They found that pretreatment with the CAP-nanoparticles led to significant upregulation of NRF2 and subsequent effects on pro- (suppression of IL-1β, TNF-α, IL-6 and CXCL1) and anti-inflammatory (activation of IL-10) cyotkines pointing to a resolved state of inflammation and oxidative stress.

Strengths:

The work comprises a comprehensive approach with a variety of in vitro assays as well as cell culture experiments to investigate CAP binding behaviour to KEAP1. In addition, the authors employ in vivo validation by establishing an ethanol-induced acute gastric mucosal damage rat model and providing evidence of the potential therapeutic effect of CAP.

The study further provides novel insights into the mode of CAP action by elucidating the mechanism by which CAP promotes NRF2 expression and downstream antioxidant target gene activation.

The design of IR-Dye800 modified albumin-coated CAP nanoparticles for enhanced drug solubility and delivery efficiency demonstrates a valuable practical application of the research findings.

In summary the study's findings suggest that CAP could be a safe and novel NRF2 agonist with implications for the development of lead drugs for oxidative stress-related diseases. Collectively, the data support the significance and potential impact of CAP as a therapeutic agent for oxidative stress-related conditions.

Weaknesses:

While the study provides valuable insights into the molecular mechanisms and in vivo effects of CAP, further clinical studies are needed to validate its efficacy and safety in human subjects. The study primarily focuses on the acute effects of CAP on ethanol-induced gastric mucosa damage. Long-term studies are necessary to assess the sustained therapeutic effects and potential side effects of CAP treatment.

While the design of CAP nanoparticles is innovative, further research is needed to optimize the nanoparticle formulation for enhanced efficacy and targeted delivery to specific tissues.

Addressing these weaknesses through additional research and clinical trials can strengthen the validity and applicability of CAP as a therapeutic agent for oxidative stress-related conditions.

# Author comment: Author response

**DOI:** 10.7554/eLife.97632.4.sa3

**Authors:** Xiaoning Gao, Wuyan Guo, Peiyuan Liu, Mingyue Yuwen, Hongyu Ren, Shengtao Hu, Zixiang Liu, Ruyang Tan, Kairui Liu, Zhiru Yang, Junli Ba, Xue Bai, Shiti Shama, Cong Tang, Kai Miao, Haozhi Pei, Liren Liu, Cheng Zhu, Tao Wang, Bo Zhang, Jun Kang

**Affiliations:** Tianjin University Tianjin China; Tianjin JiAnKang Bio and TCM-technology Development Co., Ltd. Tianjin China; Tianjin University Tianjin China; Tianjin University Tianjin China; Tianjin University Tianjin China; Tianjin University Tianjin China; Tianjin University Tianjin China; Tianjin University Tianjin China; Tianjin University Tianjin China; Tianjin University Tianjin China; Tianjin University Tianjin China; Tianjin University Tianjin China; Tianjin University Tianjin China; Tianjin University Tianjin China; CAS and THHDG (TIANJIN) RURAL REVITALIZATION INDUSTRY DEVELOPMENT CO., LTD. Room 2803, Fujian Building, Chunhua Street, Hedong District, Tianjin, 300000, China Tianjin China; Tianjin University Tianjin China; Tianjin Medical University Cancer Institute and Hospital Tianjin China; Tianjin University Tianjin China; Tianjin University Tianjin China; Tsinghua University Beijing China; Tianjin University Tianjin China

The following is the authors’ response to the previous reviews

**Public Reviews:**

**Reviewer #1 (Public review):**

The paper by Gao et al. describes the effect of capsaicin on the NRF2/KEAP1 pathway. The authors carried out a set of in vitro and in vivo experiments that addressed the mechanisms of the protective effect of capsaicin on ethanol-induced cytotoxicity.

The authors conclude that capsaicin activates NRF2, which leads to the induction of cytoprotective genes, preventing oxidative damage. The paper shows that capsaicin may directly bind to KEAP1 and that it is a noncovalent modification of the Kelch domain.

The authors also designed new albumin-coated capsaicin nanoparticles, which were tested for.

I appreciate the authors' experimental efforts to strengthen the study's conclusions. However, in my opinion, the paper is still not fully technically sound, which weakens the strength of the evidence.

Thank you very much for your constructive review. We are truly gratified by your recognition of our key findings—that capsaicin activates NRF2 by disrupting the KEAP1–NRF2 interaction, as conclusively demonstrated through multiple methods including Pull-down, Co-IP, CETSA, SPR, BLI, deuterium exchange MS, CETSA, MS simulations and other target gene expression assays, and that albumin-coated capsaicin nanoparticles exhibit therapeutic effects in vivo. Your technical suggestions were particularly valuable. In this revised version, We have carefully and thoroughly addressed the points raised by you and the other reviewer by providing additional data, including nuclear-cytoplasmic fractionation assays performed with an alternative NRF2 antibody to strengthen and clarify the supporting evidence. We believe this revision have significantly enhanced the overall quality and rigor of the manuscript. Regarding the limitation of the insufficient number of animals used in this article, we have also explained it in the main text. This is the revision we have made with our utmost efforts, and we hope it can meet your expectations to a certain extent.

**Reviewer #2 (Public review):**

Summary:

In this paper the authors wanted to show that capsaicin can disrupt the interaction between Keap1 and Nrf2 by directly binding to Keap1 at an allosteric site. The resulting stabilization of Nrf2 would protect CAP-treated gastric cells from alcohol- induced redox stress and damage as well as inflammation (both in vitro and in vivo)

Strengths:

One major strength of the study is the use of multiple methods (CoIP, SPR, BLI, deuterium exchange MS, CETSA, MS simulations, target gene expression) that consistently show for the first time that capsaicin can disrupt the Nrf2/Keap1 interaction at an allosteric site and lead to stabilization and nuclear translocation of Nrf2.

Moreover, efforts to show causal involvement of the Keap/Nrf2 axis for the made cellular observations as well as addressing potential off target effects of the polypharmacological CAP appreciated.

One point that still hampers a bit of full appreciation of the capsaicin effect in cells is that capsaicin is not investigated alone, but mostly in combination with alcohol only.

Moreover, the true add-on value of the developed nanoparticles remains obscure.

The partly relatively high levels of NRF2 in putatively unstressed cells question the validity of used models.

The rationale for switching between different CAP concentrations is unclear /not entirely convincing.

The language and introduction could be improved.

Overall, the authors are convinced that capsaicin (although weakly) can bind to Keap1 and releases Nrf2 from degradation, with relevance for biological settings. With this, the authors provide a significant finding with marked relevance for the redox/Nrf2 as well as natural products /hit discovery communities.

Thank you very much for your positive assessment of our work and for the constructive suggestions to make it better. We also believe that capsaicin (CAP) offers new insights into the activation of NRF2. In this revision, we have addressed the shortcomings with the following efforts:

(1) The inclusion of a capsaicin (CAP)-only treatment group—covering the same doses and time points as the ethanol co-treatment—revealed that CAP alone can directly inhibit the KEAP1–NRF2 interaction (Figure 3d,3e and Figure 4g), and promote the entry of NRF2 into the nucleus (Figure 2c), resulting in moderate NRF2 activation (Figure 3h and Figure 4d) after carefully revision. However, this effect was significantly enhanced in the presence of ethanol. We attribute the results to the ROS-enriched environment generated by ethanol. Given that KEAP1 is a sensor highly susceptible to oxidative modification, the combination of CAP's allosteric regulation and ethanol-induced oxidative stress promotes a more robust and persistent dissociation of the KEAP1–NRF2 complex. These findings align fully with the established model in which KEAP1–NRF2 dissociation is markedly facilitated under oxidative stress conditions.

(2) From a translational and industrial perspective, nanoparticle formulations offer improved palatability compared with CAP itself; based on firsthand experience, the nano formulation is more tolerable than CAP. When preparing pure CAP, the pungency often causes irritation, whereas HSA@CAP nanoparticles are milder and demonstrate superior safety in mice following oral gavage. Moreover, ELISA results indicate that HSA@CAP nanoparticles exhibit enhanced anti-inflammatory activity compared with CAP alone (Figure 8d). In light of these findings, we prefer to retain this part of the data.

(3) Your question is highly professional and well taken. In GES-1 (Fig. 1i) and UC-MSC (Fig. 1l), the expression of NRF2 was low in unstressed conditions, and the transcription and translation of its downstream targets indicate no functional activation, supporting the validity of our model. Accordingly, the control groups in some experiments were suboptimal. We repeated these experiments with additional biological replicates and used cells with early-passage; the discrepancies likely relate to high passage numbers and serum batch effects, but they do not affect our main conclusions. We have replaced the relevant data in the revised manuscript (Fig. 2c and Fig. 3h) and hope this addresses your concern.

(4) In GES-1 cells, 8 μM consistently yielded the optimal effect, and we therefore maintained this concentration in other experiments in the same cells. And for other experiments, we needed to co-transfect multiple plasmids. Transfection efficiency was poor in GES-1 cells, so we switched to the commonly used HEK-293T cell line. In 293T cells, 2 and 8 μM were suboptimal, so we ultimately used 32 μM (Figure 3h), consistent with other 293T experiments (Co-IP and Pull-down) that also used 32 μM. Therefore, 8 μM were insufficient in Fig. 2g as we repeated many times. This likely reflects cell line–specific differences and the experimental context in 293T cells, including transfection and overexpression of NRF2 and Ub-K48-Myc, which necessitated a relatively higher CAP concentration.

(5) Thank you very much for noting that the language and Introduction could be further improved. We have rechecked the manuscript for grammar and style and revised the Introduction with a more comprehensive, up-to-date description of the NRF2 pathway. The main changes include rewriting the third and forth paragraph of the Introduction, consolidating/removing irrelevant sections for greater clarity and concision. We hope these updates meet your expectations.

Figure 2C: It is still not clear why naïve (unstressed /untreated cells) already show rather high nuclear abundance of Nrf2 (shouldn´t Nrf2 be continuously tagged for degradation by Keap1)

Thank you for your constructive comments. In response to the concern raised, we repeated the nuclear–cytoplasmic fractionation experiments in early-passage GES‑1 cells and performed three independent replications using an alternative, widely recognized NRF2 antibody (Cell Signaling Technology, Cat. No. 12721). The results showed low nuclear NRF2 levels under basal conditions, consistent with the KEAP1-mediated continuous degradation mechanism. Accordingly, we have updated the relevant figure in Figure 2C. Nevertheless, NRF2 could still be detected in the control group, which is basically consistent with the reported baseline levels of NRF2 observed in GES - 1 cells and other cell lines [1,2,3]. Therefore, this does not indicate the failure of model construction.

References：

(1) Wang, R. et al. Costunolide ameliorates MNNG-induced chronic atrophic gastritis through inhibiting oxidative stress and DNA damage via activation of Nrf2. Phytomedicine 130, 155581, doi:10.1016/j.phymed.2024.155581 (2024).

(2) Li, Y. F. et al. Construction of Magnolol Nanoparticles for Alleviation of Ethanol-Induced Acute Gastric Injury. J Agric Food Chem 72, 7933-7942, doi:10.1021/acs.jafc.3c09902 (2024).

(3) Li, M., Wang, J., Xu, Z., Lin, Y. & Dong, J. Atraric acid attenuates chronic intermittent hypoxia-induced brain injury via AMPK-mediated Nrf2 and FoxO3a antioxidant pathway activation. Phytomedicine 148, 157261, doi:10.1016/j.phymed.2025.157261 (2025).

Figure 2G-H: Why switch to rather high concentrations?

To validate ubiquitin-mediated degradation in Figure 2G-H, we needed to co-transfect multiple plasmids. Transfection efficiency was poor in GES-1 cells, so we switched to the commonly used HEK-293T cell line. In 293T cells, 2 and 8 μM were suboptimal, so we ultimately used 32 μM, consistent with other 293T experiments (Co-IP and Pull-down) that also used 32 μM. These choices reflect intrinsic cell line properties and protein overexpression in 293T, but do not affect our investigation of capsaicin’s mechanism.

Figure 2I: in the pics of mitochondria the control mitochondria look way more punctuated (likely fissed) than the ones treated with EtOH or EtOH + CAP. Wouldn´t one expect that EtOH leads to mitochondrial fission and CAP can prevent it?

Thank you very much for your comments. We re-acquired and analyzed mitochondrial morphology by the Leica STELLARIS 5 Confocal Microscope Platform, which our school didn't have at that time. The earlier wide-field fluorescence images lacked sufficient magnification and resolution, which obscured details and may have caused confusion. In the revised manuscript, we have replaced them with confocal images showing EtOH-induced mitochondrial abnormalities, whereas CAP treatment restored the reticular network, as expected. We also added a CAP-only group, which shows no discernible effect on mitochondrial morphology.

Figure 3H: High basal Nrf2 levels in unstressed/untreated HEK WT cells, why?

Thank you for raising this concern. We repeated the experiments in HEK-293T (WT) cells in better condition, and validated the results using an alternative, widely recognized NRF2 antibody (Cell Signaling Technology, Cat. No. 12721). The data consistently show relatively low NRF2 expression under basal conditions, in line with the KEAP1-mediated continuous degradation mechanism. We have corrected the corresponding figures accordingly.

Figure 4a: Inclusion of an additional Keap1 binding protein (one with a ETGE motif) would have been desirable (to get information on specificity/risks of off-target (unwanted) effects of CAP).

Thank you for this valuable suggestion. We have added CETSA experiments for DPP3, which contains an ETGE motif. The results show that endogenous DPP3 expression was low in GES-1 cells and does not bind CAP in vitro that BLI experiments indicated the KD was above 1 mM in Supplementary Figure 4h and 4i, and thus CAP does not thermally stabilize DPP3 at the cellular level. Therefore, the risk of off-target effects via binding to ETGE-containing proteins like DPP3 appears minimal.

Figure 4D: Why is there no stabilization of Nrf2 by CAP in lane 2?

Thank you for raising this concern. We repeated the experiment in GES‑1 cells and performed three independent replicates using an alternative, widely recognized Nrf2 antibody (Cell Signaling Technology, Cat. No. 12721). The data show that CAP alone increases NRF2 expression to some extent. We have updated the corresponding figures accordingly in Figure 4D.

Figure 4f: 5% DMSO is a rather high solvent concentration, why so high (the solvent alone seems to have quite marked effects!)

Thank you for raising this concern. Our original figure legend was misleading and has been corrected. Only the highest CAP concentration (500 μM) contained 5% DMSO as the vehicle; the other CAP concentrations, prepared by serial dilution in complete medium, did not contain such high solvent levels (e.g., 65.5 μM CAP contained 0.625% DMSO). This experiment included transient overexpression of NRF2-HA as purified recombinant NRF2 protein is prohibitively expensive, 10 ug needs about 900 GBP from Abcam. We therefore conducted a preliminary assay by incubating purified Kelch-Flag protein with cell lysates overexpressing NRF2-HA and measured NRF2 levels in the supernatant and pellet in Figure 4f. Nevertheless, the conclusion that CAP disrupts the NRF2–KEAP1 interaction is better supported by SPR (Figure 3d), Co-IP (Figure 3e) and Pull-down (Figure 4g).

Figure 6/7: not expert enough to judge formulations and histology scores. However, the benefit of the encapsulated capsaicin does not become entirely clear to me, as CAP and IRHSA@CAP mostly do not significantly differ in their elicited response.

Thank you very much for the valuable suggestion. Although histopathology suggests only modest differences between the two treatments, the nanoparticle group showed markedly lower inflammatory cytokine levels than pure CAP: IL-1β, IL-6, TNF-α, and CXCL-1 were significantly reduced, while the anti-inflammatory cytokine IL-10 was significantly increased (Figure 8d). These changes are important for maintaining a healthy gastric environment and may better support digestive function in vivo. Accordingly, from a translational and industrial perspective, nanoparticle formulations also offer improved palatability compared with capsaicin itself. Based on firsthand experience, the nano formulation is more tolerable than CAP. When preparing pure CAP, the pungency often causes irritation, whereas HSA@CAP nanoparticles are milder and demonstrate superior safety in mice following oral gavage.

Figure 7: Rebamipide was introduced as positive control in the text with an activating effect on Nrf2, but there is no induction of hmox and nqo in Figure 7f, why? It does not look as the positive control was wisely chosen.

Thank you for your insightful comment. We agree that this result was suboptimal and sincerely apologize for the oversight. We are currently facing significant constraints: the original cDNA is depleted, and funding cuts have severely limited our resources for reagents and animal studies. A full repetition of the rat experiment at the original scale and quality is not feasible in the short term. To ensure the scientific rigor of the paper, we have made the difficult decision to remove Figure 7f. We believe this is necessary to base our conclusions on the most robust evidence. We apologize for any inconvenience and hope this solution is acceptable. We have revised the manuscript accordingly and appreciate your understanding.

**Recommendations for the authors:**

**Reviewer #1 (Recommendations for the authors):**

1) The authors did not provide data validating the NRF2 antibody for in vitro studies, particularly for IF data where there is no molecular mass indication for NRF2. The IF data suggest that NRF2 is primarily located in the cytoplasm under control conditions (Fig. 2A), whereas the WB data show a strong band in the nucleus (Fig. 2C). What is the reason for this inconsistency?

We sincerely appreciate your valuable comments. Previously, we used an NRF2 antibody (Cat. No. 16396-1-AP, Proteintech); the vendor’s data show that shRNA knockdown in HeLa cells markedly reduces NRF2 at the expected molecular weight and IF data in HepG2 cells show a trace amount of cytoplasmic localization in controls and clear nuclear translocation after MG-132 treatment, which indicates that this antibody can be used for immunofluorescence (IF) to indicate the subcellular localization of NRF2, and our experimental results are also in line with expectations in Figure 2A. In addition, to address the reviewer's concern, we purchased another NRF2 antibody (Cat. No. 12721, Cell Signaling Technology), which was also highly validated. In this version, we repeated nuclear-cytoplasmic fractionation experiments and other important experiments using this antibody. Together, these data confirm the low basal level of NRF2 in the absence of stress and also show that CAP could improve the expression of NRF2. We have corrected the Figure 2C so that the WB and IF results are now consistent. We wish to reiterate our deep appreciation for the professionalism and rigor of your review.

(2) Additionally, I could not find Supplementary Figure 4F-I, which concerns TRPV1. These figures are mentioned in the response to reviewers but are missing from the manuscript-please double-check.

The supplementary figures were initially submitted as a compressed archive. We recognize that there might have been an issue with the transfer of this file to the reviewers. As shown in Supplement Figure 4f to Supplement Figure 4i, we further explored the TRPV1 and DPP3 to detect the potential off-target effects of CAP respectively. Capsazepine (CAPZ), which is TRPV1 receptor antagonist did not affect the protection of CAP against GES-1 (Fig S4f and S4g), which may indicate that CAP activation of NRF2 does not have to depend on TRPV1. The binding of CAP with DPP3, containing an ETGE motif and can bind to KEPA1, was detected by BLI, and we found that the KD between CAP and DPP3 was 1.653 mM(＞100 μM), which may indicate the potential off-target effect of CAP is low because CAP had a relatively strong binding force with KEAP1 about 31.45 μM (Fig S4h and S4i).

(3) I am also somewhat unconvinced by the data obtained from NRF2 KO mice. First, it appears that some NRF2 KO mice respond to CAP treatment well while others do not, resulting in a high standard deviation. To strengthen the conclusions, it would be advisable to use a larger number of animals to confirm or exclude the effect. This is precisely why I still believe that three rats per group are insufficient for the in vivo studies. Please emphasize in the manuscript that a limitation of this study is the use of only three rats per group for the in vivo experiments.

Thank you very much for your question and suggestions. As for the rat experiments in Figure 7 and Figure 8, there are many other references available, as noted in the introduction: “Recent experiments conducted in rats have demonstrated that red pepper/capsaicin (CAP) possesses significant protective effects on ethanol-induced gastric mucosal damage , and the mechanisms involved may relate to the promotion of vasodilation[6,7], increased mucus secretion[8] and the release of calcitonin gene-related peptide (CGRP)[9,10]. However, it is important to note that the specific role of the antioxidant activity of CAP has not been thoroughly investigated.” Therefore, we conducted extensive literature research and preliminary experiments to ensure that our formal experiment with 8 groups could yield relatively stable results. Of course, we admit that using more rats in vivo would make the conclusion more reliable. Unfortunately, the project was delayed due to funding issues. We are currently facing significant resource constraints: reductions in research funding from the National Natural Science Foundation have severely limited our funding for reagents and animal experiments in this study. As a result, it has become impossible to fully repeat all animal experiments at the original scale and quality in the short term. Regrettably, to supplement the NRF2 knockout animal-related experiments (n=6), we have already spent approximately 70,000 RMB (about 10,000 USD). We have made tremendous efforts to ensure the scientific rigor of the paper. We sincerely apologize for any inconvenience caused. At the same time, we fully recognize the importance of increasing the sample size in animal experiments for this study. We have explicitly acknowledged this as a limitation of our work in the Discussion Section and have revised the manuscript accordingly. We greatly appreciate your understanding.

(4) Furthermore, please double-check the blot in Fig. 9D. Tubulin and P-p65 bands appear very similar, and tubulin disappears in response to EtOH and EtOH/CAP treatment in KO mice. Is it the case? I am not sure the quantitative data reflect the WB bands. Please verify that.

We sincerely appreciate your valuable feedback on our manuscript. Indeed, we may have included bands that do not meet the requirements due to our eagerness, and we are very grateful for your pointing this out; it was indeed a significant oversight on our part. I will definitely pay more attention to careful checking in the future. In response to this, we have re-conducted the experiments using the preserved tissue samples and have accordingly updated Figure 9d. Thank you for your insightful suggestions.

**Reviewer #2 (Recommendations for the authors):**

Presentation:

The data with the encapsulated CAP appear a little as side arm that does not bolster your main message (maybe take out and elaborate on this topic more extensively in another manuscript)

We sincerely thank the reviewer for this suggestion. However, based on the ELISA results demonstrating that nano-capsaicin exerts a significantly stronger anti-inflammatory effect than pure capsaicin (CAP), and considering its superior sensory profile for industrial applications (confirmed by our sensory evaluations), we believe these data provide valuable insights. Therefore, we would prefer to retain this section in the manuscript and hope for your understanding.

Revise the introduction on the Nrf2 signaling pathway ...as it is written at the moment, someone outside the Nrf2 field might have trouble to understand

Thank you for the valuable suggestion again. We have rewritten the introduction to the NRF2 signaling pathway to improve accessibility for readers outside the field.

“The Kelch-like ECH-associated protein 1 (KEAP1)–Nuclear factor erythroid 2–related factor 2 (NRF2)–antioxidant response element (ARE) pathway is a core defense mechanism against oxidative and electrophilic stress[11]. Under homeostatic conditions, KEAP1 acts as a linker protein for the Cul3-E3 ubiquitin ligase complex, continuously promoting the ubiquitination and proteasomal degradation of NRF2, thereby maintaining NRF2 at basal levels[12]. When oxidative or electrophilic stress occurs, critical cysteine residues in KEAP1 are modified, or the interaction between the ETGE/DLG motifs on NRF2 and the Kelch domain of KEAP1 is disrupted, allowing NRF2 to escape degradation, accumulate, and translocate to the nucleus. There, NRF2 forms heterodimers with small Maf proteins and binds to ARE, inducing the expression of antioxidant and cytoprotective genes such as those involved in glutathione metabolism, NADPH regeneration, phase II detoxifying enzymes, and drug efflux transporters, thereby restoring redox balance within the cell and reducing oxidative damage[13].

Classical NRF2 agonists, such as sulforaphane, are small molecules that bind to KEAP1 and covalently modify its cysteine residues, thereby altering the binding affinity between KEAP1 and NRF2 [14]. However, traditional covalent agonists may induce sustained overactivation of NRF2, leading to adverse side effects and limiting clinical application [15]. Consequently, recent efforts have shifted toward the development of non-covalent NRF2 agonists, which are generally associated with lower toxicity and greater translational potential, enabling more controlled enhancement of NRF2 activity and offering new insights and therapeutic opportunities in antioxidant-related interventions.”

The authors should check and review extensively for improvements to the use of English to get rid of awkward phrases /wording.

Thank you very much for this helpful comment. We sincerely appreciate the suggestion and have carefully re‑read and further polished the entire manuscript to remove awkward phrasing and improve the readability of expressions and phrases. We hope these revisions address your concern, and we remain grateful for your guidance.
